# Unobserved confounders cannot explain over-crediting in avoided deforestation carbon projects

**DOI:** 10.1038/s41559-026-03049-7

**Published:** 2026-05-07

**Authors:** Alejandro Guizar-Coutiño, George Nicholson, David Coomes, Paul J. Ferraro, Tom Swinfield, Julia P. G. Jones

**Affiliations:** 1 Department of Plant Sciences, University of Cambridge, Cambridge, UK; 2 Department of Biology, University of Oxford, Oxford, UK; 3 Department of Statistics, University of Oxford, Oxford, UK; 4 Department of Environmental Health and Engineering, Johns Hopkins University, Baltimore, MD USA; 5 Carey Business School, Johns Hopkins University, Baltimore, MD USA; 6 Department of Zoology, University of Cambridge, Cambridge, UK; 7 School of Environmental and Natural Sciences, Bangor University, Bangor, UK; 8 Department of Biology, Utrecht University, Utrecht, the Netherlands

**Keywords:** Environmental impact, Conservation biology, Environmental economics, Climate-change mitigation, Ecosystem services

## Abstract

In ecology and conservation, a growing number of studies seek to draw causal inference using quasi-experimental designs. Despite the risk of omitted variable bias from such designs, the degree to which results are sensitive to unobserved confounders is seldom assessed. Here, to demonstrate the value of such sensitivity analyses, we use the controversy surrounding whether projects aimed at reducing emissions from deforestation and forest degradation (REDD+) overestimated their effectiveness (resulting in too many credits being sold). Verifiers of REDD+ credits have argued that independent quasi-experimental analyses of REDD+ projects are flawed because they omit site-specific drivers of deforestation. If these drivers also affect where REDD+ projects are established (that is, projects target areas facing threat), omitting them will tend to underestimate the deforestation that projects avoided. We revisit a global sample of 44 REDD+ projects and show that while some projects reduced deforestation, over-crediting was rife. Crucially, we explore the sensitivity of these results to unobserved confounders and demonstrate that unobserved local drivers of both deforestation and REDD+ locations are unlikely to fully account for reported over-crediting. Assessing sensitivity to unobserved confounders remains uncommon in ecology and conservation but should become standard practice where causal conclusions are based on controlling for confounders.

## Abstract (web summary)

Causal inference should quantify the sensitivity of results to hidden confounders (variables affecting both the location of projects and outcomes). The importance of this is illustrated using analysis of over-crediting in REDD+ projects.

## Main

In ecology and conservation science there is increasing interest in exploring causal relationships where, for logistical or ethical reasons, experiments are not possible^1,2^. For example, unpacking the effect of biodiversity on ecosystem functioning at scale^3,4^ and quantifying the effectiveness of conservation interventions^5–7^ both require causal inference from observational data. Conditioning on confounders (variables which influence both the outcome and exposure to the treatment) is a widely used study design^1,8^. A crucial assumption of such designs is that there are no important unobserved confounders (that is, no ‘omitted variable’ bias). Yet, studies seldom explore how their results would differ if this assumption were violated^9^.

The challenge of omitted variables has been understood since the foundational work of Cornfield et al.^10^, who developed methods for placing bounds on the influence of unobserved confounders when exploring the causal relationship between smoking and lung cancer. One of the world’s leading statisticians of the time, R. A. Fisher, argued that the observed association between smoking and cancer was caused by a shared genetic predisposition to both. The insight of Cornfield et al.^10^ was to demonstrate that for an unobserved confounder to fully explain smokers’ nine-fold greater risk of developing lung cancer compared to non-smokers, the confounder would have to be nine-fold more prevalent among smokers. Since then, numerous sensitivity analysis techniques have been developed^9–14^. However, many studies in ecology and conservation do not apply such tests^15,16^.

We revisit the heated debate about over-crediting in the voluntary carbon market for REDD+ carbon credits^17,18^. REDD+ credits are issued by projects claiming to avoid carbon emissions by conserving tropical forests that would otherwise have been destroyed. However, the extent to which REDD+ projects have provided genuine forest conservation has come under intense scrutiny^19^. Independent analyses carried out by researchers without links to projects or verification bodies, using quasi-experimental methods that account for observed confounders, suggest that many first-generation REDD+ projects delivered little. For example, Guizar-Coutiño et al.^20^ found that 33 out of 40 projects avoided little or no deforestation, while two high-profile studies^21,22^ showed that projects have often issued more carbon credits than could be justified. The resulting scandal^18^ contributed to US$1 billion being wiped off the value of the voluntary carbon market^23^.

**Fig. 1:**
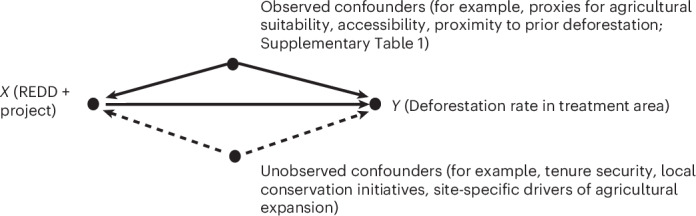
Simple directed acyclic graph showing the relationship between a REDD+ project and the outcome of interest (deforestation). Observed confounders can be accounted for in a study design, but unobserved confounders may remain. Failing to control for confounders that are positively associated with the treatment (location of REDD+ projects) and negatively associated with the outcome (deforestation) will tend to overestimate the effect of the treatment, whereas failing to control for confounders that are positively associated with both will tend to result in underestimates of the treatment effect. Exploring the sensitivity of estimated treatment effects to unobserved confounders is therefore important to check the reliability of causal inference from observational data, but it is not widely done in ecology or conservation.

Many in the carbon credit industry strongly refute the findings^24–26^. One important critique of these studies is that they inevitably miss important factors that may influence deforestation at the local level, such as the land tenure or emerging local pressures. If these local drivers of deforestation are also associated with where REDD+ projects are established, omitting them from an analysis could indeed underestimate the impact of REDD+ projects. In other words, although the published evaluations of REDD+ projects control for observed confounders, they may miss unobserved confounders that are positively associated with both deforestation and where REDD+ projects are established (Fig. 1).

**Fig. 2:**
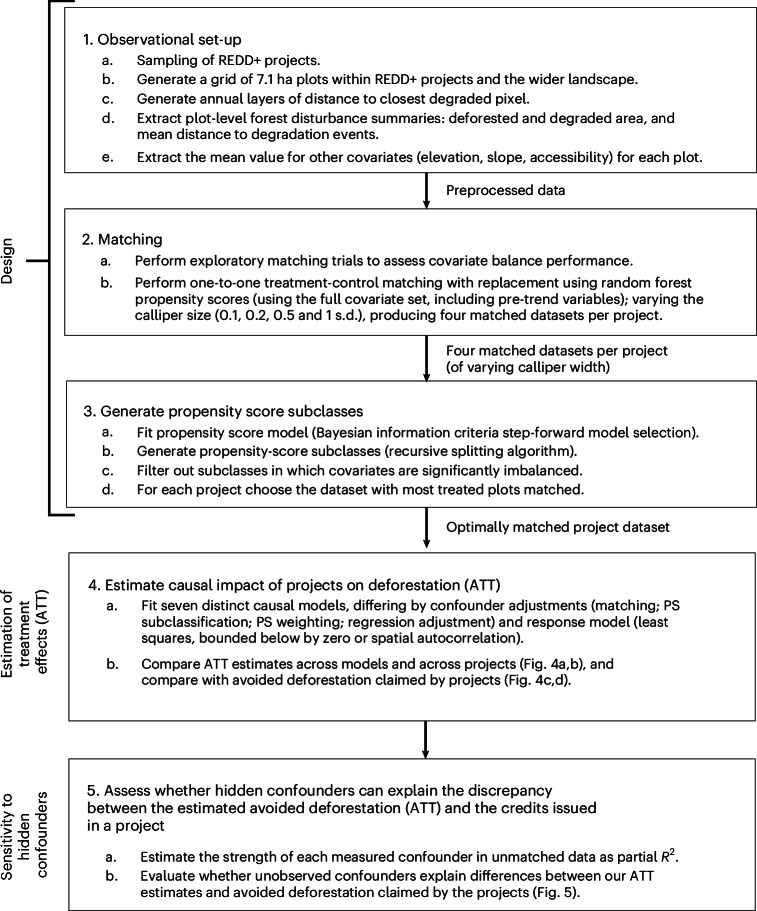
Schematic of the pipeline used in the analysis. Blocks indicate components of the analysis and are described in Methods (with more detail in Supplementary Information). PS, propensity score.

Although the REDD+ methodologies used in the global-scale analyses of REDD+ projects are being phased out and replaced by jurisdictional REDD+ approaches^27^, there are important lessons to learn from reanalysing the data that contributed to claims of over-crediting (Fig. 2). First, we quantify the extent of over-crediting by comparing the avoided deforestation claimed by the projects in certified reports with improved estimates of avoided deforestation from one of the independent studies^20^. Improvements in the prior estimates came from two methodological changes aimed at reducing potential sources of bias: sampling deforestation using plots rather than pixels^28^ and contrasting multiple estimates from causal inference estimators that more comprehensively control for sources of confounder bias (for example, matching with propensity score subclassification, covariate-adjusted estimation and fixed-effects panel analyses). Then we show how sensitivity analyses^29^ can determine the extent to which unobserved confounders in our designs could explain the difference between our estimates and the certified estimates of avoided deforestation from project documents (specifically, monitoring and evaluation reports^30^). We use this case study to illustrate the wider point about the importance of exploring the sensitivity of results in any causal analysis to the influence of potential unobserved confounders.

## Results

### Avoided deforestation

**Fig. 3:**
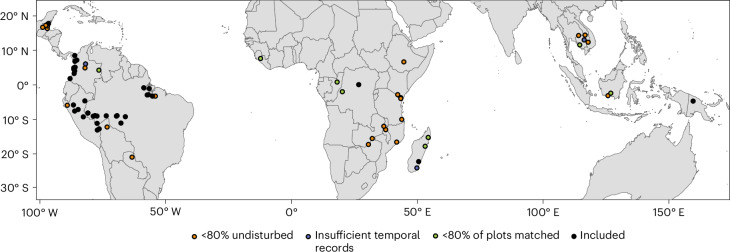
Location of the REDD+ projects included in the analysis. Of the 71 mapped sites, 22 were excluded because they had less than 80% undisturbed evergreen or semi-evergreen forest cover at the start of the project (orange), and 5 were excluded for not meeting our timeframe criteria. This leaves 44 projects that were examined (34 in the Americas, 7 in Africa and 3 in the Asia-Pacific). Of these sites, 8 were excluded because matches could be found for <80% of treatment plots (green; results for these projects are included in the Supplementary Information). After these exclusion criteria were applied, we are left with 36 projects (black) for our main analysis.

**Fig. 4:**
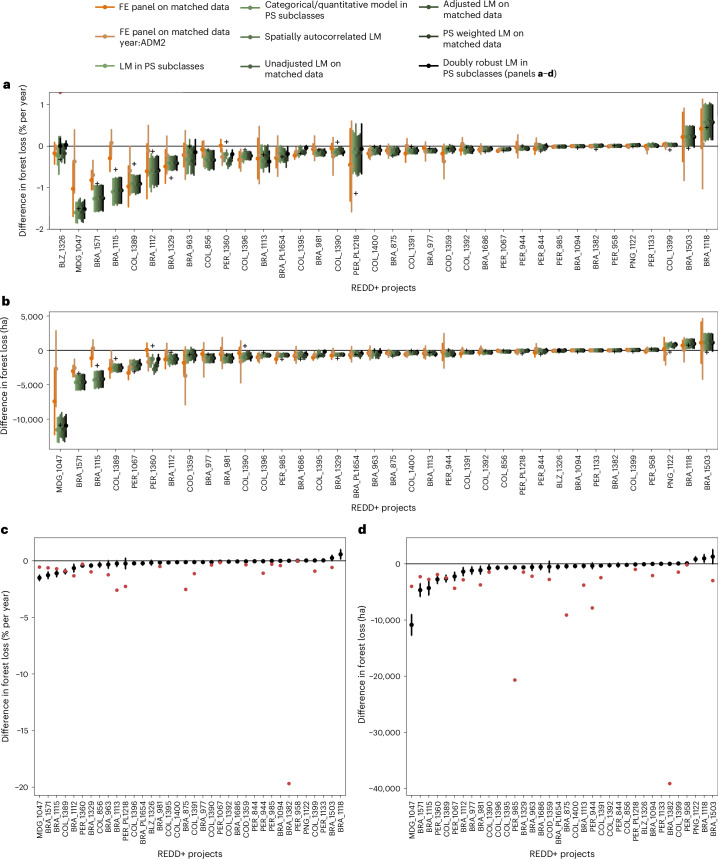
Treatment effects for robustly matched REDD+ projects. **a**,**b**, Estimates show differences in deforestation rates (**a**) and differences in the area of forest lost (**b**) for the 36 REDD+ projects, which were robustly matched, across all model specifications. Estimates below the zero line indicate avoided deforestation; error bars represent ±2 standard errors. The ATT from ref. ^20^ is marked with a ‘+’. **c**,**d**, ATT estimates from the doubly robust linear model (LM) using propensity score subclassification, in terms of deforestation rates (**c**) and forest area lost (**d**). Claimed avoided deforestation by projects is represented by red dots and is available for the 23 projects included in the sensitivity analysis (Fig. 5). See Supplementary Fig. 4 for the results from all 44 projects, including those with <80% of plots matched. Project codes are given in Supplementary Table 1. ADM2, second order administrative units; FE, fixed effects.

After filtering, 36 REDD+ projects were included in our main analysis (33 in the Americas, 2 in Africa and 1 in the Asia-Pacific) (Fig. 3 and Supplementary Table 1). These projects varied in how much deforestation they avoided over their first five years of operation, both in terms of relative reductions in forest loss (Fig. 4a) and absolute deforestation avoided (Fig. 4b). Across our nine estimators, our project-level estimates of average treatment effect on the treated (ATT) are consistent in their qualitative pattern (Fig. 4). Although some projects have effect sizes that suggest that they delivered substantial avoided deforestation (those to the left-hand side of each panel in Fig. 4), most have effect sizes close to zero. Estimates varied across models, but the overall ranking of projects was remarkably stable. This concordance indicates that our conclusions are not driven by a single modelling choice but are robust across regression adjustment, matching-only approaches, propensity score subclassification, weighting estimators and fixed-effects panel models.

The fixed-effects panel estimators produced ATT estimates that, with few exceptions, were similar to those from our matched cross-sectional models but with wider confidence intervals (Fig. 4). In cases where the panel and cross-sectional estimates differed substantially, the panel estimate was closer to zero in most cases, indicating that additional control for time-invariant confounding does not reveal systematically larger project impacts.

Our new panel estimates also help explain the relationship between our cross-sectional results and those presented previously^20^. The earlier analysis was not a conventional cross-sectional approach: by restricting the sample to plots with zero pre-treatment loss, ATTs were identifiable through within-plot temporal change. This structure is conceptually very close to a fixed-effects panel model. Accordingly, our new panel estimates align closely with the earlier paper, whereas our new cross-sectional estimators (which match on a broader set of time-invariant predictors rather than imposing a flat pre-treatment baseline) yield different effect sizes for some projects, such as PER_1360 (Fig. 4).

The 36 site-level estimates of avoided deforestation reveal a mean reduction of 0.26% per year (95% confidence interval (CI) 0.16–0.42% per year) in the rates of forest loss compared to controls. This is essentially unchanged from the figures presented in ref. ^20^, which calculated a mean reduction of 0.22% per year (95% CI 0.13–0.34% per year) across examined projects. A Bland–Altman analysis indicated negligible bias between studies (mean difference = −0.01, 95% CI −0.11 to 0.08), with limits of agreement ranging from −0.56 to 0.54 (Supplementary Fig. 1). Combining the 36 site-level estimates, we observe a reduction of deforestation of 0.62% of the total REDD+ area examined, amounting to approximately 33,948 ha.

For the 23 out of 36 projects for which we have comparable data on certified avoided deforestation, our results suggest substantial over-crediting. (However, it is important to note that five projects, one in Madagascar, two in Brazil, one in Peru and one in Colombia, under-credited—that is, they claimed less deforestation than our results suggest they avoided; Fig. 4c,d.) Overall, the projects for which this comparison is possible claimed 3.9 times more avoided deforestation in evaluation and verification reports compared with our estimates (~123,099 ha compared to ~31,822 ha), of which about 50% is explained by two projects alone: BRA_1382 and PER_985, with estimated claimed avoided deforestation areas of 39,122.2 ha and 20,684.2 ha, respectively.

### The influence of unobserved confounders

To assess whether unobserved confounders could plausibly explain discrepancies between our estimated avoided deforestation and the amounts certified by projects, we quantified the potential strength of unobserved confounding using partial *R*^2^. For each project, we computed the partial *R*^2^ of each measured confounder (distance to degradation, mean slope, mean accessibility and mean elevation) with treatment assignment and with deforestation outcomes, using unmatched data (Supplementary Fig. 2). These statistics represent the proportion of residual variation explained by the measured confounders, conditional on all other covariates, and were used as empirical benchmarks. We then parameterized hypothetical unobserved confounders with partial *R*^2^ values equal to one, two and three times those of each observed confounders, evaluating each confounder–project combination separately to maintain interpretability. Following ref. ^9^, this analysis quantifies how strong an unobserved confounder would need to be to materially alter our ATT estimates.

**Fig. 5:**
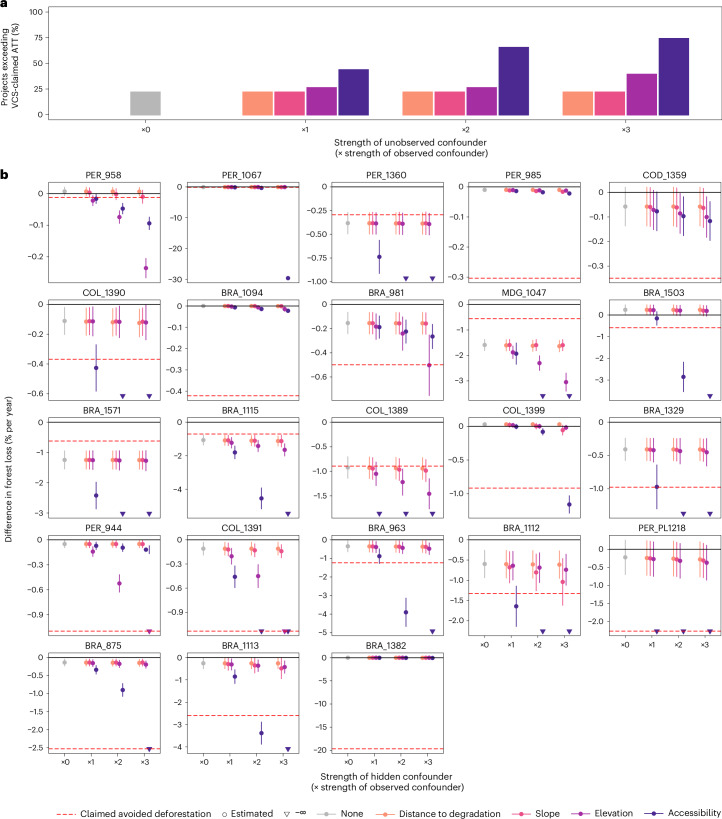
Sensitivity of estimates of avoided deforestation to unobserved confounders. Tests assessed the sensitivity of avoided deforestation estimates to unobserved confounders one, two or three times as strong as each measured confounder considered in this analysis. Results are shown for the 23 projects with available data on the avoided deforestation claimed by the project (projects marked with red dots in Fig. 4c,d). **a**, Percentage of projects that reached or exceeded the avoided deforestation claimed by the projects when ATTs are re-calculated considering unobserved confounders one to three times as strong as measured confounders. **b**, Re-estimated ATT values under varying unobserved confounder strengths, with the red dashed line indicating the avoided deforestation claimed by each project and error bars representing ±2 standard errors. ATT values with out-of-range estimates (−∞) are shown as triangles. See Supplementary Fig. 5 for results including projects where <80% of treated plots were successfully matched. Project codes are shown in Supplementary Table 1. VCS, Verified Carbon Standard.

The level of unobserved confounding required to align our results with certified avoided deforestation would need to be implausibly strong, making it unlikely that unobserved confounders alone account for the systematic over-crediting observed. Accessibility (mean travel time to the nearest urban centre) is consistently the strongest observed confounder. It shows partial *R*^2^ > 0.25 with treatment assignment in 14 out of 44 projects, compared with 4 for elevation, 1 for slope and none for distance to degradation. Associations with deforestation outcomes are substantially weaker: no project–covariate combination shows partial *R*^2^ > 0.10 after conditioning on treatment. These results are consistent with established ecological evidence that remote areas tend to face lower deforestation pressure and are more likely to be selected for conservation interventions^31^. Using these empirical values as benchmarks (Fig. 5), we find that in 5 of the 23 projects with certified avoided deforestation data, our estimates already exceed the amounts credited without invoking any unmeasured confounding. A hypothetical unobserved confounder as strongly associated with both treatment and outcome as accessibility would reconcile the remaining discrepancies in only five additional projects. Under highly conservative scenarios, where such a confounder is assumed to be twice or three times as influential as accessibility, this increases to 15 or 17 projects, respectively. No variable of this magnitude has been proposed in the REDD+ literature or identified empirically.

## Discussion

In ecology and conservation science, most causal relationships are embedded in complex social-ecological systems, which makes quantifying these relationships challenging^5^. Owing to the ever-present risk posed by unobserved confounders in these systems, ecologists have traditionally viewed causal inference from observational data with scepticism^3^. One effective approach for adjudicating debates about the influence of unobserved confounders in a study is to ask how strongly an unobserved confounder would need to be associated with both the treatment and outcome variables to change the study’s findings^9,15^. We demonstrate a practical application of this approach by revisiting a critical debate in applied ecology and conservation: did REDD+ forest carbon projects systematically issue more credits than justified?^22^ This question is of importance to scientists who care about the future of tropical forests and the search for solutions to stem their losses.

Reanalysing data from an evaluation of a global sample of REDD+ projects^20^, we find that unobserved confounders are unlikely to account for the discrepancy between the estimates of avoided deforestation in scientific publications and the avoided deforestation claimed by the projects. This observation is important because industry-led critiques of the independent academic-led estimates of avoided deforestation from REDD+ have argued that such estimates fail to account for key local drivers of deforestation^24,25^. If such drivers are also positively associated with where REDD+ projects are located, omitting them would result in underestimates of avoided deforestation.

For most REDD+ projects in our sample, if such confounders were to explain the discrepancy between academic-led estimates of avoided deforestation and the avoided deforestation claimed by the projects, they would have to be much stronger than the largest observed confounder, which in most cases was ‘accessibility’. For the projects with the greatest discrepancies, even an unobserved confounder three times stronger than accessibility could not explain the discrepancy.

Although it is impossible to know the magnitude of unobserved confounders relative to those we observe, our analysis suggests it is unlikely that unobserved variables could explain the widespread discrepancies between estimated and claimed deforestation reductions. Accessibility—by far the strongest observed confounder—is a well-established determinant of both deforestation risk and project placement^31,32^. Yet, even a hypothetical unobserved confounder one, two or three times as strongly associated with both treatment and outcome as accessibility would still, respectively, leave 13, 8 and 6 projects out of 23 with smaller estimated impacts than claimed. Although unobserved confounders such as tenure security, the presence of local conservation efforts or agricultural pressures^32^ may explain discrepancies in a few projects, they are unlikely to systematically account for the scale of over-crediting observed.

There are a variety of approaches for exploring the sensitivity of estimates of treatment effects to unobserved confounders. We considered alternative approaches that have been proposed for observational or panel settings, including methods for two-period panel estimators such as in ref. ^33^, Oster-type bounds^34^, *E*-values^35^ and several classical frameworks^12,36,37^. These approaches are not well suited to our design or data structure, and they produce sensitivity measures that are less interpretable in ecological applications (refer to Supplementary Information for a full explanation). For these reasons, we adopt the framework presented previously^9^, which avoids strong parametric assumptions, provides formally valid bounds grounded in omitted-variable bias theory and, crucially, produces sensitivity measures directly interpretable relative to the strength of observed confounders.

There are, of course, important caveats to this work. First, our analysis covers only a subset of the REDD+ projects that were active at the time (Supplementary Tables 1 and 5). We could only estimate avoided deforestation for the projects for which there were sufficient temporal records, sufficient coverage of undisturbed moist forest at baseline and for which we could match at least 80% of treated plots with control plots (36 out of 71 projects). Further, we could only explore whether unobserved confounders could explain over-crediting in the subset of 23 of these 36 projects for which we could obtain data on how much avoided deforestation was claimed by the projects in their project monitoring and evaluation reports^30^. If the included projects systematically differ from the excluded projects, this constraint could limit the generalizability of our conclusions. Second, confounders are not the only potential source of bias in the independent studies. Model misspecification biases^38^ could also account for the discrepancy between the certified estimates of avoided deforestation and those from the independent studies. Third, spillovers, also referred to as interference among units, could also affect inferences in the independent study designs^5^, but such spillovers would be more likely to inflate these estimates of avoided deforestation rather than deflate them because REDD+ projects are more likely to displace deforestation outside the project area than to reduce it. Fourth, all approaches to sensitivity analyses rely on assumptions that may be violated in empirical contexts. Most of the assumptions underlying the Cinelli and Hazlett^9^ approach that we use are plausible in our study context, but our conclusions about sensitivity to unobserved confounders could change if the effect of the observable and unobservable confounders have non-linear relationships with the treatment and outcome variables or if the confounders interact with each other.

Our results, which account for the influence of unobserved confounders in REDD+ project evaluations, add to the overwhelming body of evidence showing that first-generation REDD+ projects did, indeed, issue more credits than justified^22,39^. However, while over-crediting was rife in first-generation REDD+ projects, our results suggest that they did, on average, slow deforestation. Across the 36 projects for which we have reliable data, the projects avoided 33,948 ha of deforestation. While the voluntary carbon market is not the only means of financing forest conservation, selling emissions reductions from avoided deforestation can generate hard-to-replace finance and could make a valuable contribution in the short-to-medium term for conserving tropical forests and mitigating greenhouse gas emissions^17,18,40^. However, for such credits to be reliable they must be based on conservative, ex-post counterfactual deforestation estimates (that is, how much deforestation would have occurred without the REDD+ investment)^41^. Although the new jurisdictional REDD+ methodologies (such as ART TREES and VM48) avoid some of the limitations of first-generation projects by taking responsibility for setting crediting baselines out of the hands of project developers^42^, they do not yet include such dynamic baselines. A move to dynamic baselines would not change the timing of credit issuance (as existing methodologies only issue credits after ex-post assessment of deforestation in project areas). They would, however, increase uncertainty in the number of credits a project developer might expect^43^.

Although this paper has delved into a specific case where putting bounds on the likely influence of unobserved confounders is useful we wish to make a broader point which goes beyond this specific case study. The importance of accounting for the potential influence of confounders when estimating causal effects from observational data has been recognized since the 1950s^10,44^, and there have been examples that apply these ideas to impact evaluations in ecology and conservation for many years^45^. However, the application of these approaches remains far from universal. New packages^29^ simplify these tests, and we argue that sensitivity analyses should become standard practice whenever ecologists and conservation scientists seek to draw causal inferences from observational data.

## Methods

Our goal is to estimate the causal effect of REDD+ projects by comparing the deforestation within project boundaries with the deforestation which would have occurred in the same area of land had the project not occurred. We can frame this goal as a standard causal inference problem, where ‘treatment’ corresponds to ‘land included in a REDD+ project’. The ATT is the arithmetic difference of average deforestation of the REDD+ project implementation area minus the average deforestation of the same area of land in the counterfactual scenario of no REDD+ project. The analytical pipeline is shown in Fig. 2. We are faced with the usual challenge that treatment allocation (REDD+ area designation) may depend on certain area characteristics, such as pre-treatment deforestation trends or land accessibility, which may also affect the outcome (deforestation rate).

Our outcome of interest is deforestation as reported in the tropical moist forest (TMF) maps, which present the deforestation and degradation of evergreen and semi-evergreen forest areas across the humid tropics at 30 m resolution from 1990 to 2019^46^. While many analyses of the impact of conservation interventions on deforestation use pixels as the unit of analysis, there is recognition that analysing deforestation as binary, unrepeatable data (whether a pixel is deforested or not) can result in biases that are reduced by aggregating binary pixels spatially^28^. Where appropriate polygons exist, the unit of analysis can be pixels aggregated within land holdings^47^ or administrative boundaries^48^. However, appropriate polygons are not always available with which to sample the landscape. We sample deforestation from treatment (REDD+ projects) and control (the wider forested landscape) using plots with a 150-m radius (7.1-ha plots), defining our outcome of interest as the plot-level percentage of forest loss (ignoring regrowth) relative to the plot-level forested area observed prior to REDD+ implementation.

### Observational set-up

#### Selection of REDD+ projects

We used the database of REDD+ projects compiled previously^20^, which identified 81 REDD+ projects established in the tropics^49^ and successfully found boundary data for 71 of these. While new projects have been introduced since 2019, and others withdrawn, for consistency with ref. ^20^, we analyse the same set of projects. Guizar-Coutiño et al.^20^ aimed to include sites that encompassed at least 80% of the ‘undisturbed’ forest class as characterized by the TMF database at the project start date, which enabled the identification of standing forest areas from at least 1990 until the project start date. The selection criterion was implemented to ensure alignment with REDD+ protocols from the Verified Carbon Standard^49,50^, which require that a project’s spatial extent encompasses land that has maintained 100% forest cover for at least ten years prior to the project starting date. The TMF database, whilst constrained to the moist tropics, enables per-pixel tracking of forest trajectories, distinguishing long-standing forests from areas that have been recently degraded or deforested, which enabled the identification of undisturbed forested areas that could be used as potential controls to assess REDD+ effectiveness. We excluded 22 projects with <80% undisturbed forest cover by the time of project implementation (Supplementary Table 4). Projects that had been operating for fewer than five years or began before 2000 were also excluded, in the latter case because these started before REDD+ was established as an international framework. The remaining 44 REDD+ projects were taken forward for subsequent analyses. Supplementary Table 5 shows how the characteristics of the included projects differ between the 71 projects to the subset of 44.

#### Sampling within REDD+ areas and forested landscapes

To assess the extent of reductions in deforestation due to REDD+, we compared changes in forest cover within treated areas with those taking place in similar forest patches within the wider landscape. We generated plots with a radius of 150 m on a 1-km grid within forested areas of the REDD+ projects and surrounding regions. Each plot was ~7.1 ha in size and contained up to 78 TMF pixels, which we considered adequate for characterizing local drivers of deforestation and our outcome as continuous variables^51^. We used the extent of forest cover in 1990 as delineated by the TMF to position sampling plots, ensuring that all treated samples contained at least some portion of undisturbed moist forest cover (excluding plots that have been entirely degraded or deforested) when building the time series. We used the same approach to sample TMF plots in the wider landscape: we removed sampling locations that were not forested or that lay within a 15-km buffer zone around the project area, which may have been affected by local leakage of economic activities^52^. To create a pool of sample plots from which to find matches for REDD+ project plots (Supplementary Fig. 3), we random subsampled from this set until we had up to five samples in the wider landscape for each sampling plot located inside the REDD+ project, each separated by at least 1 km to reduce spatial dependence^53^.

#### Plot-level estimates of forest cover, forest degradation and deforestation

Annual estimates of forest cover, forest degradation and deforestation were extracted from the TMF database. This database provides a long-term characterization of forest disturbances in the humid tropics on an annual basis spanning the period 1990–2019. Our analyses focused on examining the temporal patterns of three main forest classes: the ‘undisturbed’ class, which refer to closed evergreen or semi-evergreen forest areas that have not been degraded or deforested since at least 1990; the ‘degraded’ class, consisting of short-term disturbances (less than 2.5 years) due to anthropogenic causes, such as selective logging, or from natural causes such as wind storms or fires; and the ‘deforested’ class, representing a long-term removal of forest cover^46^. For each year in the 1990–2019 period covered in TMF’s Annual Change Collection series, we calculated the extent of undisturbed, degraded and deforested forests classes within sampling plots. We derived our outcome of interest—the annual percentage of forest cover lost—as the percentage of undisturbed forest cover loss relative to the extent of undisturbed forest cover six years prior to project implementation. Finally, we generated annual plot-level estimates of proximity to forest degradation events, as characterized by TMF’s degraded forest class. Additional details on the generation of plot-level forest metrics are provided in Supplementary Information, ‘Observational set-up’.

#### Confounders

We identified key confounders likely to be associated with deforestation^32^ and where REDD+ projects are assigned in a landscape^54^ (Supplementary Table 2). Many of these are positively or negatively associated with deforestation—for instance, distance to recent forest clearings and accessibility^32^—while others are likely to be positively or negatively associated with treatment (location of REDD+ projects). If we are missing confounders that are positively associated with both, then we would be overcorrecting the bias, making REDD+ projects look less effective, on average, than they are. Additional details on the generation of confounder estimates are provided in Supplementary Information, ‘Observational set-up’.

#### Matching

Before settling on a matching strategy, we explored how different choices during statistical matching impacted the covariate balance between treated and control samples, and the proportion of project-area plots that were matched (Supplementary Information, ‘Exploratory matching analysis’). Based on this analysis, we conducted matching experiments for the 44 projects identified in the ‘Selection of REDD+ projects’ section (Fig. 3) as follows.

#### Distance metric

Propensity scores were estimated using a random forest classifier (random forest matching). These predicted probabilities of treatment assignment were used as the distance measure for matching^55^.

#### Observed confounders used in matching

We used the full set of observed confounders (based on known key drivers of deforestation^32^; Supplementary Table 2). These include elevation, slope, accessibility (measured as travel time to cities) and distance to recent forest degradation events in the five years before project start. We also collected key characteristics at the plot level, such as forest disturbance trends measured over both short-term (year −6 to year 0) and long-term (1990 to year 0) periods, which encompassed the extent of degraded and deforested forest as characterized by the TMF’s degraded and deforested forest classes (class 2 and 3, respectively), provided in relative and absolute terms. We also included measures of historical undisturbed forest area for year −6 and in 1990.

#### Replacement

We performed one-to-one matching with replacement, allowing control units to be matched to multiple treated units.

#### Callipers

To remove poor quality matches, where quality is defined as the magnitude of the differences in covariate balance between treated plots and their matched controls, we applied calliper widths of 0.1, 0.2, 0.5 or 1 standard deviations. Smaller widths result in smaller differences between treated and control plots, at the expense of dropping observations and, thus, restricting the generalizability of the results (that is, we trade-off external validity for greater internal validity). This variation in calliper width resulted in four matched datasets per project, which we take forward to the next stage.

#### Propensity score subclassification

To increase comparability between treated and control plots prior to estimating post-implementation deforestation differences, we applied propensity score subclassification^56^ within the matched datasets, grouping plots with similar treatment probabilities based on pre-treatment characteristics. Subclasses were defined using logistic regression-based propensity scores and recursive balance diagnostics, ensuring covariate alignment between treated and control observations before estimating cross-sectional ATT. Only projects in which at least 80% of treated plots could be successfully subclassified were retained, with full procedures detailed in Supplementary Information.

From the 44 sites examined, 36 sites were included in the main analysis (33 in the Americas, 2 in Africa and 1 in the Asia-Pacific). Successfully matched projects (*n* = 36) did not differ systematically in covariate values relative to projects that did not meet the 80% treated sample criterion (*n* = 8), except that matched projects experienced more deforestation in the run up to REDD+ implementation, which could indicate an overall greater exposure to drivers of deforestation compared to excluded projects (Supplementary Table 3).

### Estimating causal effect of projects on deforestation (ATT)

To estimate the ATT and assess its robustness to modelling assumptions, we fit and compare a range of causal models that differ in both statistical specification and in the assumptions they use to identify causal effects. Most of our estimators are cross-sectional, comparing post-implementation deforestation between matched treated and control plots, using approaches such as matching, propensity score subclassification, covariate adjustment and weighting. These methods rely on a selection-on-observables assumption: once we condition on observed confounder, treated and control plots are assumed comparable.

To complement these cross-sectional estimators, we additionally fit two fixed-effects panel models. The first uses plot and time fixed effects to allow for unobserved but time-invariant confounders, and identifies effects using only within-plot temporal variation. This specification relies on the standard parallel-trends assumption that, in the absence of treatment, each plot’s deforestation trajectory can be represented as the sum of a plot-specific baseline level and a shared time trend. The second panel model includes fixed effects for plot and time, as well as an interaction term between time and sub-national geographic units (ADM2), as defined in the geoBoundaries database^57^, which controls for any time-varying confounders at the level of these administrative unit (such as counties, districts, municipalities or regions). Our primary purpose in fitting multiple models is to examine the stability of ATT estimates to potential violations of statistical modelling assumptions, such as sensitivity to the choice of modelling strategy, unaccounted spatial dependence or non-standard outcome distributions (for example, deforestation bounded below by zero). By fitting models based on panel data, regression adjustment, propensity score weighting, matching alone, propensity score subclassification and a doubly robust approach, we also gain insight into the practical performance of different causal estimation strategies under real-world conditions. Although not doubly robust in the classical sense, regression adjustment after propensity score subclassification can reduce bias by adjusting for residual covariate imbalance within subclasses. Comparing results across these models helps highlight where ATT estimates are sensitive to specific modelling assumptions, which is valuable for evaluating the stability and credibility of our findings. Full implementation details are provided in Supplementary Information.

### Evaluating whether unobserved confounders explain differences between our ATT estimates and avoided deforestation claimed by the projects

To compare our empirical estimates of avoided deforestation with the values reported in Verified Carbon Standard project monitoring documents^29^ we conducted a structured sensitivity analysis following ref. ^9^. This approach quantifies how strong an unobserved confounder would need to be, relative to measured confounders, to meaningfully alter the ATT.

For each measured confounder (distance to degradation, mean slope, mean accessibility, mean elevation), we estimated two partial *R*^2^ statistics using unmatched data: (1) the proportion of variation it explained in treatment assignment after accounting for other observed confounders; and (2) the proportion of variation it explained in deforestation outcomes, adjusting for treatment and other observed confounders. These empirical partial *R*^2^ values provide benchmarks for the strength that a hypothetical unobserved confounder would need to attain to shift ATT estimates. For each project and each confounder separately, we parameterized hypothetical unobserved confounders with strengths equal to one, two and three times the empirical partial *R*^2^ of that confounder. We evaluated each confounder–project pairing individually (rather than pooling across confounders or projects) to avoid obscuring the influence of specific variables.

Sensitivity analysis was implemented using the sensemakr package in R^29^, applied to the matched and covariate-adjusted estimator (model F, Supplementary Information, ‘Estimating causal impact of projects on deforestation (the ATT)’). We selected this estimator as the baseline for three reasons. First, it aligns directly with our core research design, which relies on cross-sectional comparison after matching, while adding robustness through covariate adjustment in the regression stage. Second, it integrates naturally with the Cinelli–Hazlett framework^9^, which quantifies sensitivity in a regression framework in terms of observed covariate strength via partial *R*^2^ metrics. Third, and most importantly, the cross-sectional estimator yields larger (in absolute value) and more precise ATT estimates than the panel estimators, which deliberately gives REDD+ projects the best opportunity to align certified estimates of avoided deforestation with our estimated effect sizes. In contrast to the imprecisely estimated effects that are often closer to zero from the panel data estimators, it would be easier for us to make the claim that unobserved confounders are not the reason why the estimated effects are so much smaller than the certified effects. In other words, the panel estimators are relatively disadvantageous towards project claims and, thus, if we were to use them we could be criticized as having deliberately chosen a model that disadvantages the projects in the sensitivity analysis. Full implementation details are provided in Supplementary Information, ‘Section 6: sensitivity analysis’.

### Reporting summary

Further information on research design is available in the Nature Portfolio Reporting Summary linked to this article.

## Supplementary information

Supplementary Information Supplementary Methods, Supplementary Figs. 1–8 and Supplementary Tables 1–5.

Reporting Summary

Peer Review File

## Data Availability

The data to reproduce the main figures is available at https://osf.io/r9ygh.

## Code availability

The code to reproduce the main figures is available at https://osf.io/r9ygh.
