# Supplementary material for: Unobserved confounders cannot explain over-crediting in avoided deforestation carbon projects
Source: Nat Ecol Evol. 2026 May 7;10(6):1071–81. doi: 10.1038/s41559-026-03049-7 (PMC13253331; doi:10.1038/s41559-026-03049-7)
Supplement: Supplementary file 1 — Supplementary Methods, Supplementary Figs. 1–8 and Supplementary Tables 1–5. [file 41559_2026_3049_MOESM1_ESM.pdf]

nature ecology & evolution

Supplementary information https://doi.org/10.1038/s41559-026-03049-7

Unobserved confounders cannot explain over-crediting in avoided deforestation carbon projects

In the format provided by the authors and unedited

Unobserved confounders cannot explain over-crediting in avoided deforestation carbon projects

Supplementary Methods

1 Selection of REDD+ projects

Sourcing of REDD+ project boundaries. The database compiled by Guizar-Coutiño et al. (2022) was created using project boundaries sourced from the Verra database in 2019, accessed at `http://www.vcsprojectdatabase.org/`, now accessible via `https://registry.verra.org/`. Only projects categorized as 'Reducing Deforestation and Degradation', located in tropical regions (Africa, South-East Asia, Latin America, and Oceania) were included, of which 81 projects were listed at the time. Project boundary data was available for the majority of projects in a KML format. The authors contacted project developers directly when either the project boundary file was missing from the database or when the boundary available online did not correspond precisely with that shown in the Project Design Document (PDD). Project boundaries obtained directly from developers included BRA_1118, BRA_1329, and COD_934, leading to a total compilation of 71 boundary files out of the 81 listed projects in the database.

Selection of projects based on tropical moist forest pixel coverage. To characterize forest cover and forest dynamics we use the Tropical Moist Forest (TMF) maps, which present deforestation and degradation across the humid tropics at 30 m resolution from 1990 to 2019. The 71 REDD+ sites for which boundary data was available encompassed areas of moist and seasonally dry tropical forests, but because the TMF database only includes forest change data for evergreen and semievergreen forest, our analyzes are limited to these forest types. We only include projects which had at least 80% cover of *undisturbed* forest class in the TMF dataset (i.e., evergreen/semievergreen forest that has not been degraded or deforested) by the project start date. This excluded 22 projects. Five projects which operated for fewer than 5 years or commenced before 2000 were also excluded. We provide a summary of reasons for inclusion or exclusion in Supplementary Table S1 and detail of forest cover in Table S4.

2 Observational set-up

Longitudinal deforestation data. We derived annual rates of observed changes in forest cover (%) relative to a baseline year prior to REDD+ commencement. Years in each plot are numbered so that treatment occurs in year 0 and data inputs range from year -6 through to year 5. Yearly forest loss, $z_{i,j,t} \in [0\%, 100\%]$, denotes the percentage forest cover lost during year t, in plot j of project i, as a percentage of the estimated forest cover at year -6.

Data preprocessing and quality control. We omit any plots with deforestation exceeding 100% over the entire time course, i.e., with $\sum_{t=-5}^{5} z_{i,j,t} > 100\%$. Measured confounders $\boldsymbol{x}_{i,j}$ are scaled and centred to zero-mean and unit standard deviation within each project.

Outcome definition (proportion of forest loss). The quantitative outcome, $y_{i,j}$, is the average annual percentage of deforestation, averaged across years 1 to 5 post-treatment, i.e. $y_{i,j} := \frac{1}{5}\sum_{t=1}^{5} z_{i,j,t}$.

In addition, a categorical outcome, $c_{i,j}$, is used to indicate whether there was any post-treatment deforestation:

$$c_{i,j} := \begin{cases} 1 & \text{if } y_{i,j} > 0 \\ 0 & \text{if } y_{i,j} = 0 \end{cases} \tag{1}$$

Drivers of deforestation (observed confounders). Details of the observed confounders used are given in Table S1. For each plot, we obtained mean estimates for elevation (meters) and slope (degrees) [Jarvis et al., 2008], travel time to the nearest urban center Weiss et al. [2018], area of degraded forests and distance to the nearest forest degradation event, both as characterized by the TMF's *degraded* forest class. Forest degradation in the TMF consists of short-term disturbances (<2.5 years) due to anthropogenic causes, such as selective logging, or from natural causes such as wind storms or fire, which substantially but temporarily alter pixel spectral characteristics [Vancutsem et al., 2021]. To account for distance to closest forest degradation events, we produced plot-level annual estimates of the distance to the nearest degraded pixel. First, for each pixel covered by the circular plot, we computed pixel-level estimates of the distance to the nearest pixel that had changed its status from undisturbed to degraded, during the observed year, for the period 1990-2019. We then produced plot-level annual rolling estimates of the mean distance to degradation events in the previous five years, covering the period 1995-2019. Pre-treatment deforestation trends included variables capturing short-term trends in deforestation, estimated by computing forest loss rates from 6 years before treatment until the year of project commencement, as well as long-term deforestation trends, encompassing the period from 1990 until the year of project commencement.

Selection of Reduced Emissions from tropical Deforestation and Degradation (REDD+) projects. In Guizar-Coutiño et al. [2022], the authors included project PL1748, South Cardamom REDD+ project in Cambodia, in the analyses in error, despite not meeting the minimum number of years for our criteria. We include it here for consistency despite it only running for four years rather than the five years required by our protocol.

3 Matching

3.1 Exploratory matching analysis

We explored a wide variety of matching protocols (see, e.g., Imbens and Rubin [2015], Chapter 15, Desbureaux [2021]) characterising their ability to improve the balance of measured confounders between treatment and control units. Our tests included variations in the specification of matching runs across the following parameters:

- **Algorithms.** We used three matching algorithms: propensity score matching (PSM), Mahalanobis distance matching (MHN), and Random Forest propensity score matching (RFM).
- **Measured confounders.** We used a "base" set of observable confounders for all runs, focused on characterising key drivers of deforestation Busch and Ferretti-Gallon [2023] (Table S1), which included: 1) elevation; 2) slope Jarvis et al. [2008]; 3) mean distance to population centers Weiss et al. [2018]; 4) mean distance to recent degraded areas over the five years prior to project commencement; 5) extent of degraded area at year 0; 6) short-term deforestation trends, based on absolute area deforested and proportional rates based from year −6 through to year 0; and, 7) long-term deforestation trajectories, based on absolute area deforested and proportional rates based from 1990 through to year 0; while exact-matching by country and biome (that is, control plots were selected from moist tropical forests in the same country). We iteratively added the following covariates to that base set: extent of undisturbed forest in the project at year −6 (forest area t−6), extent of undisturbed forest in 1990 (forest area 1990), and extent of undisturbed forest in 1990 and implementation year (forest area t−6 and forest area 1990). This resulted in a total of 4 distinct combinations of measured confounders.
- **Calipers.** We applied calipers of different sizes to constrain the selection of landscape observations prior to matching, so that the distribution of measured confounders lay within 0.1, 0.2, 0.5 and 1 SD of the distribution in the treatment group. These values were selected to span a range from tight to relaxed matching tolerances commonly used in the literature.

- **Matching with and without replacement.** We considered both matching with replacement (i.e., allowing control observations to be paired with multiple treatment units), and without replacement (i.e., constraining one control observation per treatment unit).

The combination of matching criteria resulted in 96 matching runs for each of the 44 selected REDD+ sites, giving a total of 4,224 runs.

We assessed the quality of matching iterations against 2 criteria:

- Covariate balance between treated and control samples. Similarity between treated and control samples was determined by the overlap of the distributions of the covariate values. Matching runs were considered adequate if the absolute standardized mean difference between treated and matched control samples was less than 0.25 across all measured confounders included in the matching trial Stuart [2010].
- Proportion of project-area samples that were matched. Estimates of additionality can be biased unless a high proportion of treatment samples are successfully matched: only then is the "average treatment effect on the treated" estimated accurately. Matching was considered adequate if at least 80% of treatment samples were paired with a control.

3.2 Selected matching algorithms

Based on our exploratory analysis (Figure S6), we implement matching in the main analysis using Random Forest propensity score matching (RFM) with replacement. We further determined to use the full set of measured confounders (base + forest cover in 1990 and at year -6) and four different caliper widths (0.1, 0.2, 0.5, or 1 standard deviation) in the next stage of our analyses. This combination of matching parameters resulted in matched sets that not always attained covariate balance between treated and control samples across all measured confounders (Figure S7). This issue is addressed in ensuing analyses.

4 Generating propensity score subclasses

For each of the four matched datasets in a REDD+ project, we follow these steps to define covariate-balanced propensity score subclasses, based on Imbens and Rubin [2015]:

1. **Estimate propensity scores.** We estimate propensity scores using a logistic regression model, where treatment assignment is regressed on a set of observed covariates. To select an appropriate model while avoiding overfitting, we apply forward stepwise selection using the Bayesian Information Criterion (BIC) as the decision rule, performing this model selection process separately for each project. The candidate model space includes all main effects, two-way interactions, and quadratic terms (Section 13.3, Imbens and Rubin [2015]). The fitted model resulting from this selection process is then used to compute individual-level propensity scores. This logistic regression approach aligns with standard practices for subclassification and complements the Random Forest-based propensity score estimation used for matching in Stage 2.
2. **Define propensity score subclasses.** We partition observations into propensity score subclasses using a recursive splitting algorithm (Section 13.6 and Chapter 17, Imbens and Rubin [2015]). The procedure begins with all observations in a single subclass. We compute the t-statistic for the difference in mean propensity scores between treated and control units. If this t-statistic exceeds a threshold indicating imbalance (we use $|t| > 2$), and the subclass contains a sufficient number of treated and control units, it is split at the median propensity score. This recursive splitting continues until each resulting subclass satisfies two constraints: (i) it contains at least five treated and five control observations, and (ii) the total subclass sample size is at least equal to the number of covariates plus two. This process ensures sufficient overlap in propensity scores within each subclass to support valid causal inference.
3. **Ensure covariate balance in each subclass.** After subclassification, we perform quality control (QC) on covariate balance within each subclass. For each covariate in each subclass, we compute

a t-statistic comparing the means between treated and control observations. To assess overall balance across the project, we test whether the distribution of all subclass-level t-statistics (across all covariates and subclasses) deviates significantly from a standard Gaussian distribution. We apply a Bonferroni correction for multiple projects, using a significance level of $\alpha_{QQ} = 5\%/44 = 0.11\%$. We iteratively remove the subclass with the most extreme t-statistic falling outside the $(1 - \alpha_{QQ})$ confidence bands of a Gaussian QQ-plot [Weine et al., 2023], and repeat the test until the remaining t-statistics are consistent with Gaussianity. This procedure reduces the risk of including subclasses with systematic covariate imbalance between treated and control units, as recommended in Section 13.6 of Imbens and Rubin [2015].

For each REDD+ project, the process above produces four matched datasets per project, each varying in the percentage of treated plots successfully matched. This percentage varies depending on the project, the caliper width used for matching, and the quality control filter applied. We choose the dataset of the four with the highest percentage of matched treated plots for each project to take forward to Average Treatment Effect on the Treated (ATT) estimation.

5 Estimating causal impact of projects on deforestation (the ATT)

We fit and compare a suite of causal models to estimate the ATT and assess the robustness of our findings to alternative modeling assumptions. All models rely on the assumption of selection on observables but differ in their statistical specifications, including how outcomes are modeled and how covariate adjustment is handled. By comparing ATT estimates across models that use propensity score subclassification, regression adjustment, matching alone, inverse-probability weighting, and spatial error structures, we assess sensitivity to different forms of residual confounding, non-standard outcome distributions, and spatial autocorrelation. This model diversity helps identify cases where ATT estimates are stable and where they are sensitive to specific assumptions—providing a fuller picture of the credibility of our causal claims. Full technical details are provided below.

A. Linear model with propensity score sub-classification. For the plots in subclass s of project i we fit the following model via ordinary least squares, which has different means for control ($d_{i,j} = 0$) and treated ($d_{i,j} = 1$) plots:

$$y_{i,j} = \begin{cases} \mu_{i,0}^{(s)} + \varepsilon_{i,j} & d_{i,j} = 0 \\ \mu_{i,0}^{(s)} + \tau_i^{(s)} + \varepsilon_{i,j} & d_{i,j} = 1 \end{cases} \qquad \text{for } j \in \mathcal{J}_i^{(s)}, \tag{2}$$

where $\mu_{i,0}^{(s)}$ is the mean outcome in a control plot, $\tau_i^{(s)}$ is the average difference between control and treated plots (the ATT), and $\varepsilon_{i,j}$ are independent error terms with constant variance $\mathbb{V}(\varepsilon_{i,j}) = \sigma_i^2$. We can write (2) more concisely as

$$y_{i,j} = \mu_{i,0}^{(s)} + \tau_i^{(s)} \ \mathbb{I}(d_{i,j} = 1) + \varepsilon_{i,j} \tag{3}$$

where the indicator function $\mathbb{I}(A) = 1$ if statement A is true, and $\mathbb{I}(A) = 0$ if A is false. The ATT estimate in the sth subclass is

$$\widehat{\text{ATT}}_i^{(s)} := \hat{\tau}_i^{(s)} \tag{4}$$

The overall ATT for project i is then estimated as the average of these within-subclass estimates, weighted by subclass sample size [Imbens and Rubin, 2015]:

$$\widehat{\text{ATT}}_i := \sum_{s=1}^{S_i} \frac{|\mathcal{J}_i^{(s)}|}{J_i} \widehat{\text{ATT}}_i^{(s)} \tag{5}$$

$$\widehat{\mathbb{V}}\left(\widehat{\text{ATT}}_i\right) := \sum_{s=1}^{S_i} \frac{|\mathcal{J}_i^{(s)}|^2}{J_i^2} \widehat{\mathbb{V}}\left(\widehat{\text{ATT}}_i^{(s)}\right) , \tag{6}$$

where $\mathcal{J}_i^{(s)}$ denotes the set of plots in subclass s of project i.

B. Doubly robust linear model with propensity score sub-classification. For the plots in subclass s of project i we refine model (3) to include a linear predictor of measured potential confounders:

$$y_{i,j} = \mu_{i,0}^{(s)} + \tau_i^{(s)} \ \mathbb{I}(d_{i,j} = 1) + \boldsymbol{\beta}^T \boldsymbol{x}_{i,j} + \varepsilon_{i,j} \qquad \text{for } j \in \mathcal{J}_i^{(s)} \tag{7}$$

The overall ATT for project i is then estimated by combining the $\hat{\tau}_i^{(s)}$ across subclasses as in (4)-(6).

C. Categorical/quantitative statistical model with propensity score sub-classification. Here we define an alternative statistical model for deforestation proportion outcome $y_{i,j}$:

$$p(\boldsymbol{c}_i, \boldsymbol{d}_i) = \prod_{j=1}^{J_i} p(c_{i,j} \mid d_{i,j}) p(y_{i,j} \mid c_{i,j}, d_{i,j}) \tag{8}$$

with conditional mean parameters $\pi_{i,d}$ and $\mu_{i,d}$ such that, for $j = 1, \ldots, J_i$,

$$\mathbb{P}(c_{i,j} = 1 \mid d_{i,j} = d) = \pi_{i,d} \tag{9}$$

$$\mathbb{E}[y_{i,j} \mid c_{i,j}, d_{i,j} = d] = \begin{cases} \mu_{i,d} & c_{i,j} = 1 \\ 0 & c_{i,j} = 0 \end{cases} \tag{10}$$

(recall that $c_{i,j} = 1 \Leftrightarrow y_{i,j} > 0$). This model explicitly incorporates the property that the outcome distribution is bounded below by zero. We estimate the conditional expectations of $c_{i,j}$ and $y_{i,j}$ within each propensity score subclass $s = 1, \ldots, S_i$:

$$\hat{\pi}_{i,d}^{(s)} := \frac{\sum_{j \in \mathcal{J}_i^{(s)}} c_{i,j} \mathbb{I}(d_{i,j} = d)}{\sum_{j \in \mathcal{J}_i^{(s)}} \mathbb{I}(d_{i,j} = d)} \tag{11}$$

$$\widehat{\mathbb{V}}\left(\hat{\pi}_{i,d}^{(s)}\right) := \frac{\hat{\pi}_{i,d}^{(s)}(1 - \hat{\pi}_{i,d}^{(s)})}{\sum_{j \in \mathcal{J}_i^{(s)}} \mathbb{I}(d_{i,j} = d)} \tag{12}$$

$$\hat{\mu}_{i,d}^{(s)} := \frac{\sum_{j \in \mathcal{J}_i^{(s)}} y_{i,j} \mathbb{I}(d_{i,j} = d \wedge c_{i,j} = 1)}{\sum_{j \in \mathcal{J}_i^{(s)}} \mathbb{I}(d_{i,j} = d \wedge c_{i,j} = 1)} \tag{13}$$

$$\widehat{\mathbb{V}}\left(\hat{\mu}_{i,d}^{(s)}\right) := \frac{\texttt{SampleVariance}(\{y_{i,j} : j \in \mathcal{J}_i^{(s)},\ d_{i,j} = d \wedge c_{i,j} = 1\})}{\sum_{j \in \mathcal{J}_i^{(s)}} \mathbb{I}(d_{i,j} = d \wedge c_{i,j} = 1)} \tag{14}$$

where $\mathcal{J}_i^{(s)}$ is the set of plots in the sth subclass in project i. The estimated ATT in the sth subclass is:

$$\widehat{\text{ATT}}_i^{(s)} := \hat{\pi}_{i,1}^{(s)} \hat{\mu}_{i,1}^{(s)} - \hat{\pi}_{i,0}^{(s)} \hat{\mu}_{i,0}^{(s)} \tag{15}$$

with the ATT estimator's sampling variance estimated following Goodman [1960]:

$$\widehat{\mathbb{V}}\left(\widehat{\text{ATT}}_i^{(s)}\right) := \sum_{d=0}^{1} \left(\widehat{\mathbb{V}}\left(\hat{\mu}_{i,d}^{(s)}\right) + \left(\hat{\mu}_{i,d}^{(s)}\right)^2\right)\left(\widehat{\mathbb{V}}\left(\hat{\pi}_{i,d}^{(s)}\right) + \left(\hat{\pi}_{i,d}^{(s)}\right)^2\right) - \left(\hat{\mu}_{i,d}^{(s)}\right)^2 \left(\hat{\pi}_{i,d}^{(s)}\right)^2 . \tag{16}$$

The overall ATT for project i is then estimated by combining the $\hat{\tau}_i^{(s)}$ across subclasses as in (5) and (6).

D. Spatially correlated linear model with propensity score sub-classification. For the plots in subclass s of project i we fit a version of model (3) with spatially correlated residuals having an isotropic Gaussian semivariogram [Pinheiro and Bates, 2009, Cressie, 2015],

$$\gamma(\Delta(\varepsilon_{i,j}, \varepsilon_{i,j'}), \lambda) := 1 - \exp\left(-\frac{\Delta(\varepsilon_{i,j}, \varepsilon_{i,j'})^2}{\lambda^2}\right), \tag{17}$$

where $\Delta(\varepsilon_{i,j}, \varepsilon_{i,j'})$ is the Euclidean distance between plots j and j' in project i. The overall ATT for project i is then estimated by combining the $\hat{\tau}_i^{(s)}$ across subclasses as in (4)-(6).

E. Simple linear model fitted to matched data. For all plots in project i's matched dataset we fit the following model via least squares:

$$y_{i,j} = \mu_{i,0} + \tau_i \ \mathbb{I}(d_{i,j} = 1) + \varepsilon_{i,j} \tag{18}$$

where $\mathbb{V}(\varepsilon_{i,j}) = \sigma_i^2$. The ATT estimate for project i is then $\hat{\tau}_i$.

F. Adjusted linear model fitted to matched data. For all plots in project i's matched dataset we fit the following model via least squares:

$$y_{i,j} = \mu_{i,0} + \tau_i \ \mathbb{I}(d_{i,j} = 1) + \boldsymbol{\beta}^T \boldsymbol{x}_{i,j} + \varepsilon_{i,j} \tag{19}$$

where $\mathbb{V}(\varepsilon_{i,j}) = \sigma_i^2$. The ATT estimate for project i is then $\hat{\tau}_i$.

G. Linear model with propensity score weighting. For all plots in project i's matched dataset we fit the following model via weighted least squares:

$$y_{i,j} = \mu_{i,0} + \tau_i \; \mathbb{I}(d_{i,j} = 1) + \varepsilon_{i,j} \tag{20}$$

$$\mathbb{V}(\varepsilon_{i,j}) = \begin{cases} e_{i,j} \; \sigma_i^2 & d_{i,j} = 1 \\ (1 - e_{i,j}) \; \sigma_i^2 & d_{i,j} = 0 \end{cases} \tag{21}$$

where $e_{i,j}$ is the propensity score for plot j in project i. The ATT estimate for project i is then $\hat{\tau}_i$.

H. Panel model fitted to matched data with fixed effects for entities and time.

For each project i, let $\mathcal{J}_i$ be the set of matched plots, and let t index *relative* year with $t = 0$ the treatment year. We restrict to $t \geq -5$. Define $D_{ij} = \mathbb{I}(\text{treat}_{ij} = 1)$ and $\text{Post}_{it} = \mathbb{I}(t > 0)$. We estimate, by OLS with high-dimensional fixed effects,

$$z_{ijt} = a_j \; + \; b_t \; + \; \tau_i \left(D_{ij} \times \text{Post}_{it}\right) \; + \; \varepsilon_{ijt}, \qquad j \in \mathcal{J}_i, \; t \in \{-5, \dots, 5\} \tag{22}$$

where a_j (plot) and b_t (relative-time) are fixed effects, and $\mathbb{V}(\varepsilon_{ijt})$ is left unspecified (heteroskedasticity and arbitrary within-cluster dependence allowed). The project-level ATT is $\hat{\tau}_i$, i.e., the coefficient on $D_{ij} \times \text{Post}_{it}$.

Implementation. We fit (22) with `fixest::feols` using two-way clustering by plot and relative-time and finite-sample corrections (`ssc(cluster.adj=TRUE, fixef.K="nested")`). Inference uses the cluster-robust variance estimator and Wald intervals for τ_i. If all outcomes are zero in a project ($z_{ijt} \equiv 0$), we define $\hat{\tau}_i = 0$ with $\text{se}(\hat{\tau}_i) = 0$ (model not estimable). Outcomes $z_{ijt} \in [0, 100]$ denote annual % forest loss in plot j of project i during year t, measured relative to estimated forest cover at year -6.

I. Panel model fitted to matched data fixed effects for entities and time and an interaction term between the time and the sub-national geographic units.

For each project i, let $\mathcal{J}_i$ be the set of matched plots, and let t index *relative* year with $t = 0$ the treatment year. We restrict to $t \geq -5$. Define $D_{ij} = \mathbb{I}(\text{treat}_{ij} = 1)$ and $\text{Post}_{it} = \mathbb{I}(t > 0)$. We estimate, by OLS with high-dimensional fixed effects,

$$z_{ijt} = a_j \; + \; b_t \; + \; c_{g(j) \times t} \; + \; \tau_i \left(D_{ij} \times \text{Post}_{it}\right) \; + \; \varepsilon_{ijt}, \qquad j \in \mathcal{J}_i, \; t \in \{-5, \dots, 5\} \tag{23}$$

where a_j (plot) and b_t (relative-time) are fixed effects, and $c_{g(j) \times t}$ denotes an interaction between relative time and the ADM2 sub-national geographic unit $g(j)$ in which plot j is nested. $\mathbb{V}(\varepsilon_{ijt})$ is left unspecified (heteroskedasticity and arbitrary within-cluster dependence allowed). The project-level ATT is $\hat{\tau}_i$, i.e., the coefficient on $D_{ij} \times \text{Post}_{it}$.

Implementation. We fit (23) with `fixest::feols` using two-way clustering by plot and relative-time and finite-sample corrections (`ssc(cluster.adj=TRUE, fixef.K="nested")`). If all outcomes are zero in a project ($z_{ijt} \equiv 0$), we define $\hat{\tau}_i = 0$ with $\text{se}(\hat{\tau}_i) = 0$ (model not estimable). Outcomes $z_{ijt} \in [0, 100]$ denote annual % forest loss in plot j of project i during year t, measured relative to estimated forest cover at year -6. Sub-national geographic units (ADM2 level) were obtained from the geoBoundaries database [Runfola et al., 2020].

Figure 4 in the main text shows the ATT results for the 36 projects meeting full QC criteria. ATT estimates for the 44 matched projects, including those with a matched treated sample size $< 80\%$, are provided in Figures S4 and S8.

6 Evaluating whether unobserved confounders explain differences between our ATT estimates and avoided deforestation claimed by the projects (sensitivity analysis)

Official estimates of avoided deforestation, derived from Verified Carbon Standard (VCS) project monitoring reports and aggregated over the first five years, generally exceed our ATT estimates of deforestation

averted. This discrepancy may result from hidden confounders influencing our ATT estimates. To explore this possibility, we assess whether hidden confounders, with strengths comparable to those of our datasets' measured confounders, could explain the differences between our estimates and the official values. Specifically, we estimate the strength of measured confounders in unmatched data and adjust our ATT estimates for hypothetical hidden confounders at one, two, and three times (1x, 2x, 3x) the strength of the measured confounders.

The methods developed by [Cinelli and Hazlett, 2019] were designed specifically for this purpose. Within their framework, we introduce a hypothetical hidden confounder, z, into our adjusted linear model (19) fitted to matched data:

$$y_j = \mu_0 + \tau \mathbb{I}(d_j = 1) + \boldsymbol{\beta}^T \boldsymbol{x}_j + \gamma z_j + \varepsilon_j \ , \tag{24}$$

where $\mathbb{V}(\varepsilon_{i,j}) = \sigma^2$, and for clarity we have dropped dependence on project i in this section.

Within the framework of [Cinelli and Hazlett, 2019], we can examine how adjustment for z could change our ATT estimate $\hat{\tau}$, and thereby how it might bring $\hat{\tau}$ into closer agreement with the official estimate, denoted v. [Cinelli and Hazlett, 2019] derive relationships between the statistical properties of hidden confounder z, and bounds on $\hat{\tau}$. The two statistical properties inputted are (i) the partial R^2 between hidden z and treatment d, conditional on observed $\boldsymbol{x}$, denoted $R^2_{d\sim z|\boldsymbol{x}}$; and (ii) the partial R^2 between hidden z and outcome y, conditional on $\boldsymbol{x}$ and d, denoted $R^2_{y\sim z|d,\boldsymbol{x}}$. Figure S2shows the estimated partial R^2 for each of the measured confounders in this study (distance to degradation, slope, elevation and accessibility).

[Cinelli and Hazlett, 2019] provide methods to calculate $R^2_{d\sim z|\boldsymbol{x}}$ and $R^2_{y\sim z|d,\boldsymbol{x}}$ so that z has the same (or a multiple of the) strength of confounding as a measured confounder x_k, with that strength being quantified similarly through partial R^2 statistics conditional on the other observed confounders $\boldsymbol{x}_{-k}$, i.e. through $R^2_{d\sim x_k|\boldsymbol{x}_{-k}}$ and $R^2_{y\sim x_k|d,\boldsymbol{x}_{-k}}$. Our process for sensitivity analysis for hidden confounder z that has m times (we try values $m = 1, 2, 3$) the strength of confounding of measured variable x_k is performed using `sensemakr` R software [Cinelli and Hazlett, 2019, R Core Team, 2021] as follows:

1. Estimate $R^2_{d\sim x_k|\boldsymbol{x}_{-k}}$ and $R^2_{y\sim x_k|d,\boldsymbol{x}_{-k}}$ in *unmatched* data;
2. Calculate $R^2_{d\sim z|\boldsymbol{x}}$ and $R^2_{y\sim z|d,\boldsymbol{x}}$ that correspond to m times the strength of confounding of x_k (we set $k_D = k_Y = m$ in equations (21)-(22) of [Cinelli and Hazlett, 2019]);
3. Compute estimate and credible interval for τ in matched data when additionally adjusted for hidden confounder z.

Technical details As m increases, we select the direction of change in $\hat{\tau}$ to make it closer to the official estimate v; in our datasets we observe $\hat{\tau} > v$ in all projects, i.e. less avoided deforestation than suggested by official estimates; so, increasing m reduces the adjusted estimate $\hat{\tau}$. As $m \in \mathbb{R}_0^+$ increases, there is a finite value (denote it m_{asympt}) such that as $m \to m_{\text{asympt}}$ we have $R^2_{d\sim z|\boldsymbol{x}} \to 1$ and the adjusted value of $\hat{\tau} \to -\infty$. In cases where $m > m_{\text{asympt}}$ we present adjusted $\hat{\tau}$ as being less than a large negative threshold (e.g. as $\hat{\tau} < -8$ in Figure S5). In order to perform the analysis in the `sensemakr` framework, we used the official value v as an offset in the linear model at (24):

$$y_j = \mu_0 + (v + \theta)\mathbb{I}(d_j = 1) + \boldsymbol{\beta}^T \boldsymbol{x}_j + \gamma z_j + \varepsilon_j \ , \tag{25}$$

so that θ represents the difference between our ATT τ and official estimate v. This allows us to examine the adjustment for hidden confounder in how it decreases θ towards and beyond zero (our estimate agrees with the official estimate at $\theta = 0$).

Figure 5 of the main text shows the sensitivity analysis results for 23 out of 36 projects for which we have comparable data on certified avoided deforestation. Sensitivity analyses for 26 out of the 44 matched projects, including those with a matched treated sample size less than 80%, are provided in Figure S5.

Alternative sensitivity analysis methods considered Panel-based sensitivity methods such as Keele et al. [2019] require explicit assumptions about how unobserved confounding evolves over time and quantify bias using a sensitivity parameter Γ, representing the maximum factor by which an unobserved confounder could differentially influence treatment assignment between periods. Although valuable for two-period or difference-in-differences designs, Γ cannot be readily interpreted in terms of ecological

quantities like variance explained or landscape attributes. Applying these methods would also require restricting identification entirely to within-plot temporal variation, discarding all time-invariant information; in our data, this produces substantially noisier and often smaller estimates, providing an unstable basis for benchmarking unobserved confounding. Oster-type bounds Oster [2019] require specifying $R_{\max}$ (the explanatory power of a hypothetical full model with all confounders) and assuming 'proportional selection', meaning that unobserved confounders affect treatment assignment in the same proportion as observed confounders. As noted by Cinelli and Hazlett [2019], this proportional-selection parameter is difficult to interpret and can behave counterintuitively. Given limited pre-treatment data and covariates that cannot plausibly capture most of the variation in deforestation pressure, these assumptions cannot be defended in our ecological setting. The E-value approach VanderWeele and Ding [2017] is assumption-light and works with summary statistics, but is fundamentally defined for risk ratios and most interpretable for binary outcomes. Extensions to continuous outcomes yield quantities without a natural ecological interpretation and cannot be benchmarked against observed covariates, in contrast to partial R^2–based sensitivity metrics.

Supplementary Figures

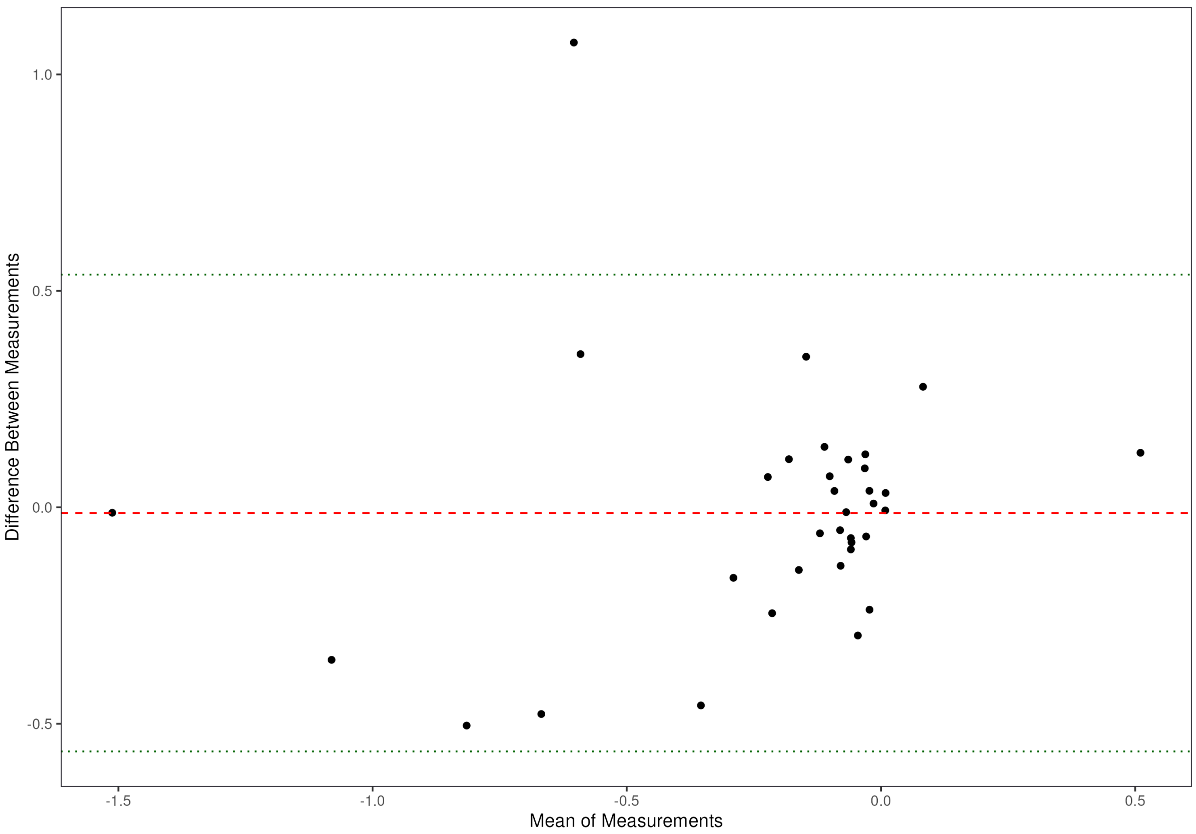

Figure S1: Bland-Altman plot comparing Guizar-Coutiño et al. [2022] and estimates provided in this study, using doubly robust models. The mean of the two methods for each examined site (x-axis) plotted against the difference between these estimates (y-axis). The red dashed line represents the mean difference in estimates across studies, while the horizontal dotted lines indicate the 95% limits of agreement (mean $\pm$ 1.96 $\times$ SD), ranging from –0.56 to 0.54.

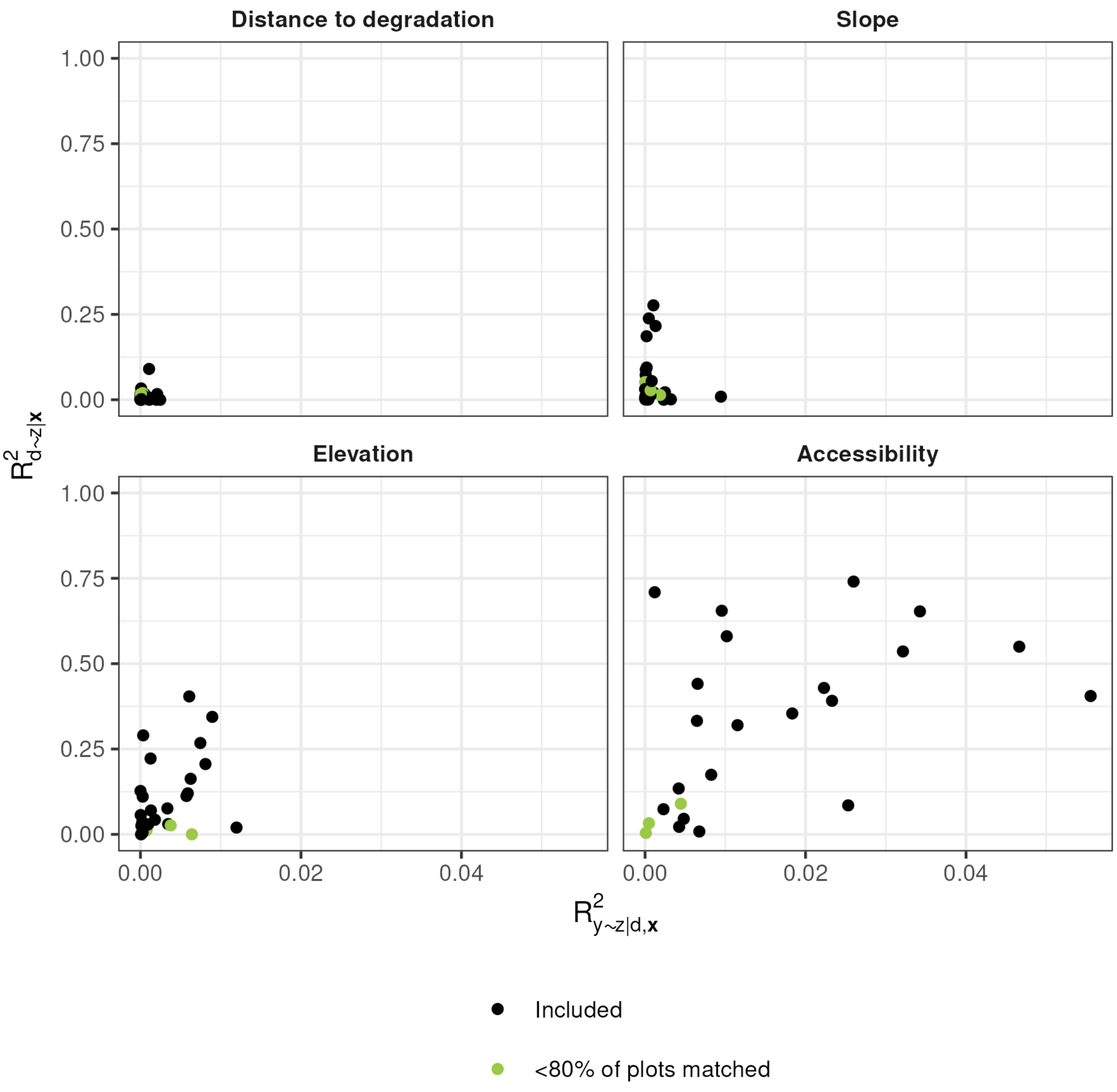

Figure S2: The estimated partial R^2 for each of the measured confounders (distance to degradation, slope, elevation and accessibility). Each point represents a project: those excluded from the main analysis because $<$ 80% of the treated plots were matched are shown in green. For each measured confounder z, the y-axis shows the proportion of the total variance in the treatment variable d (being included in a REDD+ project) explained by the confounder after accounting for the variance explained by the other covariates $\boldsymbol{x}$ (denoted $R^2_{d\sim z|\boldsymbol{x}}$). The x-axis shows partial R^2 between z and outcome y, (deforestation) conditional on $\boldsymbol{x}$ and d, denoted $R^2_{y\sim z|d,\boldsymbol{x}}$.

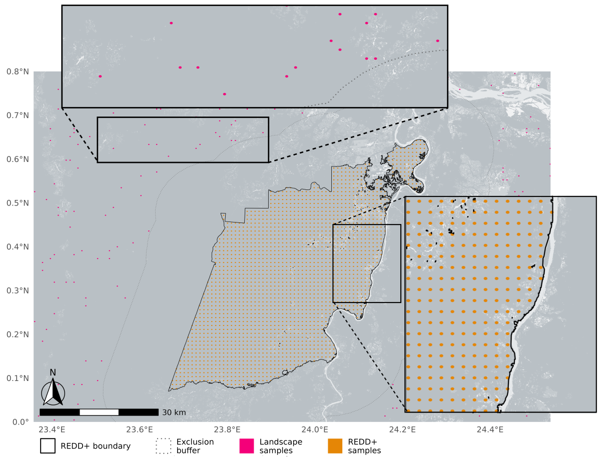

Figure S3: Illustration of the sampling strategy, for a REDD+ project in Democratic Republic of the Congo (VCS 1359 COD). Treatment plots were sampled from within the REDD+ project on a 1 km grid (orange), while potential control plots (pink) were randomly sampled from the wider landscape outside a 15 km buffer around the REDD+ project. The extent of undisturbed forest cover (dark grey; Vancutsem et al., 2021) was used as a reference for distributing sample plots across the moist tropics.

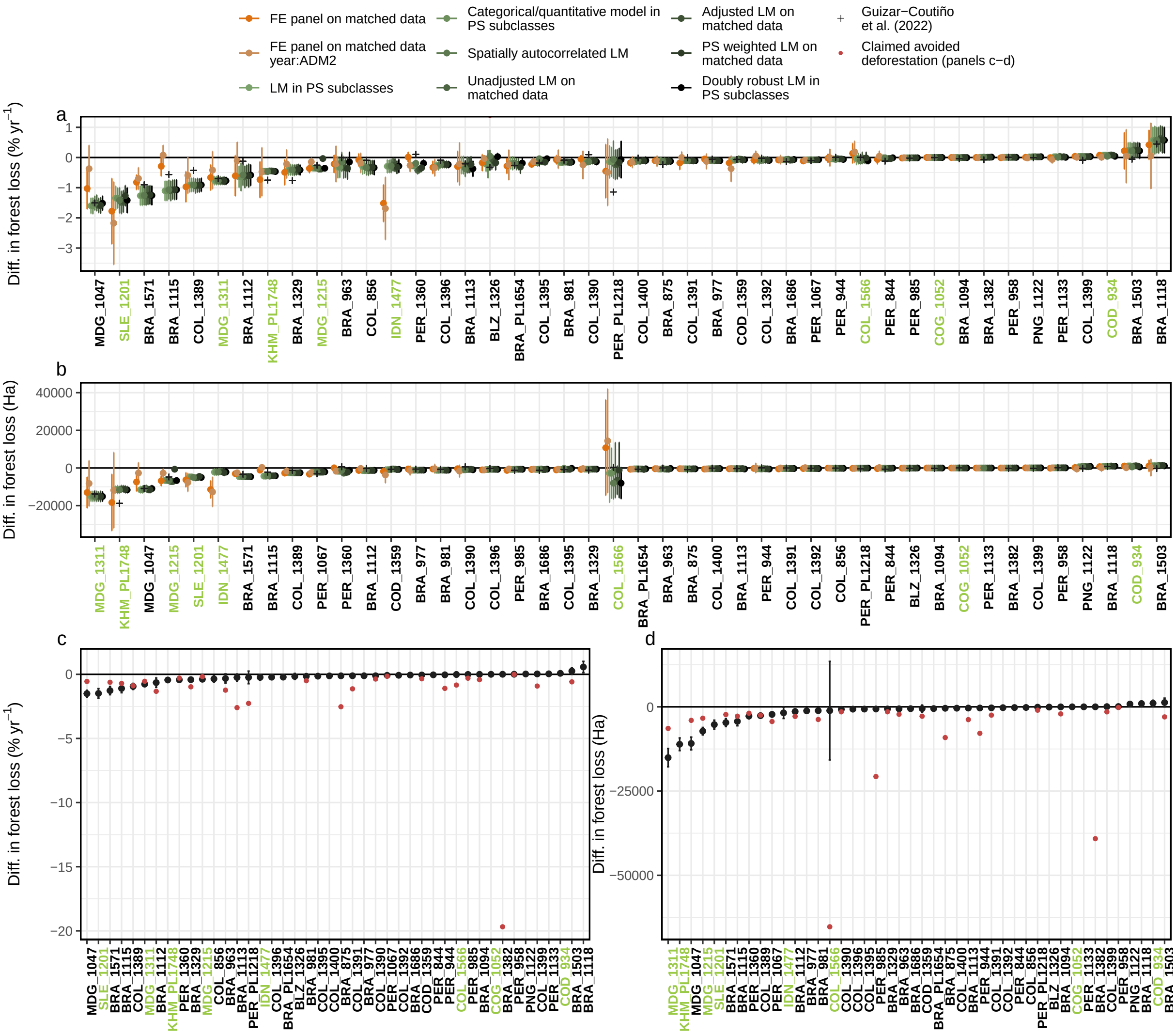

Figure S4: Average treatment effect on the treated (ATT) for the full set of 44 REDD+ projects. Estimates show differences in deforestation rates (**a**) and differences in the area of forest lost (**b**), for the for the full set of 44 REDD+ projects (those where < 80% of plots are matched are shown in green), across all model specifications. Error bars represent ±2 SEs. Estimates below the zero line indicate avoided deforestation, and the ATT from Guizar-Coutiño et al. [2022] is marked with a '+' symbol. The ATT estimated using the doubly robust linear model in propensity score subclass models is shown in deforestation rates (**c**) and area (**d**), compared with (in red) avoided deforestation claimed by projects in monitoring and evaluation reports in Swinfield et al. [2025]. Note this is shown only for the 26 projects for which we have data (again, projects labeled in green were not robustly matched).

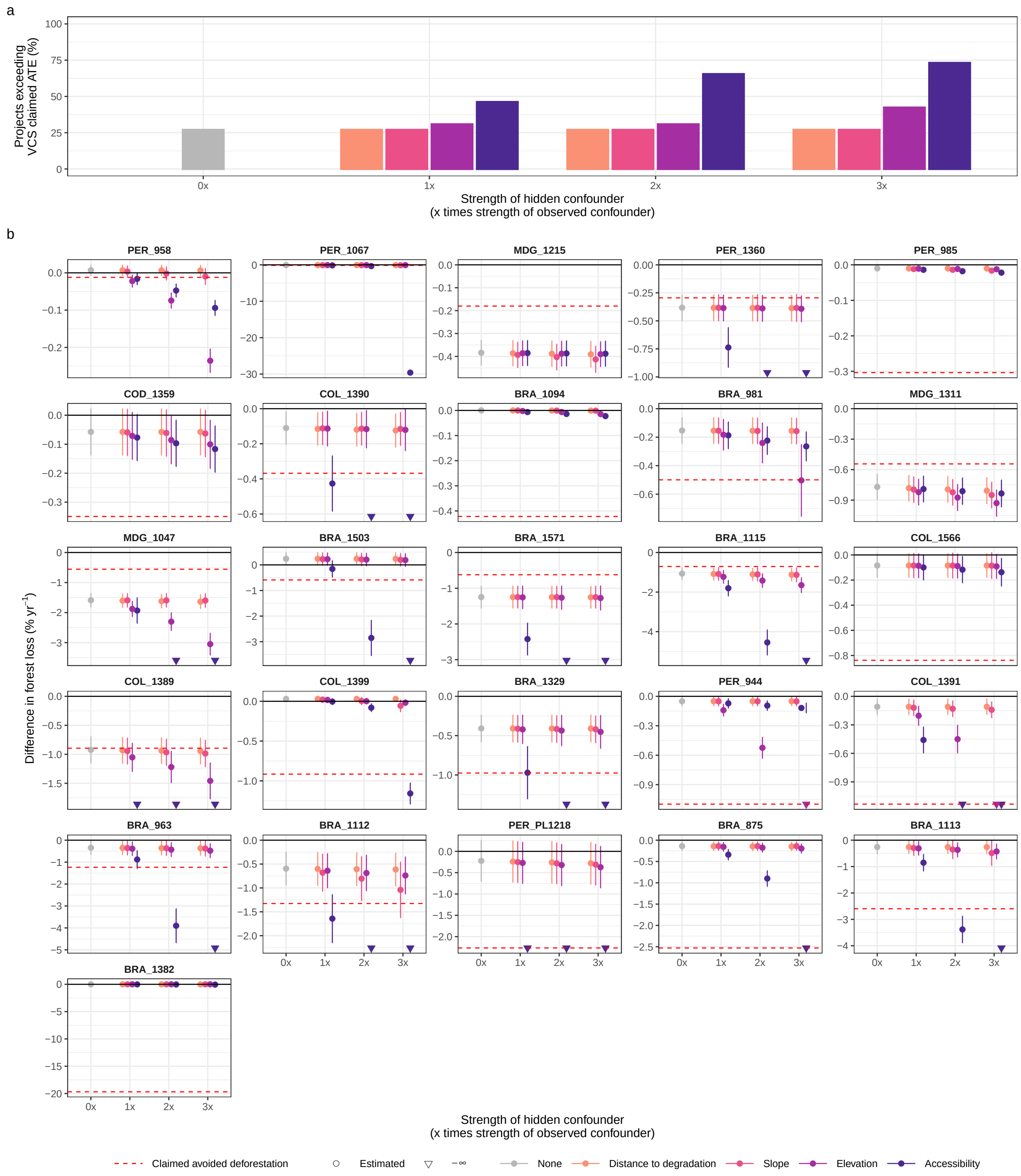

Figure S5: Sensitivity of estimates of avoided deforestation to unobserved confounders. Tests assessed the sensitivity of avoided deforestation estimates to unobserved confounders 1×, 2×, or 3× as strong as each measured confounder considered in this analysis. Results are shown for the 26 projects with available data on the avoided deforestation claimed by the project, including those where fewer than 80% of treated plots were successfully matched. (**a**) Percentage of projects that reached or exceeded the avoided deforestation claimed by the projects when ATTs are re-calculated considering unobserved confounders 1 to 3 times as strong as measured confounders. (**b**) Re-estimated ATT values under varying unobserved confounder strengths, with the red dashed line indicating the avoided deforestation claimed by each project and error bars representing ±2 SEs. ATT values with out-of-range estimates (−∞) are shown with triangles.

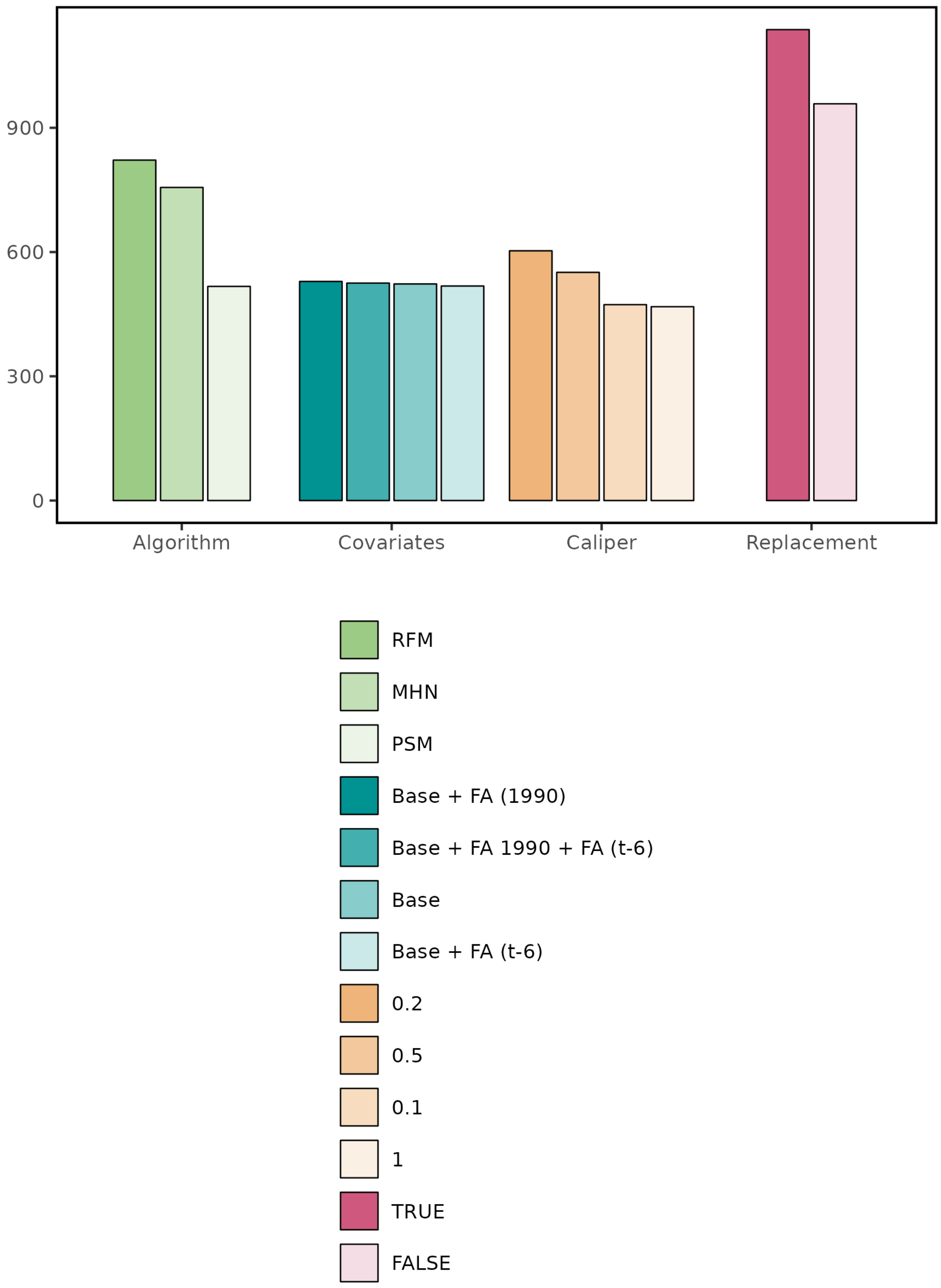

Figure S6: The number of matched sets that passed the quality control criteria, subdivided by specifications made when matching. A total of 96 distinct matching specifications were applied in each of 44 REDD+ sites. The specifications included: a) four choices of measured confounders combinations; base only (i.e. elevation, slope, accessibility, distance to degradation, short-term and long-term deforestation trends), base plus forest cover at the time of project implementation (at year −6), base plus forest cover in 1990, and base + forest cover in 1990 and implementation year (at year −6); b) choice of matching algorithm between Mahalanobis (MHN), propensity score (PSM), and Random Forest (RFM); c) choice of filtering regime (caliper sizes 0.1, 0.2, 0.5 and 1 SD); and d) choice of whether to allow matching with replacement or not.

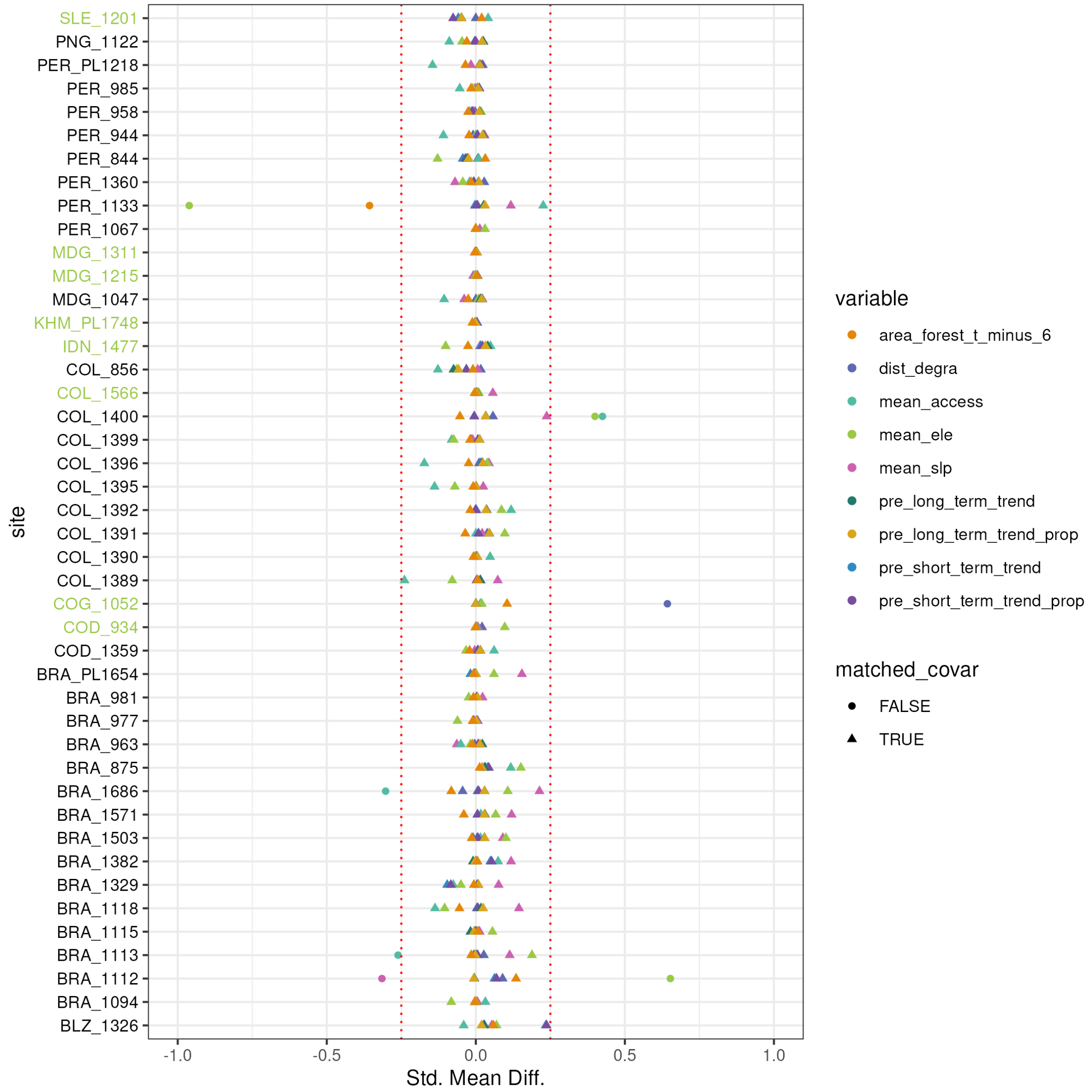

Figure S7: Post-matching standardized mean differences across covariates for selected matching models, from which post-matching analyses were developed. Projects labelled in green are the projects which were not robustly matched (i.e., we found suitable matches for less than 80% of treated plots).

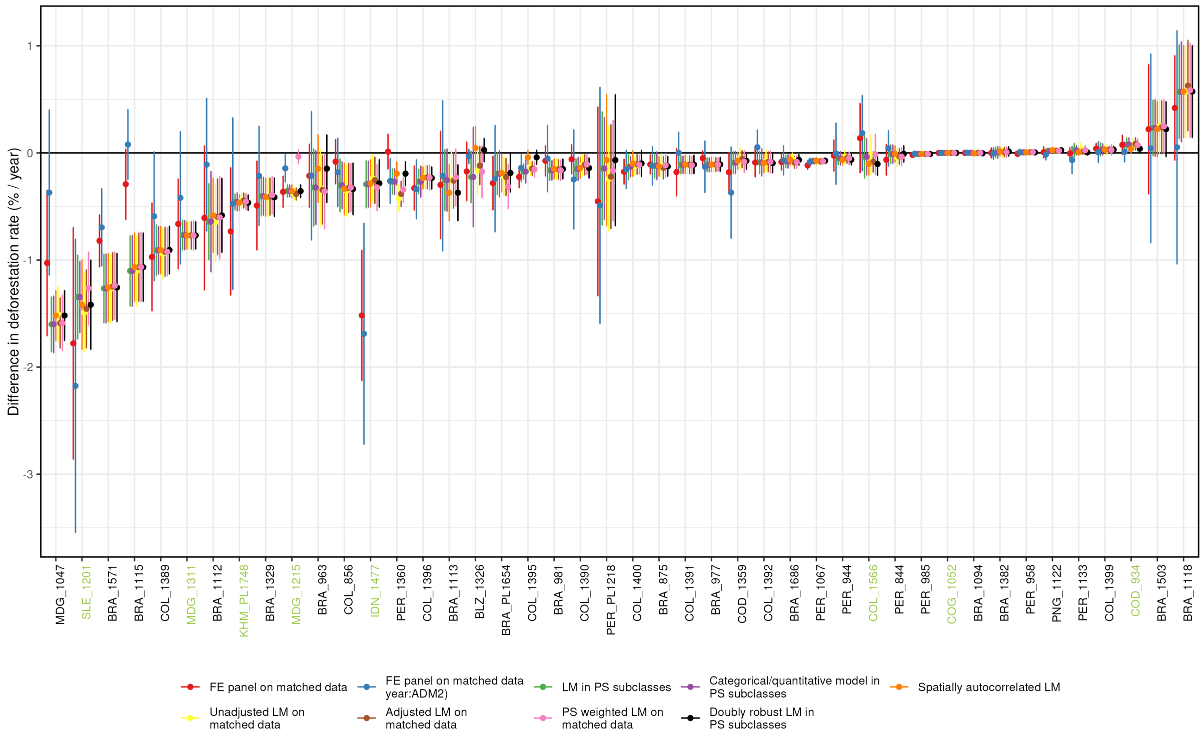

Figure S8: ATT by method. Projects labelled in green are the projects which were not robustly matched (i.e., we did not find suitable matches for less than 80% of treated plots).

References

Jonah Busch and Kalifi Ferretti-Gallon. What Drives and Stops Deforestation, Reforestation, and Forest Degradation? An Updated Meta-analysis. *Review of Environmental Economics and Policy*, pages 000–000, August 2023. ISSN 1750-6816.

Carlos Cinelli and Chad Hazlett. Making Sense of Sensitivity: Extending Omitted Variable Bias. *Journal of the Royal Statistical Society Series B: Statistical Methodology*, 82(1):39–67, December 2019. ISSN 1369-7412. doi: 10.1111/rssb.12348. URL https://doi.org/10.1111/rssb.12348. _eprint: https://academic.oup.com/jrsssb/article-pdf/82/1/39/49320681/jrsssb_82_1_39.pdf.

Noel Cressie. *Statistics for spatial data.* John Wiley & Sons, 2015.

Sébastien Desbureaux. Subjective modeling choices and the robustness of impact evaluations in conservation science. *Conservation Biology*, 35(5):1615–1626, 2021. ISSN 1523-1739.

Leo A. Goodman. On the Exact Variance of Products. *Journal of the American Statistical Association*, 55 (292):708–713, December 1960. ISSN 0162-1459. doi: 10.1080/01621459.1960.10483369. URL https://www.tandfonline.com/doi/abs/10.1080/01621459.1960.10483369. Publisher: Taylor & Francis _eprint: https://www.tandfonline.com/doi/pdf/10.1080/01621459.1960.10483369.

Alejandro Guizar-Coutiño, Julia P. G. Jones, Andrew Balmford, Rachel Carmenta, and David A. Coomes. A global evaluation of the effectiveness of voluntary REDD+ projects at reducing deforestation and degradation in the moist tropics. *Conservation Biology*, 36(6):e13970, 2022. ISSN 1523-1739. doi: 10.1111/cobi.13970. URL https://onlinelibrary.wiley.com/doi/abs/10.1111/cobi.13970.

Guido W. Imbens and Donald B. Rubin. *Causal Inference for Statistics, Social, and Biomedical Sciences: An Introduction.* Cambridge University Press, Cambridge, 2015. ISBN 978-0-521-88588-1. doi: 10.1017/CBO9781139025751. URL https://www.cambridge.org/core/books/causal-inference-for-statistics-social-and-biomedical-sciences/71126BE90C58F1A431FE9B2DD07938AB.

A Jarvis, H I Reuter, A Nelson, and E Guevara. Hole-filled SRTM for the Globe. V4. Technical report, CGIAR / CGIAR, 2008. URL http://srtm.csi.cgiar.org.

Luke J. Keele, Dylan S. Small, Jesse Y. Hsu, and Colin B. Fogarty. Patterns of Effects and Sensitivity Analysis for Differences-in-Differences. *arXiv*, 2019. doi: 10.48550/arXiv.1901.01869. URL http://arxiv.org/abs/1901.01869.

Emily Oster. Unobservable Selection and Coefficient Stability: Theory and Evidence. *Journal of Business & Economic Statistics*, 37(2):187–204, 2019. ISSN 0735-0015. doi: 10.1080/07350015.2016.1227711. URL https://doi.org/10.1080/07350015.2016.1227711.

José C. Pinheiro and Douglas Bates. *Mixed-Effects Models in S and S-PLUS.* Springer Science & Business Media, April 2009. ISBN 978-1-4419-0317-4. Google-Books-ID: y54QDUTmvDcC.

R Core Team. *R: A Language and Environment for Statistical Computing.* R Foundation for Statistical Computing, Vienna, Austria, 2021. URL https://www.R-project.org/.

Daniel Runfola, Austin Anderson, Heather Baier, Matt Crittenden, Elizabeth Dowker, Sydney Fuhrig, Seth Goodman, Grace Grimsley, Rachel Layko, Graham Melville, Maddy Mulder, Rachel Oberman, Joshua Panganiban, Andrew Peck, Leigh Seitz, Sylvia Shea, Hannah Slevin, Rebecca Youngerman, and Lauren Hobbs. geoBoundaries: A global database of political administrative boundaries. 2020.

Elizabeth A. Stuart. Matching methods for causal inference: A review and a look forward. *Statistical science : a review journal of the Institute of Mathematical Statistics*, 25(1):1–21, February 2010. ISSN 0883-4237. doi: 10.1214/09-STS313. URL https://www.ncbi.nlm.nih.gov/pmc/articles/PMC2943670/.

Tom Swinfield, Abby Williams, David Coomes, Michael Dales, Patrick Ferris, Alejandro Guizar-Coutiño, James Hartup, Jody Holland, Sadiq Jaffer, Julia Jones, Miranda Lam, Srinivasan Keshav, Anil Madhavapeddy, Eleanor Toye-Scott, Thales West, and Andrew Balmford. Learning lessons from over-crediting to ensure additionality in forest carbon credits. *Cambridge Open Engage*, 2025. doi: 10.33774/coe-2025-29fk2. URL https://www.cambridge.org/engage/coe/article-details/690c88f1ef936fb4a2b8d8de.

C. Vancutsem, F. Achard, J.-F. Pekel, G. Vieilledent, S. Carboni, D. Simonetti, J. Gallego, L. E. O. C. Aragão, and R. Nasi. Long-term (1990–2019) monitoring of forest cover changes in the humid tropics. 7(10):eabe1603, 2021. ISSN 2375-2548. URL https://advances.sciencemag.org/content/7/10/eabe1603.

Tyler J. VanderWeele and Peng Ding. Sensitivity Analysis in Observational Research: Introducing the E-Value. *Annals of Internal Medicine*, 167(4):268–274, 2017.

Eric Weine, Mary Sara McPeek, and Mark Abney. Application of Equal Local Levels to Improve Q-Q Plot Testing Bands with R Package qqconf. *Journal of Statistical Software*, 106:1–31, April 2023. ISSN 1548-7660. doi: 10.18637/jss.v106.i10. URL https://doi.org/10.18637/jss.v106.i10.

D J Weiss, A Nelson, H S Gibson, W Temperley, S Peedell, A Lieber, M Hancher, E Poyart, S Belchior, N Fullman, B Mappin, U Dalrymple, J Rozier, T C D Lucas, R E Howes, L S Tusting, S Y Kang, E Cameron, D Bisanzio, K E Battle, S Bhatt, and P W Gething. A global map of travel time to cities to assess inequalities in accessibility in 2015. 553(7688):333–336, 2018. ISSN 0028-0836, 1476-4687. URL http://dx.doi.org/10.1038/nature25181.

Table S1: The full list of REDD+ projects considered for this analysis (n=71), their area (ha), the reasons those excluded were excluded (colours follow those used in Figure 3), and whether they were included in the Guizar-Coutiño et al., (2022) analysis or not. The number of treated and potential control samples that were used in the matching runs are included in the sample size column. For the 44 projects taken forward to matching, project-level average treatment effect (ATT) estimated using the doubly robust linear model in propensity score subclasses are given, arranged by the % of treatment plots which were successfully matched. Projects where <80% of treatment plots were matched are highlighted in green (as in Figure 4). Projects are labeled with their Verified Carbon Standard code preceded by the International Organization for Standardization system: BLZ: Belize, BOL: Bolivia, BRA: Brazil, KHM: Cambodia, COL: Colombia, COG: Republic of Congo, COD: Democratic Republic of Congo, ETH: Ethiopia, GTM: Guatemala, IDN: Indonesia, KEN: Kenya, LAO: Laos, MDG: Madagascar, MWI: Malawi, MOZ: Mozambique, PNG: Papua New Guinea, PRY: Paraguay, PER: Peru, SLE: Sierra Leone, TZA: Tanzania, ZMB: Zambia, ZWE: Zimbabwe.

Project ID	Project Name	Area (km2)	Effect size [95% CI] (%/yr^{-1}). For projects where we could not estimate the effect size, reason for exclusion is give	Sample size<br>0= Untreated samples<br>1= Treated samples	% Matched	Y=Projects included in Guizar-Coutiño et al 2022, X=those not included	VCS Methodology (VM)
BLZ_852	Rio Bravo Climate Action Project	63.3	Insufficient temporal records	NA	NA	X	VM0007
BLZ_812	Bull Run Overseas Forest Carbon Project	6.76	<80% undisturbed	NA	NA	X	VM0007
BLZ_647	Boden Creek Ecological Preserve Forest Carbon Project	54.8	<80% undisturbed	NA	NA	X	VM0007
BLZ_1326	Laguna Seca Forest Carbon Project	87.2	0.03 [-0.08 0.14]	0= 7362, 1= 88	100	Y	VM0007
BOL_818	Protection of the Bolivian Amazon Forest	2.36	<80% undisturbed	NA	NA	X	VM0007
BRA_1686	Agrocortex REDD Project	1877	-0.06 [-0.12 -0.01]	0= 235120, 1= 1901	97	Y	VM0015
BRA_1113	The Valparaiso Project	292	-0.37 [-0.64 -0.11]	0= 233905, 1= 296	96	Y	VM0007
BRA_1112	The Russas Project	428	-0.58 [-0.93 -0.24]	0= 233905, 1= 427	95	Y	VM0007
BRA_1571	Manoa REDD+ Project	736	-1.26 [-1.58 -0.94]	0= 234794, 1= 751	99	Y	VM0015
BRA_1503	Resex Rio Preto-Jacundá REDD+ Project	1016	0.22 [-0.04 0.48]	0= 234143, 1= 1002	98	Y	VM0015

BRA_1382	The Envira Amazonia Project - A Tropical Forest Conservation Project in Acre, Brazil	397	0.01 [-0.03 0.06]	0= 234143, 1= 393	96	Y	VM0007
BRA_1329	Maísa REDD+ Project	302	-0.41 [-0.59 -0.23]	0= 234143, 1= 295	96	Y	VM0015
BRA_1118	Suruí Forest Carbon Project	336	0.57 [0.14 1]	0= 234633, 1= 328	96	Y	VM0015
BRA_1115	JARI/AMAPÁ REDD+ PROJECT	782	-1.07 [-1.39 -0.75]	0= 233905, 1= 740	97	Y	VM0015
BRA_1094	Ecomapua Amazon REDD Project	991	0 [-0.01 0]	0= 239030, 1= 991	82	Y	VM0015
BRA_981	ADPML PORTEL-PARA REDD PROJECT	1502	-0.15 [-0.24 -0.06]	0= 234974, 1= 1492	95	Y	VM0015
BRA_977	RMDLT PORTEL-PARA REDD PROJECT	2111	-0.11 [-0.17 -0.04]	0= 234974, 1= 2107	98	Y	VM0015
BRA_963	The Purus Project	357	-0.15 [-0.46 0.17]	0= 233905, 1= 353	96	Y	VM0007
BRA_875	FLORESTAL SANTA MARIA PROJECT	721	-0.13 [-0.22 -0.03]	0= 234633, 1= 734	99	Y	VM0007
BRA_PL1654	Fortaleza Ituxi REDD Project	473	-0.19 [-0.36 -0.01]	0= 234794, 1= 478	97	Y	VM0015
BRA_832	Cikel Brazilian Amazon REDD APD Project Avoiding Planned Deforestation	299	<80% undisturbed	NA	NA	X	VM0007
KHM_904	Reduced Emissions from Deforestation and Degradation in Community Forests – Oddar Meanchey, Cambodia	632	<80% undisturbed	NA	NA	X	VM0006
KHM_PL1689	Tumring REDD+ Project	683	Insufficient temporal records	NA	NA	X	VM0009

KHM_1650	Reduced Emissions from Deforestation and Degradation in Keo Seima Wildlife Sanctuary	1733	<80% undisturbed	NA	NA	X	VM0015
KHM_PL1748	Southern Cardamoms REDD+ Project	5005	-0.46 [-0.54 -0.39]	0= 11744, 1= 4797	39	Y	VM0009
COL_1566	REDD+ Project Resguardo Indigena Unificado Selva de Mataven (RIU SM) (started 2013)	15572	-0.1 [-0.21 0]	0= 213067, 1= 14108	1.2	Y	VM0007
COL_PL1695	Scheme of Compensation for Ecosystem Services for Forest Management and Conservation of Water Sources in the Jurisdiction of CORPOCHIVOR	24.6	<80% undisturbed	NA	NA	X	VM0015
COL_1400	Concosta REDD+ Project	650	-0.1 [-0.22 0.02]	0= 203672, 1= 659	97	Y	VM0006
COL_1392	Cajambre REDD+ Project	658	-0.09 [-0.19 0]	0= 203672, 1= 650	96	Y	VM0006
COL_1390	Carmen del Darién (CDD) REDD+ Project	1342	-0.14 [-0.24 -0.04]	0= 204533, 1= 1326	92	Y	VM0006
COL_1389	Acapa -- Bajo Mira Y Frontera REDD+ Project	554	-0.91 [-1.13 -0.68]	0= 203672, 1= 536	97	Y	VM0006
COL_1395	Bajo Calima y Bahía Málaga (BCBM) REDD+ Project	920	-0.04 [-0.11 0.03]	0= 203672, 1= 915	93	Y	VM0006
COL_1399	Mutatá REDD+ Project	405	0.03 [0 0.06]	0= 203505, 1= 411	99	Y	VM0006
COL_1396	Rio Pepe y ACABA REDD+ Project	611	-0.23 [-0.34 -0.13]	0= 204533, 1= 608	98	Y	VM0006
COL_1391	SUPP REDD+ Project	542	-0.11 [-0.19 -0.02]	0= 203672, 1= 540	99	Y	VM0006

COL_856	The Chocó-Darién Conservation Corridor REDD Project	107	-0.34 [-0.58 -0.09]	0= 204121, 1= 108	96	Y	VM0009
COL_PL1657	Grouped Project REDD+ conservation of the Oak ecological corridor Guantiva – La Rusia – Iguaque, Colombia	71.7	Insufficient temporal records	NA	NA	X	VM0015
COG_1052	North Pikounda REDD+	927	0 [0 0]	0= 14142, 1= 922	11	Y	VM0011
COD_1359	Isangi REDD+ Project	1994	-0.07 [-0.15 0.01]	0= 38961, 1= 1983	100	Y	VM0006
COD_934	The Mai Ndombe REDD+ Project	2796	0.04 [0 0.08]	0= 42073, 1= 2683	35	X	VM0009
ETH_1340	Bale Mountains Eco-region REDD+ project	2569	<80% undisturbed	NA	NA	X	VM0015
GTM_1541	Lacandon – Forest for life REDD+ Project	2050	<80% undisturbed	NA	NA	X	VM0015
GTM_1384	Reduced Emissions from Avoided Deforestation in the Multiple Use Zone of the Maya Biosphere Reserve in Guatemala (GuateCarbon)	7264	<80% undisturbed	NA	NA	X	VM0015
IDN_1477	Katingan Peatland Restoration and Conservation Project	1507	-0.28 [-0.51 -0.06]	0= 112761, 1= 2665	56	X	VM0007
IDN_674	Rimba Raya Biodiversity Reserve Project	476	<80% undisturbed	NA	NA	X	VM0004
KEN_1408	Chyulu Hills REDD+ Project	4162	<80% undisturbed	NA	NA	X	VM0009
KEN_612	The Kasigau Corridor REDD Project - Phase II The Community Ranches	1754	<80% undisturbed	NA	NA	X	VM0009
KEN_562	The Kasigau Corridor REDD Project – Phase I Rukinga Sanctuary	306	Insufficient temporal records	NA	NA	X	VM0009

LAO_1398	Reducing Emissions from Deforestation and Carbon Enhancement in Xe Pian National Protected Area	1429	<80% undisturbed	NA	NA	X	VM0015
MDG_1311	Carbon Emissions Reduction Project in the Corridor Ankeniheny-Zahamena (CAZ) Protected Area, Madagascar	3926	-0.77 [-0.9 -0.64]	0= 38077, 1= 4039	77	Y	VM0015
MDG_1215	The Makira Forest Protected Area in Madagascar	3747	-0.36 [-0.42 -0.3]	0= 41024, 1= 3847	73	Y	VM0007
MDG_1047	Carbon Emissions Reduction Project in the Forest Corridor Ambositra-Vondrozo (COFAV), Madagascar	1443	-1.52 [-1.75 -1.28]	0= 38771, 1= 1508	96	Y	VM0015
MDG_PL1743	Beampingaratsy REDD Project	660	Insufficient temporal records	NA	NA	X	VM0007
MWI_1168	Kulera Landscape REDD+ Program for Co-Managed Protected Areas, Malawi	2186	<80% undisturbed	NA	NA	X	VM0006
MOZ_PL1674	Gilé National Reserve REDD Project	1538	<80% undisturbed	NA	NA	X	VM0007
PNG_1122	April Salumei REDD Project	6539	0.03 [0.01 0.04]	0= 60504, 1= 6501	99	Y	VM0007
PRY_953	The Paraguay Forest Conservation Project - Reduction of GHG emissions from deforestation and forest degradation in the Chaco-Pantanal ecosystem	49	<80% undisturbed	NA	NA	X	VM0007

PER_958	BIOCORREDOR MARTIN SAGRADO REDD+ PROJECT	3003	0 [-0.01 0.02]	0= 295288, 1= 3015	95	Y	VM0015
PER_1360	Forest Management to reduce deforestation and degradation in Shipibo Conibo and Cacataibo Indigenous communities of Ucayali region	1293	-0.19 [-0.31 -0.08]	0= 295288, 1= 1298	99	Y	VM0015
PER_1133	Yacumama Forest Carbon Project	30.2	0.01 [-0.02 0.03]	0= 295288, 1= 29	93	X	VM0007
PER_1067	Reduction of deforestation and degradation in Tambopata National Reserve and Bahuaja-Sonene National Park within the area of Madre de Dios region – Peru	5658	-0.07 [-0.1 -0.04]	0= 295288, 1= 5760	90	Y	VM0007
PER_944	Alto Mayo Conservation Initiative	1787	-0.06 [-0.11 -0.01]	0= 295705, 1= 1722	98	Y	VM0015
PER_844	Madre de Dios Amazon REDD Project	985	-0.01 [-0.09 0.07]	0= 295272, 1= 996	97	Y	VM0007
PER_PL1455	Reduction of the deforestation and degradation of tropical dry forests in Piura and Lambayeque	398	<80% undisturbed	NA	NA	X	VM0015
PER_985	Cordillera Azul National Park REDD project	13627	-0.01 [-0.02 -0.01]	0= 295676, 1= 13747	97	Y	VM0007
PER_PL1218	Evio Kuiñaji Ese´Eja Cuana, To Mitigate Climate Change, Madre de Dios - Perú	87.2	-0.07 [-0.68 0.55]	0= 294578, 1= 88	99	Y	VM0007
SLE_1201	Gola REDD project	706	-1.42 [-1.84 -1]	0= 8447, 1= 719	48	X	VM0007

TZA_1325	Mjumita Community Forest Project (Lindi)	665	<80% undisturbed	NA	NA	X	VM0015
ZMB_1532	COMACO Landscape Management Project	14260	<80% undisturbed	NA	NA	X	VM0015; VM0017
ZMB_1202	LOWER ZAMBEZI REDD+ PROJECT	403	<80% undisturbed	NA	NA	X	VM0009
ZWE_902	KARIBA REDD+ PROJECT	10756	<80% undisturbed	NA	NA	X	VM0007

*For consistency with Guizar-Coutino et al. 2022, Project PL1748 in Cambodia is retained despite not meeting the time-frame criteria.

Table S2: Observed confounders used in matching, with rational for inclusion, source of data and format and resolution.

**Covariate**	**Rationale for inclusion**	**Source**	**Format and resolution**
Elevation (meters), Slope (degrees)	Elevation and slope influence land use practices, with higher elevation and steeper slopes consistently associated with lower risk of deforestation (Joppa a& Pfaff 2009.	SRTM Digital Elevation Data Version 4 (Jarvis et al. 2008).	Raster (90 m^2)
Accessibility (travel time to population centers in seconds)	Accessibility to forest lands from urban centres is associated with a higher risk of deforestation (Chomitz et al. 1999; Rudel et al. 2009).	Global accessibility map (Weiss et al. 2018).	Raster (300 m^2), data from 2015
Proximity to recent degradation events (Euclidean distance in meters)	Proximity to forest disturbances is consistently associated with higher deforestation risk (Busch & Ferretti-Gallon 2023). We computed the average distance to the closest degraded pixel, as characterized by the TMF's degradation forest class.	Own analysis using the JRC Tropical Moist Forests database (Vancutsem et al. 2021).	Raster (30 m^2)
Area of forest disturbances (Ha)	The risk of deforestation increases when there are cleared or disturbed patches of forest (Busch & Ferretti-Gallon 2023). We calculated the area of forest disturbances within plots by the year the project was started, by combining the extent of degraded and deforested forest as characterized by the TMF's degraded and deforested forest classes (class 2 and 3 respectively).	JRC Tropical Moist Forests database (Vancutsem et al. 2021).	Raster (30 m^2)
Undisturbed forest cover extent (Ha)	We used estimates of plot-level forest cover as characterized by the TMF's undisturbed forest class, taking measurements from 1990 (i.e. the beginning of the TMF records) to post-five years after project implementation.	JRC Tropical Moist Forest database (Vancutsem et al. 2021).	Raster (30 m^2)
Biome (Tropical moist forest)	Accounts for the biophysical characteristics of biomes, such as temperature, precipitation and agricultural suitability (Busch & Ferretti-Gallon 2023). Our sampling occurred in tropical moist forest regions for climatic comparability	JRC Tropical Moist Forest database (Vancutsem et al. 2021).	Raster (30 m^2)

Covariate	Rationale for inclusion	Source	Format and resolution
Country	Deforestation is influenced by domestic policies, governance, economic processes and markets (Umemiya et al. 2010; Ceddia et al. 2014).	Large Scale International Boundary (LSIB) dataset.	Vector data

Table S3: Covariate differences between included projects (n=36) and those excluded due to the unavailability of suitable matches for at least 80% of treated plots (n=8). Mean values for distance to populated areas (accessibility), distance to recent deforestation, slope, and elevation were derived using plot-level estimates. Long- and short-term deforestation trends indicate project-level proportional changes, assessed from 1990 to year 0 (implementation year) and from year -6 to year 0, respectively. Significant differences were tested using Wilcoxon rank-sum tests.

Covariate	Mean included (n=36)	Mean excluded (n=8)	Mean difference (W, p.value)
Mean plot level dist. to recent forest disturbances. (m)	970	1710	-739.71 (172, 0.41)
Mean plot level time travel to pop. centers (seconds)	767	879	-112 (156, 0.73)
Mean plot level elevation (m)	315	401	-85.97 (193, 0.14)
Mean plot level slope (degrees)	5.84	5.32	0.52 (123, 0.54)
Proj level long-term deforestation (prop.)	0.03	0.05	-0.02 (199, 0.1)
**Proj level short-term deforestation (prop.)**	**0.01**	**0.02**	**-0.01 (201, 0.09)**

Table S4: Project inclusion status and forest cover at the time of project implementation. Of the 71 projects for which we could obtain spatial data, five projects which operated for fewer than five years or commenced before 2000 were excluded from the analyses (BLZ_852, KHM_PL1689, COL_PL1657, KEN_562, MDG_PL1743; not shown below). Of the remaining 66, 22 contained < 80% of 'undisturbed' evergreen forest class as defined by the Tropical Moist Forest product (i.e. evergreen or semievergreen forest pixels which have not been disturbed over the full Landsat historical dataset), shown below as '<80% undisturbed forest cover'. The 44 REDD+ projects taken forward for subsequent analyses are indicated as 'Included for matching'. Columns Undisturbed, Degraded, Deforested, Regrowth, Water and Other refer to the TMF forest classes 1-6, respectively, and represent the composition of tropical forest classes within project boundaries at the time of project implementation. Projects are labelled with their Verified Carbon Standard code preceded by their country code using the International Organization for Standardization system: BLZ: Belize, BOL: Bolivia, BRA: Brazil, COD: Democratic Republic of the Congo, COG: Republic of the Congo, COL: Colombia, ETH: Ethiopia, GTM: Guatemala, IDN: Indonesia, KEN: Kenya, KHM: Cambodia, LAO: Laos, MDG: Madagascar, MOZ: Mozambique, MWI: Malawi, PER: Peru, PNG: Papua New Guinea, PRY: Paraguay, SLE: Sierra Leone, TZA: Tanzania, ZMB: Zambia, ZWE: Zimbabwe.

Project ID	Area (Ha)	Starting year	Inclusion status	Undisturbed %	Degraded %	Deforested %	Regrowth %	Water %	Other %
BOL_818	236	2011	<80% undisturbed	17.1	18.9	5.4	48	0	9.93
BLZ_812	676	2009	<80% undisturbed	59.3	16.1	11	0.39	0	12.4
COL_PL1695	2455	2014	<80% undisturbed	47.6	12.8	4.12	0.89	0.01	33.9
PRY_953	4902	2011	<80% undisturbed	0	0	2.24	0	0	97.2
BLZ_647	5477	2005	<80% undisturbed	59.5	25.6	1.1	0.59	0.29	12.3
BRA_832	29862	2007	<80% undisturbed	78.2	10.2	0.11	8.7	0.07	2
PER_PL1455	39792	2013	<80% undisturbed	0.01	0.02	0.2	0	0	96.2
ZMB_1202	40334	2009	<80% undisturbed	0	0	0	0	0	0
IDN_674	47574	2008	<80% undisturbed	52.7	26.5	10.8	1.65	0.19	7.42
KHM_904	63220	2008	<80% undisturbed	19	2.76	4.97	0.01	3.45	69.3
TZA_1325	66528	2010	<80% undisturbed	0.01	0.03	2.17	0.01	0.01	95.9
LAO_1398	142870	2013	<80% undisturbed	50.9	4.35	4.84	0.35	0.35	38.6
MOZ_PL1674	153753	2012	<80% undisturbed	0	0	0	0	0	0
KHM_1650	173280	2010	<80% undisturbed	32.9	4.71	4.28	0.32	0	57.2
KEN_612	175371	2010	<80% undisturbed	0.01	0.02	0	0	0.01	99.1
GTM_1541	204980	2012	<80% undisturbed	42.2	8.83	40.5	0.82	0.74	6.36
MWI_1168	218647	2009	<80% undisturbed	1.31	0.9	0.38	0.07	0.23	96.5
ETH_1340	256878	2012	<80% undisturbed	48.5	14.3	22.6	0.54	0	17.9
KEN_1408	416189	2013	<80% undisturbed	0.6	0.08	0.04	0	0.19	97.8
GTM_1384	726388	2012	<80% undisturbed	60.5	11.7	6.22	0.59	0.07	19.8

Project ID	Area (Ha)	Starting year	Inclusion status	Undisturbed %	Degraded %	Deforested %	Regrowth %	Water %	Other %
ZWE_902	1075623	2011	<80% undisturbed	0	0	0	0	0	0
ZMB_1532	1426030	2012	<80% undisturbed	0	0	0	0	0.11	91
PER_1133	3020	2010	Included for matching	98.6	0.54	0.06	0.08	0.02	0.07
BLZ_1326	8723	2011	Included for matching	93.8	5.14	0.03	0.14	0	0.31
PER_PL1218	8724	2011	Included for matching	93.2	3.14	2.1	0.42	0.05	0.49
COL_856	10687	2010	Included for matching	95.7	2.97	0.12	0.02	0	0.53
BRA_1113	29195	2011	Included for matching	93.6	1.92	2.31	0.73	0.09	0.68
BRA_1329	30242	2012	Included for matching	92.2	2.03	0.21	0.37	0	0.24
BRA_1118	33626	2009	Included for matching	84.7	3.31	4.93	0.3	0	1.88
BRA_963	35745	2011	Included for matching	95.3	1.69	0.62	0.05	0.35	0.44
BRA_1382	39749	2012	Included for matching	98.2	0.52	0.11	0.04	0	0.05
COL_1399	40471	2013	Included for matching	96	0.93	0.02	0.03	0	0.05
BRA_1112	42788	2011	Included for matching	96.9	0.86	0.83	0.38	0.09	0.26
BRA_PL1654	47327	2013	Included for matching	96.8	0.71	0.44	0.03	0.52	0.85
COL_1391	54210	2013	Included for matching	97.3	0.83	0	0.04	0.2	0.18
COL_1389	55448	2013	Included for matching	88.1	3.73	0.39	0.67	0.83	1.15
COL_1396	61132	2014	Included for matching	91.8	1.17	0.03	0.01	0.06	0.18
COL_1400	64997	2013	Included for matching	96.7	0.75	0	0.02	0.16	0.25
COL_1392	65845	2013	Included for matching	97.2	0.62	0.02	0.41	0.31	0.15
SLE_1201	70649	2012	Included for matching	97.2	1.47	0.04	0.03	0.01	0.26
BRA_875	72120	2009	Included for matching	97.3	1.42	0.42	0.08	0.01	0.1
BRA_1571	73620	2013	Included for matching	97.4	1.29	0.14	0.1	0.01	0.01
BRA_1115	78232	2011	Included for matching	84.8	2.68	6.35	0.32	0.08	5.09
COL_1395	91971	2013	Included for matching	96.4	1.28	0.08	0.08	0.07	0.17
COG_1052	92735	2012	Included for matching	99.3	0.01	0	0	0	0
PER_844	98539	2009	Included for matching	90.9	7.26	0.93	0.09	0.01	0.3
BRA_1094	99149	2002	Included for matching	97.3	1.37	0.26	0.03	0.1	0.25
BRA_1503	101592	2012	Included for matching	95.8	1.16	0.42	0.11	0.16	0.13
PER_1360	129260	2010	Included for matching	95.9	1.45	0.39	0.15	0.06	0.34
COL_1390	134243	2014	Included for matching	90.9	3.99	0.49	0.03	1.15	1.65
MDG_1047	144335	2007	Included for matching	85.1	3.65	3.75	0.08	0	1.04

Project ID	Area (Ha)	Starting year	Inclusion status	Undisturbed %	Degraded %	Deforested %	Regrowth %	Water %	Other %
BRA_981	150204	2008	Included for matching	93.9	2.91	1.39	0.15	0.12	0.81
IDN_1477	150715	2010	Included for matching	96.4	1.82	0.74	0.26	0	0.07
PER_944	178704	2007	Included for matching	88.8	4.81	1.44	0.3	0.07	3.96
BRA_1686	187740	2014	Included for matching	98.2	0.92	0.14	0.01	0	0.03
COD_1359	199421	2009	Included for matching	92.2	1.4	0.15	0	0	0.08
BRA_977	211131	2008	Included for matching	90.4	1.11	0.26	0.04	0.12	0.2
COD_934	279650	2011	Included for matching	83	2.06	1	0.02	0.02	2.69
PER_958	3e+05	2010	Included for matching	94.3	3.43	0.55	0.3	0.02	0.69
MDG_1215	374708	2005	Included for matching	89.6	6.93	0.45	0.03	0.06	2.35
MDG_1311	392643	2008	Included for matching	88.9	3.14	1.7	0.06	0	0.2
KHM_PL1748	5e+05	2015	Included for matching	82	5.08	3.02	0.56	0.48	8.25
PER_1067	565849	2010	Included for matching	94.2	1.62	0.25	0.26	1.08	1.07
PNG_1122	653933	2009	Included for matching	96	0.68	0.15	0.03	1.49	0.98
PER_985	1362723	2008	Included for matching	96.7	1.77	0.18	0.13	0.07	0.46
COL_1566	1557190	2013	Included for matching	82.9	2.36	1.05	0.35	1.21	11.4

Table S5: Summaries of project characteristics by selection stage. Countries listed with their International Organization for Standardization system (ISO3): BLZ: Belize, BOL: Bolivia, BRA: Brazil, COD: Democratic Republic of the Congo, COG: Republic of the Congo, COL: Colombia, ETH: Ethiopia, GTM: Guatemala, IDN: Indonesia, KEN: Kenya, KHM: Cambodia, LAO: Laos, MDG: Madagascar, MOZ: Mozambique, MWI: Malawi, PER: Peru, PNG: Papua New Guinea, PRY: Paraguay, SLE: Sierra Leone, TZA: Tanzania, ZMB: Zambia, ZWE: Zimbabwe.

Stage	Area (Ha) mean (IQR)	ISO3 (count)	VCS methodology (count)
All projects (n=71)	201874.4 (39770.4–193580.45)	BRA: 16, COL: 12, PER: 9, BLZ: 4, KHM: 4, MDG: 4, KEN: 3, COD: 2, GTM: 2, IDN: 2, ZMB: 2, BOL: 1, COG: 1, ETH: 1, LAO: 1, MOZ: 1, MWI: 1, PNG: 1, PRY: 1, SLE: 1, TZA: 1, ZWE: 1	VM0017: 1, VM0015: 25, VM0011: 1, VM0009: 9, VM0007: 24, VM0006: 11, VM0004: 1
Excl. insufficient temporal records (n=66)	214466.34 (40368.64–203590.38)	BRA: 16, COL: 11, PER: 9, BLZ: 3, KHM: 3, MDG: 3, COD: 2, GTM: 2, IDN: 2, KEN: 2, ZMB: 2, BOL: 1, COG: 1, ETH: 1, LAO: 1, MOZ: 1, MWI: 1, PNG: 1, PRY: 1, SLE: 1, TZA: 1, ZWE: 1	VM0017: 1, VM0015: 24, VM0011: 1, VM0009: 7, VM0007: 22, VM0006: 11, VM0004: 1
Excl. insufficient temporal and <80% TMF undisturbed cover (n=44) i.e. this is the final set of projects taken forward to matching.	201902.61 (46192.68–190660.06)	BRA: 15, COL: 10, PER: 8, MDG: 3, COD: 2, BLZ: 1, COG: 1, IDN: 1, KHM: 1, PNG: 1, SLE: 1	VM0015: 15, VM0011: 1, VM0009: 3, VM0007: 16, VM0006: 9
