# Supplementary material for: Unobserved confounders cannot explain over-crediting in avoided deforestation carbon projects
Source: Nat Ecol Evol. 2026 May 7;10(6):1071–81. doi: 10.1038/s41559-026-03049-7 (PMC13253331; doi:10.1038/s41559-026-03049-7)
Supplement: Supplementary file 3 — Peer Review File [file 41559_2026_3049_MOESM3_ESM.pdf]

nature portfolio

# Peer Review File

# Unobserved confounders cannot explain over-crediting in avoided deforestation carbon projects

Corresponding Author: Professor Julia Jones

Version 0:

Decision Letter:

10th August 2025

*Please ensure you delete the link to your author homepage in this e-mail if you wish to forward it to your co-authors.

Dear Professor Jones,

Your Article, "Unobserved confounders cannot explain over-crediting in avoided deforestation carbon projects" has now been seen by 3 reviewers. You will see from their comments copied below that while they find your work of considerable potential interest, they have raised quite substantial concerns that must be addressed. In light of these comments, we cannot accept the manuscript for publication, but would be very interested in considering a revised version that addresses these concerns.

If you choose to revise your manuscript taking into account all reviewer and editor comments, please highlight all changes in the manuscript text file.

We are committed to providing a fair and constructive peer-review process. Please do not hesitate to contact us if there are specific requests from the reviewers that you believe are technically impossible or unlikely to yield a meaningful outcome.

If revising your manuscript:

* Include a “Response to reviewers” document detailing, point-by-point, how you addressed each referee comment. If no action was taken to address a point, you must provide a compelling argument. This response will be sent back to the referees along with the revised manuscript.

* If you have not done so already we suggest that you begin to revise your manuscript so that it conforms to our Article format instructions at http://www.nature.com/natecolevol/info/final-submission. Refer also to any guidelines provided in this letter.

* Include a revised version of any required reporting checklist. It will be available to referees (and, potentially, statisticians) to aid in their evaluation if the manuscript goes back for peer review. A revised checklist is essential for re-review of the paper.

Please use the link below to submit a revised paper:

Link Redacted

**Note:** This URL links to your confidential home page and associated information about manuscripts you may have submitted, or that you are reviewing for us. If you wish to forward this email to co-authors, please delete the link to your homepage.

If you wish to submit a suitably revised manuscript we would hope to receive it within 6 months. If you cannot send it within this time, please let us know. We will be happy to consider your revision so long as nothing similar has been accepted for publication at Nature Ecology & Evolution or published elsewhere.

Nature Ecology & Evolution is committed to improving transparency in authorship. As part of our efforts in this direction, we are now requesting that all authors identified as ‘corresponding author’ on published papers create and link their Open Researcher and Contributor Identifier (ORCID) with their account on the Manuscript Tracking System (MTS), prior to acceptance. This applies to primary research papers only. ORCID helps the scientific community achieve unambiguous attribution of all scholarly contributions. You can create and link your ORCID from the home page of the MTS by clicking on

‘Modify my Springer Nature account’. For more information please visit please visit <a href="http://www.springernature.com/orcid">www.springernature.com/orcid</a>.

Please do not hesitate to contact me if you have any questions or would like to discuss the required revisions further.

Thank you for the opportunity to review your work.

[redacted]

Reviewer comments:

Reviewer #1 (Remarks to the Author):

Particularly because you come down solidly in favor of your own prior analysis, it is important to explore possible reasons other than unobserved confounders that your results are at odds with the claims made by actors in the carbon market.
One possible reason is selection bias in the sample of projects, which is the same as in Guizar-Coutino et al. (2022) with the sole addition of Yacumama Forest Carbon Project. It would seem more straight-forward to just say that they re-analyze the same sample. Alternatively, they should explain the source of their list of 71 projects (e.g. are these all from the Verra registry? From ID-RECCO? Or their own compilation?), and then exactly how they determined which projects had less than 80% evergreen forest. For example, in Rimba Raya, this must exclude peat swamp forest, and in Cikel, I wonder if it excludes logged forest? Did they use the same delineation of forest cover in 1990 as for the sampling plots? They further narrow their sample by retaining only projects in which they are able to match at least 80% of the treated plots. Here, it would be worth re-stating the methods and criteria used to determine that a plot could not be matched. I suggest that they present a summary of how sample characteristics (at least project size, country, verifier, etc.) change as they narrow the sample, rather than leaving it up to the reader to pick these patterns out of Table S1.
A second possible concern is the definition of the deforestation outcome, and whether/how you match on proximity to previous deforestation, which is often one of the strongest determinants of deforestation. Here, I am confused about your use of the terms degraded, deforested, disturbed, and forest cover lost. You match on distance to degraded areas in the five years before project start and extent of degraded areas at project initiation (year 0) - but it is not clear if “degraded” means degraded forest, or areas degraded by being cleared of forest.

Minor:
Clarify if used forward stepwise selection with pooled data from all countries to generate one model.
In Figure 5, it appears that the half moons on the axes means statistics that are too large to portray given the scale of the graph – please confirm.
In Table S1, the color scheme appears to be from Figure 4 (not Figure 1).
What more can be said about the 5 projects that apparently were under-credited? It would be worth commenting on region, forest type, and methodology employed to estimate the baseline.
The estimated effect of project Peru 1360 is very different in this paper than in the previous Guizar-Cutino paper. Why?
What is meant by “certified avoided deforestation”? Is this carbon credits generated, registered, sold, or retired from the projects?
The authors note that “selling emissions reductions from avoided deforestation can generate hard-to-replace finance and could make a valuable contribution in the short-to-medium term. For such credits to be credible, they must be based on conservative, ex-post evaluations of avoided deforestation” … please comment on what would be required to sell projects based on ex-post evaluations of avoided deforestation. Could this be the “dynamic baselines” now accepted by Verra?

Reviewer #2 (Remarks to the Author):

Reviewer Report
Title: Unobserved confounders cannot explain over-crediting in avoided deforestation carbon projects
General Assessment:
This is an overall very interesting and well-executed paper, and it makes an important contribution to the literature on the integrity of carbon offset markets, particularly in the context of avoided deforestation projects. The authors present a careful and thoughtful empirical analysis that engages directly with concerns around unobserved confounding—a critical issue in the evaluation of additionality and crediting in nature-based carbon markets. The paper is timely and relevant, and I believe it will be of interest to a broad audience in environmental economics, policy, and climate finance.

Before recommending publication, I suggest the authors address two key points:
1. Clarify Framing and Structure in the Introduction
Currently, the introduction leaves the reader wondering how the authors address unobservable confounders, which by definition cannot be directly measured. The strength of the paper lies in its use of the strongest observable confounder to make a logical inference about the plausibility of unobserved bias—this is a compelling approach, and it should be made clearer from the outset.
To this end, I suggest revising the introduction to explicitly state this framing and possibly moving the R.A. Fisher anecdote (currently later in the paper) up front. This could serve as an intuitive preface for how the authors approach the problem and help orient the reader before diving into the empirical details.

2. Express Over-Crediting in Terms of Issued Credits
The finding that avoided deforestation credits are over-credited by a factor of 3.9 is striking. However, it would be even more informative to relate this directly to issued credits rather than hectares, since the latter can vary widely in carbon content. One option would be to use the offset achievement ratio presented in Probst et al. (2024), which may provide a more interpretable frame for understanding the implications of the over-crediting estimates. Connecting the analysis more directly to carbon credits issued would help clarify the practical magnitude of the problem for both researchers and policy audiences.

Reviewer #2 (Remarks on code availability):

Reviewer #3 (Remarks to the Author):

In “Unobserved confounders cannot explain over-crediting in avoided deforestation carbon projects”, Guizar-Coutiño et al. revisit a prior study of the deforestation impacts of REDD+ to highlight the value of sensitivity analyses in ecological and conservation impact evaluations. The paper tries to accomplish two distinct goals: (1) improve understanding of the scale of overcrediting in REDD+ projects; and (2) highlight sensitivity analyses as a useful and practical tool for ecologists to explore the influence of unobserved confounders. I believe both of these make for important contributions in the field. However, I worry that the current paper falls short of these goals. Below I highlight two core concerns about the paper, as well as a longer list of smaller, editorial suggestions.

Potential use of panel methods
While reading the methods, I was somewhat confused about why the authors choose not to pursue any panel methods for their analysis. Based on their description of the “Observational set-up”, it sounds like they have a nice panel dataset that spans pre- and post-treatment periods. However, they seem to discard much of this detailed temporal data, using it in two ways: (a) defining their primary outcome of interest as the mean deforestation rate in the 5 years following project implementation; and (b) using pre-implementation deforestation metrics for matching and as controls. However, prior work by one of the co-authors has shown that matched panel methods better isolate causal effects than matched, cross-sectional comparisons (Ferraro and Miranda 2014). Why didn’t the authors pursue a staggered difference in differences research design?

My sense is that a shift towards a panel model would improve the paper in line with both of the goals I identify in my opening paragraph. For goal (1), the panel methods would provide a more robust method of estimating the causal effects of these REDD+ projects, further strengthening the confidence in the claims about overcrediting. For goal (2), the methodological demonstration of how to apply these sensitivity analyses in panel settings would help researchers who are seeking to integrate sensitivity analyses into modern quasi-experimental research designs. For example, a clear description of how the methods proposed by (Keele et al. 2019) seems like an obvious, and valuable addition if the authors really want to guide ecologists towards more widespread implementation of sensitivity analyses in cutting-edge, quasi-experimental analyses.

One complication that I can imagine emerging if the authors did shift towards a panel research design is that many of the covariates the researchers use seem to be temporally invariant. However, it seems like there might be other several other variables that could serve as useful alternatives including: gridded population data, night lights data, interactions between accessibility or agricultural suitability and global agricultural prices.

As a related aside, I got the impression that some of the methodological decisions were made to maintain comparability to Guizar-Coutiño et al., 2019? For example, I think this applies both to the decision to stick with a matched, cross-sectional research design, as well as some of the sample selection? If this was a response to a comment to the original publication that strategy might make sense. However, I believe the authors could strengthen this paper as a stand-alone contribution if they didn’t feel the need to maintain consistency across these two studies.

Methodological emphasis on matching techniques rather than sensitivity analysis
Main text of the paper really emphasizes that one contribution the authors hope to make is to demonstrate the use of sensitivity analysis in ecological studies. In contrast, the methods seemed to get bogged down in the details of other techniques, rather than emphasizing the details of the sensitivity analysis. For example, the methods section on “matching” is 1088 words, while the (final) section on the sensitivity analysis that represents the core results presented in the paper is only 171 words. This isn’t inherently a bad thing, but I do worry it underscores a bit of a misalignment between the paper’s stated goals, and its execution. I believe the paper could be more successful in advancing the use of sensitivity analyses if it (a) didn’t make the readers work through somewhat unrelated, but technically complex methods on matching or a diversity of model structures; and (b) provided a more comprehensive description of the literature on, and the authors’ implementation of the sensitivity analysis. For example, readers might benefit from a discussion of how sensitivity analyses might differ when attempting to measure model sensitivity when using just a simple OLS with covariate controls for confounders, versus a model that relies upon a combination of matching and covariates.

Smaller editorial comments
Line 52: “unpicking” is an odd word here, maybe “unpacking”?
Line 69: What do the authors mean by “independent studies”? Aren’t some of these supported by REDD+ verification

bodies? And don’t some of the papers have common authors across studies?
Line 81: The authors are a bit vague in how they describe their research design as using “sophisticated statistical methods.” By this point, I’d like to know the specific method they’re using (e.g. matched, cross-sectional comparisons of post-implementation deforestation).
Line 107: typo, remove 1km
Results: This section seems a bit superficial. Can you dig a bit deeper into your results? For example: (a) You ran lots of additional analyses on different model structures – is there anything useful to conclude based on that? (b) From a climate perspective, what we’re really interested about when it comes to REDD+ is probably the overarching program performance vs the performance of each individual project (e.g. I might be willing to accept overcrediting in one project if another project is undercrediting). Can you reflect on your sensitivity analysis to make statements about the crediting performance of the overarching portfolio of projects as opposed to just the individual projects?
Line 111: If you’re introducing this method to ecologists, I think it’d be useful to provide a bit more intuition about how this comparison provides information about potential confounding.
Line 146: Typo, subject verb agreement
Line 169: This list of caveats is entirely focused on the selection bias in your sample of projects. Are there any other caveats you might want to raise about the broader analysis? For example, what caveats emerge from the use of cross-sectional analysis? And, most importantly, what caveats emerge about your use of sensitivity analysis? Are there other observable confounders that you’d ideally like to include?
Line 181: “avoided XX ha of deforestation”
Line 200: May want to re-order figures
Line 213: Typo, “percent”
Line 221: Given this limitation, why do you use the TMF dataset rather than GFC?
Line 222: Why did you need to drop projects starting before 2000? Given that you’re using JRC data, you can go back to 1990.
Line 233: How did you decide upon this cut-off?
Line 235: Why did you do this sub-sampling before matching?
Lines 250 and 264: You have sections on both “confounders” and “covariate set” but seem to use these terms somewhat interchangeably. Can these sections be better integrated? Maybe it’d be useful to differentiate between the covariates included in your matching, and the theoretical set of confounders that you’d be worried might bias your estimates? I think a more complete description of these confounders, both observed and unobserved, and how they’ve been discussed in the prior literature on the drivers of deforestation and causes of bias in REDD+ studies would be a useful addition, given the focus of the paper.
Line 278: Here you motivate your propensity score subclass analysis by describing issues with difference in difference estimators, but my understanding is that you’re not using DiD? This is a bit confusing.
Table S2: Typo, misspelled “area”
Table S2: Additional variables that might be worth including to expand the sensitivity analysis: Night lights as a proxy for income; gridded population; FAO agricultural suitability or potential yields
References
Ferraro, Paul J., and Juan José Miranda. 2014. “The Performance of Non-Experimental Designs in the Evaluation of Environmental Programs: A Design-Replication Study Using a Large-Scale Randomized Experiment as a Benchmark.” Journal of Economic Behavior & Organization 107, Part A (November): 344–65. https://doi.org/10.1016/j.jebo.2014.03.008.
Keele, Luke J., Dylan S. Small, Jesse Y. Hsu, and Colin B. Fogarty. 2019. “Patterns of Effects and Sensitivity Analysis for Differences-in-Differences.” arXiv:1901.01869. Preprint, arXiv, February 1. https://doi.org/10.48550/arXiv.1901.01869.

Version 1:

Decision Letter:

5th February 2026

Dear Julia,

Thank you for submitting your revised manuscript "Unobserved confounders cannot explain over-crediting in avoided deforestation carbon projects" (NATECOLEVOL-25061882A). It has now been seen again by the original reviewers and their comments are below. The reviewers find that the paper has improved in revision, and therefore we'll be happy in principle to publish it in Nature Ecology & Evolution, pending minor revisions to satisfy the reviewers' final requests and to comply with our editorial and formatting guidelines.

If the current version of your manuscript is in a PDF format, please email us a copy of the file in an editable format (Microsoft Word or LaTex)-- we can not proceed with PDFs at this stage.

If you have not done so already, please ensure that you also email us a completed copy of the Reporting summary :

Reporting summary: <a href="https://www.nature.com/documents/nr-reporting-summary.pdf">https://www.nature.com/documents/nr-reporting-summary.pdf</a>

We are now performing detailed checks on your paper and will send you a checklist detailing our editorial and formatting requirements in about a week. Please do not upload the final materials and make any revisions until you receive this additional information from us.

Thank you again for your interest in Nature Ecology & Evolution. Please do not hesitate to contact me if you have any questions.

[redacted]

Reviewer #1 (Remarks to the Author):

This is a very thorough revision that has made the paper even more compelling. My only remaining concern is around the influence of prior and nearby deforestation on new deforestation.

First, I think that there is a typo in the header for Table S3, which says that the table reports “Averages for accessibility, distance to recent deforestation, slope …”

Second, while it is clear that prior deforestation > 20% of project area leads to the exclusion of a project, it is not clear what percent of prior deforestation would lead to the exclusion of a plot. I suggest that the authors specify what they mean by “some” in their explanation of how the plot sample was generated (“We used the extent of forest cover in 1990 as delineated by the TMF to position sampling plots, ensuring that all samples contained at least some portion of undisturbed moist forest cover when building the time-series of forest cover.”)

Third, in their explanation of observed confounders used in matching, they should be clear that short-term and long-term forest disturbance trends are measured within plots (unlike distance to recent forest degradation events). This is clear in Table S2 but could be clarified in the text with just minor editing.

Fourth, I find it surprising that they have not included nearby historical deforestation (e.g. in a buffer around plots) or distance to prior deforestation in their set of matching covariates - that is, they have not used the most obvious variable available to them to account for the contagious spread of deforestation. Perhaps this is because they believe it is strongly correlated with their accessibility measure and therefore unnecessary? I worry that this somewhat undermines their claims in the sensitivity analysis, because an unobserved confounder related to prior deforestation in the immediate region around a project could very well be just as important as accessibility.

Finally, I hope that the authors continue this line of work, including digging into the implications of unobservable confounders having nonlinear relationships with the treatment and outcome variables or interacting with each other. I don’t think that they need to say more in this manuscript, but I suspect that this will be an important future line of inquiry.

Reviewer #3 (Remarks to the Author):

I thank the authors for their detailed response and careful revisions in response to my prior review. The authors have done an excellent job responding to all of my prior concerns. In particular, I believe the new introduction does an excellent job communicating the paper’s two key contributions, and the additional panel models help readers better understand robustness of results across alternate research designs. The resulting paper represents an important contribution, and I think it’d be an excellent fit for Nature Ecology and Evolution. At this point, I only have minor editorial comments that I hope will help the authors finalize the paper for publication.
Line 75: “delivered little” – Expand, little of what?
Line 87: verb tense, should be “replaced”
Line 97: “specifically from” rather than “specially”
Line 105: “our project-level” rather than “project-level our”
Line 118: “relationship between this study” – both the panel results and the cross-sectional results are coming from “this study”. I think it could be useful to specify here that “Our new panel estimates help explain differences between our cross-sectional results and the results from Guizar-Coutino et al.”
Line 133: The nested parenthetical plus and i.e. is a bit hard to follow
Line 166: At some point, it might be informative to provide some description of the unobserved variables that have been proposed by REDD+ supporters.
Line 189: Missing “be”
Line 202: Missing period.
Line 417: I agree with your arguments for focusing your sensitivity analysis upon the cross-sectional estimator rather than the panel estimator. Your third point emphasizing your desire to choose an estimator that gives REDD+ projects the most generous interpretation is particularly compelling given your conclusions. However, I believe the text on lines 417-425 could be further refined to make this a bit more clear to readers.
Figure 4: I think it’s a bit confusing to have the legend for panels a-b appear under panels c-d, and then to have the legend for panels c-d described in the caption.
Figure 5: If I understand correctly, it might be more clear to state “x times strength of strongest observed confounder” rather than “x times strength of observed covariate” in the figure labels and caption. This language is also more similar to what you use in the text (e.g. lines 155 and 194)

**Open Access** This Peer Review File is licensed under a Creative Commons Attribution 4.0 International License, which permits use, sharing, adaptation, distribution and reproduction in any medium or format, as long as you give appropriate credit to the original author(s) and the source, provide a link to the Creative Commons license, and indicate if changes were made.
In cases where reviewers are anonymous, credit should be given to 'Anonymous Referee' and the source.
The images or other third party material in this Peer Review File are included in the article’s Creative Commons license, unless indicated otherwise in a credit line to the material. If material is not included in the article’s Creative Commons license and your intended use is not permitted by statutory regulation or exceeds the permitted use, you will need to obtain permission directly from the copyright holder.
To view a copy of this license, visit https://creativecommons.org/licenses/by/4.0/

Reviewer #1 (Remarks to the Author):

Particularly because you come down solidly in favor of your own prior analysis, it is important to explore possible reasons other than unobserved confounders that your results are at odds with the claims made by actors in the carbon market. One possible reason is selection bias in the sample of projects, which is the same as in Guizar-Coutino et al. (2022) with the sole addition of Yacumama Forest Carbon Project. It would seem more straight-forward to just say that they re-analyze the same sample. Alternatively, they should explain the source of their list of 71 projects (e.g. are these all from the Verra registry? From ID-RECCO? Or their own compilation?), and then exactly how they determined which projects had less than 80% evergreen forest. For example, in Rimba Raya, this must exclude peat swamp forest, and in Cikel, I wonder if it excludes logged forest? Did they use the same delineation of forest cover in 1990 as for the sampling plots? They further narrow their sample by retaining only projects in which they are able to match at least 80% of the treated plots. Here, it would be worth re-stating the methods and criteria used to determine that a plot could not be matched. I suggest that they present a summary of how sample characteristics (at least project size, country, verifier, etc.) change as they narrow the sample, rather than leaving it up to the reader to pick these patterns out of Table S1.

Thank you for these comments. We agree we should have provided more information on the project selection process. We have revised the methods and supplementary information, including adding two new tables to the supplementary information. We deal with each of your points separately below.

**Selection of REDD+ projects included in the analysis**

The main contribution of this paper, which we make clearer in this version, is an illustration of the value of testing the sensitivity of results to unobserved confounders. We use the debate surrounding over-crediting in REDD+ as an illustrative case study to show why testing sensitivity to unobserved confounders should be normal practice in studies drawing causal inference from observational data. For this reason, we use the same database of 71 projects used by Guizar-Coutiño et al 2022. This represented all the "reducing deforestation and degradation" projects located in the tropics listed by VERRA at the time for which project boundaries were available. Because the requirements of the updated methods we apply in this analysis are slightly different, the exact set of projects included in the final analysis is slightly different.

The methods explains the selection of projects.

> ***Selection of REDD+ projects:*** *We used the database of REDD+ projects compiled by Guizar-Coutiño et al.[1], which identified 81 REDD+ projects established in the tropics (Verified Carbon Standard, 2019) and successfully found boundary data for 71 of these. While new projects have been introduced since 2019, and others withdrawn, for consistency with Guizar-Coutiño et al., we analyze the same set of projects. Guizar-Coutiño et al. determined to include sites that encompassed at least 80% of the undisturbed forest class as characterized by TMF's at the project start date, which enabled the identification of standing forest areas from at least 1990 until project start date. The selection criterion was implemented to ensure alignment with VCS avoided deforestation protocols(Shoch et al. 2011), which require that a project's spatial extent encompasses land that has maintained 100% forest cover for at least 10 years prior to the project starting date. The TMF database, whilst constrained to the moist tropics, enables per-pixel tracking of forest trajectories, distinguishing long-standing forests from areas that have been recently degraded or deforested, which enabled the identification*

*of undisturbed forested areas that could be used as potential controls to assess REDD+ effectiveness. We excluded 22 projects with <80% undisturbed forest cover by the time of project implementation (see Table S4). Projects that had been operating for fewer than 5 years or began before 2000 were also excluded, in the later case because these started before REDD+ was established as an international framework. The remaining 44 REDD+ projects were taken forward for subsequent analyzes. Table S5 shows how the characteristics of included projects changes from the 71 to subset of 44.*

More detail of where the candidate list of 71 projects came from is now provided in the Supplementary Information (section: Selection of REDD+ projects):

*Sourcing of REDD+ project boundaries. The database compiled by Guizar-Coutiño et al. (2022) was created using project boundaries sourced from the Verra database in 2019, accessed at \url{http://www.vcsprojectdatabase.org/}, now accessible via \url{https://registry.verra.org/}. 81 projects categorized as 'Reducing Deforestation and Degradation', located in tropical regions (Africa, South-East Asia, Latin America, and Oceania) were listed at the time. Project boundary data was available for the majority of projects in a KML format. The authors contacted project developers directly when either the project boundary file was missing from the database or when the boundary available online did not correspond precisely with that shown in the Project Design Document (PDD). Project boundaries obtained directly from developers included BRA_1118, BRA_1329, and COD_934, leading to a total compilation of 71 boundary files out of the 81 listed projects in the database.*

Table S1 provides a list of the 71 projects, and a description of which projects were or were not included in the earlier paper, and why those that were rejected from this analysis were rejected.

**Definition of evergreen forest.**
We agree this could have been clearer and have thoroughly revised. Following Guizar-Coutiño et al., 2022, and because humid forest can be more reliably detected from remote sensing than other forest types, we constrain our analysis to projects in humid forest and use the Tropical Moist Forest (TMF) data from the European Space Agency as our forest change dataset. As an inclusion criterion, we use projects covered by at least 80% of the *undisturbed* forest class according to the TMF product, at the time the project was implemented. The text on ‘Selection of REDD+ projects) pasted above makes this clearer.

Our new Table S4 provides information about the percentage cover of each forest class for each project at the time of project implementation and details on whether it was included or not in the matching analysis. Please find below a sample of the first 5 five rows.

*Table S4: Project inclusion status and forest cover at the time of project implementation. Of the 71 projects for which we could obtain spatial data, five projects which operated for fewer than five years or commenced before 2000 were excluded from the analyses (BLZ_852, KHM_PL1689, COL_PL1657, KEN_562, MDG_PL1743; not shown below). Of the remaining 66, 22 contained < 80% of 'undisturbed' forest class as defined by the Tropical Moist Forest product (i.e. evergreen or semievergreen forest pixels which have not been disturbed over the full Landsat historical dataset), shown below as '<80% undisturbed forest cover'. The 44 REDD+ projects taken forward for subsequent analyses are indicated as 'Included for*

*matching'. Columns Undisturbed, Degraded, Deforested, Regrowth, Water and Other refer to the TMF forest classes 1-6, respectively, and represent the composition of tropical forest classes within project boundaries at the time of project implementation. Projects are labelled with their Verified Carbon Standard code preceded by their country code using the International Organization for Standardization system: BLZ: Belize, BOL: Bolivia, BRA: Brazil, COD: Democratic Republic of the Congo, COG: Republic of the Congo, COL: Colombia, ETH: Ethiopia, GTM: Guatemala, IDN: Indonesia, KEN: Kenya, KHM: Cambodia, LAO: Laos, MDG: Madagascar, MOZ: Mozambique, MWI: Malawi, PER: Peru, PNG: Papua New Guinea, PRY: Paraguay, SLE: Sierra Leone, TZA: Tanzania, ZMB: Zambia, ZWE: Zimbabwe.*

| Project ID | Area (Ha) | Starting year | Inclusion status | Undisturbed % | Degraded % | Deforested % | Regrowth % | Water % | Other % |
|---|---|---|---|---|---|---|---|---|---|
| BOL_818 | 236 | 2011 | <80% undisturbed | 17.1 | 18.9 | 5.4 | 48 | 0 | 9.93 |
| BLZ_812 | 676 | 2009 | <80% undisturbed | 59.3 | 16.1 | 11 | 0.39 | 0 | 12.4 |
| COL_PL1695 | 2455 | 2014 | <80% undisturbed | 47.6 | 12.8 | 4.12 | 0.89 | 0.01 | 33.9 |
| PRY_953 | 4902 | 2011 | <80% undisturbed | 0 | 0 | 2.24 | 0 | 0 | 97.2 |
| BLZ_647 | 5477 | 2005 | <80% undisturbed | 59.5 | 25.6 | 1.1 | 0.59 | 0.29 | 12.3 |

We also edited Figure 3, changing the label to '<80% undisturbed' to align with this terminology.

**On forest types included in the analyzes**

The TMF provides limited capabilities to differentiate among tropical moist forest types, for instance, it differentiates between mangrove and bamboo dominated forests but does not delineate peat swamp forests specifically. TMF does enable to identify temporal trajectories of forest disturbances in more specific ways than other globally available products such as the Global Forest Change dataset (Hansen et al. 2012). We exploit this characteristic in our approach for identifying potential control forest plots, by characterizing the extent of forest by disturbance categories (disturbed vs undisturbed) at the plot level, in the lead-up to project implementation. This information is carried forward in the next analytical stage by including plot-level disturbance trajectories as matching covariates. In the examples mentioned above, we matched forest pixels using the project-provided boundaries, such that any forested land included in these polygons was examined in our analysis, provided they conform to the moist forest type definition by Vancutsem et al. This could include logged or disturbed forests if such patches were found within project boundaries, but in these cases our matching strategy ensured that these plots were compared to forest plots with a similar distribution of disturbed/undisturbed forest areas. In the Supplementary Information (section: Selection of REDD+ projects), we provide further details about the selection process concerning forest type and how the type of forest coverage at the time of project implementation influenced project selection.

***Selection of projects based on tropical moist forest pixel coverage.*** *To characterize forest cover and forest dynamics we use the Tropical Moist Forest (TMF) maps, which present*

*deforestation and degradation across the humid tropics at 30 m resolution from 1990 to 2019. The 71 REDD+ sites for which boundary data was available encompassed areas of moist and seasonally dry tropical forests, but because the TMF database only includes forest change data for evergreen and semievergreen forest, our analyzes are limited to these forest types. We only include projects which had at least 80% cover of undisturbed forest class in the TMF dataset (i.e., evergreen/semievergreen forest that has not been degraded or deforested) by the project start date. This excluded 22 projects. Five projects which operated for fewer than 5 years or commenced before 2000 were also excluded. We provide a summary of reasons for inclusion or exclusion in Supplementary Table S1 and detail of forest cover in Table S4.*

We provide additional information on the plot-level characteristics collected as part of the data gathering process and we expand in the matching section on how these plot-based estimates are used in the matching section (reproduced below):

***Plot-level estimates of forest cover, forest degradation and deforestation:*** *Annual estimates of forest cover, forest degradation and deforestation were extracted from the TMF database. This database provides a long-term characterization of forest disturbances in the humid tropics on an annual basis spanning the period 1990 - 2019. Our analyses focused on examining temporal patterns of three main forest classes: the undisturbed class, which refer to closed evergreen or semi-evergreen forest areas that have not been degraded or deforested since at least 1990; the degraded class, consisting of short-term disturbances (less than 2.5 years) due to anthropogenic causes, such as selective logging, or from natural causes such as wind storms or fires; and the deforested class, representing a long-term removal of forest cover (Vancutsem et al. 2021). For each year in the 1990 - 2019 period covered in TMF's Annual Change Collection series, we calculated the extent of undisturbed, degraded and deforested forests classes within sampling plots. We derived our outcome of interest - the annual percentage of forest cover lost - as the percentage of undisturbed forest cover loss relative to the extent of undisturbed forest cover six years prior to project implementation. Finally, we generated annual plot-level estimates of proximity to forest degradation events, as characterized by TMF's degraded forest class. Additional details on the generation of plot-level forest metrics are provided in the SM (Observational set-up).*

***Observed confounders used in matching****: We used the full set of observed confounders (based on known key drivers of deforestation*(Busch & Ferretti-Gallon 2023)*; Table S2). These include elevation, slope, accessibility (measured as travel time to cities) distance to recent forest degradation events in the five years before project start, forest disturbance trends measured over both short-term (year −6 to year 0) and long-term (1990 to year 0) periods, which encompassed the extent of degraded and deforested forest as characterized by the TMF's degraded and deforested forest classes (class 2 and 3 respectively), provided in relative and absolute terms. We also included measures of historical undisturbed forest area year −6, and in 1990.*

**Summary of how sample characteristics change by project selection stage**

In response to your query about how sample characteristics change as the sample reduces from the 71 projects for which we had spatial data to the 44 taken forward for matching, we have added Supplementary Table 5, which provides a summary of the project characteristics at each stage of the project selection. This is referenced in the main text:

Table S5 shows how the characteristics of included projects change from the 71 to the 44 taken forward for matching. We reproduce the Table S5 below:

*Table S5: Summaries of project characteristics by selection stage. Countries listed with their International Organization for Standardization system (ISO3): BLZ: Belize, BOL: Bolivia, BRA: Brazil, COD: Democratic Republic of the Congo, COG: Republic of the Congo, COL: Colombia, ETH: Ethiopia, GTM: Guatemala, IDN: Indonesia, KEN: Kenya, KHM: Cambodia, LAO: Laos, MDG: Madagascar, MOZ: Mozambique, MWI: Malawi, PER: Peru, PNG: Papua New Guinea, PRY: Paraguay, SLE: Sierra Leone, TZA: Tanzania, ZMB: Zambia, ZWE: Zimbabwe.*

| *Stage* | *Area (Ha) mean (IQR)* | *Country, by ISO3 code (count)* | *VCS methodology (count)* |
|---|---|---|---|
| *All projects (n=71)* | *201874.4 (39770.4–193580.45)* | *BRA: 16, COL: 12, PER: 9, BLZ: 4, KHM: 4, MDG: 4, KEN: 3, COD: 2, GTM: 2, IDN: 2, ZMB: 2, BOL: 1, COG: 1, ETH: 1, LAO: 1, MOZ: 1, MWI: 1, PNG: 1, PRY: 1, SLE: 1, TZA: 1, ZWE: 1* | *VM0017: 1, VM0015: 25, VM0011: 1, VM0009: 9, VM0007: 24, VM0006: 11, VM0004: 1* |
| *Excl. insufficient temporal records (n=66)* | *214466.34 (40368.64–203590.38)* | *BRA: 16, COL: 11, PER: 9, BLZ: 3, KHM: 3, MDG: 3, COD: 2, GTM: 2, IDN: 2, KEN: 2, ZMB: 2, BOL: 1, COG: 1, ETH: 1, LAO: 1, MOZ: 1, MWI: 1, PNG: 1, PRY: 1, SLE: 1, TZA: 1, ZWE: 1* | *VM0017: 1, VM0015: 24, VM0011: 1, VM0009: 7, VM0007: 22, VM0006: 11, VM0004: 1* |
| *Excl. insufficient temporal and <80% TMF undisturbed cover (n=44) i.e. this is the final set of projects.* | *201902.61 (46192.68–190660.06)* | *BRA: 15, COL: 10, PER: 8, MDG: 3, COD: 2, BLZ: 1, COG: 1, IDN: 1, KHM: 1, PNG: 1, SLE: 1* | *VM0015: 15, VM0011: 1, VM0009: 3, VM0007: 16, VM0006: 9,* |

A second possible concern is the definition of the deforestation outcome, and whether/how you match on proximity to previous deforestation, which is often one of the strongest determinants of deforestation. Here, I am confused about your use of the terms degraded, deforested, disturbed, and forest cover lost. You match on distance to degraded areas in the five years before project start and extent of degraded areas at project initiation (year 0) - but it is not clear if "degraded" means degraded forest, or areas degraded by being cleared of forest.

Thank you. We have edited the text to make the definitions of forest type more explicit. We have edited the methods section of the text and supplementary information, ensuring that we consistently use the term 'forest degradation' when the TMF's degraded forest class is being used, and

'deforestation' when the TMF deforestation class is being used. As for your query above, we generated estimates of distance to recent forest degradation events when conducting matching, taking closest degraded pixel as characterized by the TMF (using their 'degraded forest class'). We've made this clearer in the methods text in the section ***Plot-level estimates of forest cover, forest degradation and deforestation*** which is reproduced above.

And in the Supplementary Information:

> *For each plot, we obtained mean estimates for elevation (meters) and slope (degrees) \citep{jarvis2008HolefilledSRTMGlobe}, travel time to the nearest urban center \cite{weiss2018GlobalMapTravel}, area of degraded forests and distance to the nearest forest degradation event, both as characterized by the TMF's degraded forest class. Forest degradation in the TMF consists of short-term disturbances (<2.5 years) due to anthropogenic causes, such as selective logging, or from natural causes such as wind storms or fire, which substantially but temporarily alter pixel spectral characteristics \citep{vancutsem2021Longterm19902019}. To account for distance to closest forest degradation events, we produced plot-level annual estimates of the distance to the nearest degraded pixel. First, for each pixel covered by the circular plot, we computed pixel-level estimates of the distance to the nearest pixel that had changed its status from undisturbed to degraded, during the observed year, for the period 1990-2019...*

Our changes to text highlighted above and our new Table S4 make the forest cover classes clearer.

Moreover, we have amended the relevant sections of Table S2 clarifying the use of forest degradation in the generation of forest metrics for matching. The changes are shown in the table below.

Table S2 (extracted section)

| ***Covariate*** | ***Rationale for inclusion*** | ***Source*** | ***Format and resolution*** |
|---|---|---|---|
| *Proximity to recent degradation events (Euclidean distance in meters)* | *Proximity to forest disturbances is consistently associated with higher deforestation risk (Busch & Ferretti-Gallon 2023). We computed the average distance to the closest degraded pixel in the lead up to project implementation, as characterized by the TMF's degradation forest class.* | *Own analysis using the JRC Tropical Moist Forests database (Vancutsem et al. 2021).* | *Raster (30 $m^2$)* |
| *Area of forest disturbances (Ha)* | *The risk of deforestation increases when there are cleared or disturbed patches of forest (Busch & Ferretti-Gallon 2023). We calculated the area of forest disturbances within plots by the year the project was started, by combining the extent of degraded and deforested forest as characterized by the TMF's degraded* | *JRC Tropical Moist Forests database (Vancutsem et al. 2021).* | *Raster (30 $m^2$)* |

| | | | |
|---|---|---|---|
| | *and deforested forest classes (class 2 and 3 respectively).* | | |
| *Undisturbed forest cover extent (Ha)* | *We used estimates of plot-level forest cover as characterized by the TMF's undisturbed forest class, taking measurements from 1990 (i.e. the beginning of the TMF records) to post-five years after project implementation.* | *JRC Tropical Moist Forest database (Vancutsem et al. 2021).* | *Raster (30 $m^2$)* |

Minor:
Clarify if used forward stepwise selection with pooled data from all countries to generate one model.

Thank you, we have now clarified in Supplementary Information as follows:

> *To select an appropriate model while avoiding overfitting, we apply forward stepwise selection using the Bayesian Information Criterion (BIC) as the decision rule, performing this model selection process separately for each project.*

In Figure 5, it appears that the half moons on the axes means statistics that are too large to portray given the scale of the graph – please confirm.
Thank you for pointing this out. The issue was that half-moons correspond to instances where the adjusted model generated out-of-range estimates ($-\infty$). We have now replotted Figure 5 and Figure S5, properly flagging these cases.

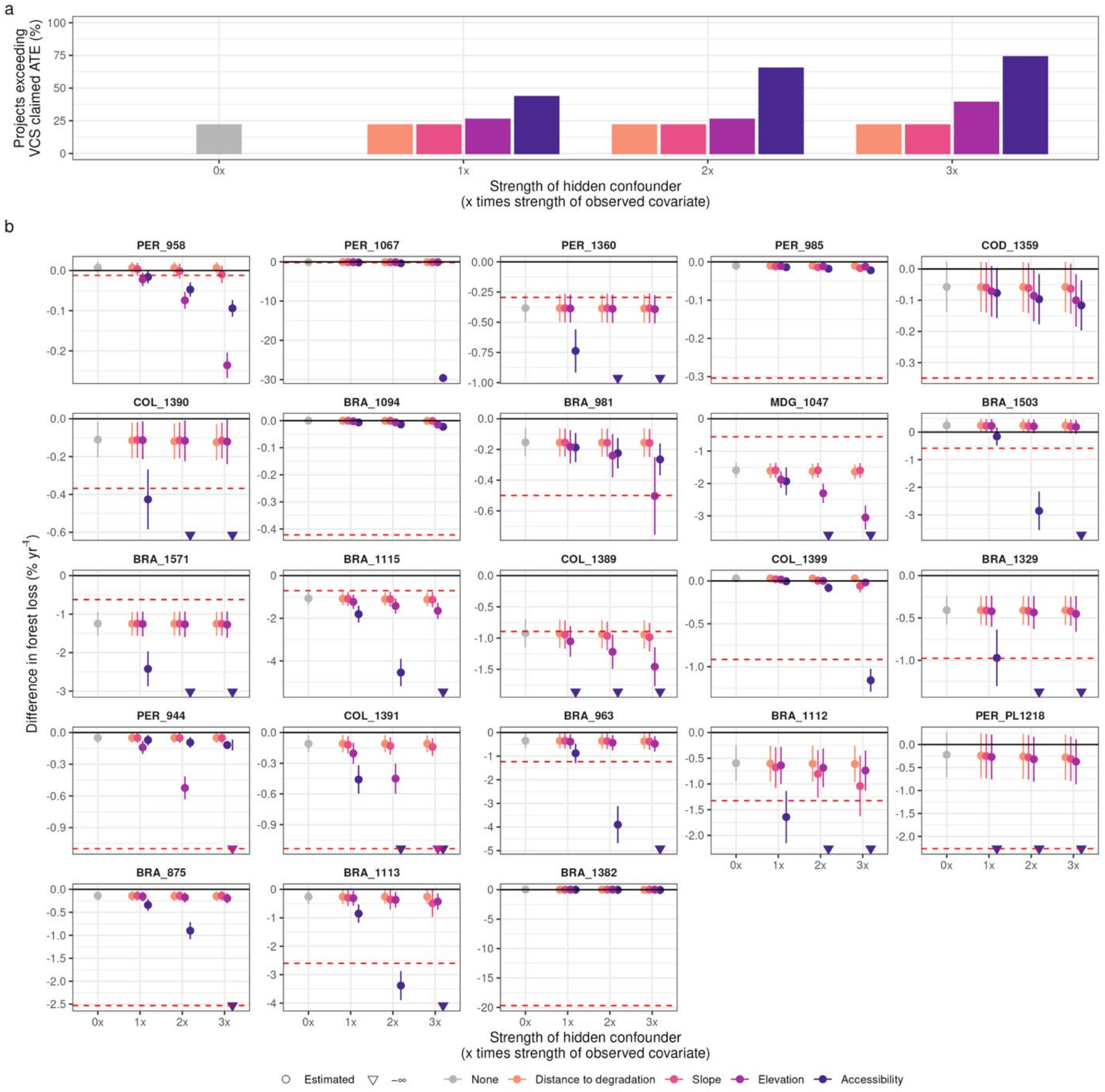


***Figure 5.*** *Sensitivity of our estimates of avoided deforestation to unobserved confounders 1, 2, or 3 times as strong as each measured confounder considered in this analysis. Results are shown for the 23 projects with available data on the avoided deforestation claimed by the project (the projects having red dots in Figure 4c, d).* ***(a)*** *Percentage of projects that reached or exceeded the avoided deforestation claimed by the projects when ATTs are re-calculated considering unobserved confounders 1 to 3 times as strong as measured confounders.* ***(b)*** *Re-estimated ATT values under varying unobserved confounder strengths, with the red dashed line indicating the avoided deforestation claimed by each project. ATT values with out-of-range estimates (−∞) shown in triangles. See Figure S5 for results including projects where <80% of treated plots were successfully matched.*

In Table S1, the color scheme appears to be from Figure 4 (not Figure 1).
Thank you. We have corrected this. The color scheme comes from Figure 3.

What more can be said about the 5 projects that apparently were under-credited? It would be worth commenting on region, forest type, and methodology employed to estimate the baseline.
In the Results section, we have added a clause that mentions the countries where the under-credited projects occur. We have also included the methodology under which projects issued credits to Table S1 and the new Table S5. We do not mention anything about forest type as the TMF dataset we use only covers tropical evergreen and semievergreen and we only include projects which are >80% covered in this forest type at the project start date.

> *For the 23 out of 36 projects for which we have comparable data on certified avoided deforestation, our results suggest substantial over-crediting. (However, it is important to note that five projects (one in Madagascar, two in Brazil, one in Peru and one in Colombia) under-credited i.e. they claimed less deforestation than our results suggest they avoided (Figure 4c, d).) Overall, the projects for which this comparison is possible claimed 3.9 times more avoided deforestation in evaluation and verification reports compared with our estimates (~123,099 ha compared to ~31,822 ha), of which about 50% is explained by two projects alone: BRA_1382 and PER_985, with estimated claimed avoided deforestation areas of 39122.2 and 20684.2 ha, respectively.*

The estimated effect of project Peru 1360 is very different in this paper than in the previous Guizar-Cutino paper. Why?

Thank you for this observant comment. We have added the following explanatory text to the Results section:

> *Our new panel estimates also help explain the relationship between this study and Guizar-Coutiño et al. (2022). The earlier analysis was not a conventional cross-sectional approach: by restricting the sample to plots with zero pre-treatment loss, ATTs were identifiable through within-plot temporal change. This structure is conceptually very close to a fixed-effects panel model. Accordingly, our new panel estimates align closely with the earlier paper, whereas our new cross-sectional estimators (which match on a broader set of time-invariant predictors rather than imposing a flat pre-treatment baseline) yield different effect sizes for some projects, such as PER_1360 (Figure 4).*

What is meant by "certified avoided deforestation"? Is this carbon credits generated, registered, sold, or retired from the projects?
This refers to the number credits verified in the monitoring and evaluation reports. This definition is now clarified the 1st time we mention the certified estimates obtained from project documents. We cite a dataset drawn together by one of our coauthors (Swinfield et al 2025), which includes all the details of how the certified avoided deforestation was compiled for each project.

> *Then we show how sensitivity analyses(Cinelli et al. 2020) can determine the extent to which unobserved confounders in our designs could explain the difference between our estimates and the certified estimates of avoided deforestation from project documents (specially monitoring and evaluation reports(Swinfield et al. 2025)).*

The authors note that "selling emissions reductions from avoided deforestation can generate hard-to-replace finance and could make a valuable contribution in the short-to-medium term. For such credits to be credible, they must be based on conservative, ex-post evaluations of avoided deforestation" ... please comment on what would be required to sell projects based on ex-post evaluations of avoided deforestation. Could this be the "dynamic baselines" now accepted by Verra?

We have updated this text to be clearer:

> *However, for such credits to be credible they must be based on conservative, ex-post counterfactual deforestation estimates (i.e., how much deforestation would have occurred without the REDD+ investment)(Swinfield et al. 2023). While the new jurisdictional REDD+ methodologies (such as ART TREES and VM48), avoid some of the limitations of 1st generation projects by taking responsibility for setting crediting baselines out of the hands of project developers(Haya et al. 2025), they do not yet include such dynamic baselines. A move to dynamic baselines would not change the timing of credit issuance (as existing methodologies only issue credits after ex-post assessment of deforestation in project areas). They would, however, increase uncertainty in the number of credits an investor might expect(Delacote et al. 2025).*

The dynamic baselines introduced by Verra apply only to afforestation, reforestation and revegetation (ARR; VM0047) and improved forest management (IFM; VM0045) interventions. The new REDD+ methodology developed by Verra (VM48) and ART TREEs make use of historical averaging and do not use dynamic baselines.

Reviewer #2 (Remarks to the Author):
General Assessment:
This is an overall very interesting and well-executed paper, and it makes an important contribution to the literature on the integrity of carbon offset markets, particularly in the context of avoided deforestation projects. The authors present a careful and thoughtful empirical analysis that engages directly with concerns around unobserved confounding—a critical issue in the evaluation of additionality and crediting in nature-based carbon markets. The paper is timely and relevant, and I believe it will be of interest to a broad audience in environmental economics, policy, and climate finance.
Thank you for these positive words. We agree!

Before recommending publication, I suggest the authors address two key points:

1. Clarify Framing and Structure in the Introduction. Currently, the introduction leaves the reader wondering how the authors address unobservable confounders, which by definition cannot be directly measured. The strength of the paper lies in its use of the strongest observable confounder to make a logical inference about the plausibility of unobserved bias—this is a compelling approach, and it should be made clearer from the outset. To this end, I suggest revising the introduction to explicitly state this framing and possibly moving the R.A. Fisher anecdote (currently later in the paper) up front. This could serve as an intuitive preface for how the authors approach the problem and help orient the reader before diving into the empirical details.

Thank you. We agree with this suggestion of moving the anecdote. While the applied case study is interesting, the main contribution of the paper is this methodological point: using the strongest observable confounder to make a logical inference about the plausibility of unobserved bias overturning a result. We feel that the new introduction works really well and better emphasizes the methodological contribution of the paper over the applied case study.

2. Express Over-Crediting in Terms of Issued Credits. The finding that avoided deforestation credits are over-credited by a factor of 3.9 is striking. However, it would be even more informative to relate this directly to issued credits rather than hectares, since the latter can vary widely in carbon content. One option would be to use the offset achievement ratio presented in Probst et al. (2024), which may provide a more interpretable frame for understanding the implications of the over-crediting estimates. Connecting the analysis more directly to carbon credits issued would help clarify the practical magnitude of the problem for both researchers and policy audiences.

While in some ways it would be ideal to present over-crediting in terms of carbon rather than hectares, this translation would require substantial assumptions given the information provided by the projects. We also feel that this translation would distract from our main point. Others have written extensively about over-crediting in 1st generation REDD+ projects. While we add to this debate by showing that unobserved confounders are unlikely to explain the discrepancy between what was claimed by projects and what independent studies suggest are more realistic estimates of avoided deforestation, our main contribution is to use the REDD+ case study to show how studies in ecology and conservation that draw causal inference from observational data can, and should, test the sensitivity of their results to unobserved confounders.

Reviewer #3 (Remarks to the Author):

In "Unobserved confounders cannot explain over-crediting in avoided deforestation carbon projects", Guizar-Coutiño et al. revisit a prior study of the deforestation impacts of REDD+ to highlight the value of sensitivity analyses in ecological and conservation impact evaluations. The paper tries to accomplish two distinct goals: (1) improve understanding of the scale of overcrediting in REDD+ projects; and (2) highlight sensitivity analyses as a useful and practical tool for ecologists to explore the influence of unobserved confounders. I believe both of these make for important contributions in the field. However, I worry that the current paper falls short of these goals. Below I highlight two core concerns about the paper, as well as a longer list of smaller, editorial suggestions.

We are glad you appreciate that our goals make important contributions to the field. To address your concerns, we have added substantial new analysis (implementing two fixed-effects panel estimators on the matched dataset) and we have also addressed the smaller editorial suggestions.

**Potential use of panel methods**

While reading the methods, I was somewhat confused about why the authors choose not to pursue any panel methods for their analysis. Based on their description of the "Observational set-up", it sounds like they have a nice panel dataset that spans pre- and post-treatment periods. However, they seem to discard much of this detailed temporal data, using it in two ways: (a) defining their primary outcome of interest as the mean deforestation rate in the 5 years following project implementation; and (b) using pre-implementation deforestation metrics for matching and as controls. However, prior work by one of the co-authors has shown that matched panel methods better isolate causal effects than matched, cross-sectional comparisons (Ferraro and Miranda 2014). Why didn't the authors pursue a staggered difference in differences research design?

My sense is that a shift towards a panel model would improve the paper in line with both of the goals I identify in my opening paragraph. For goal (1), the panel methods would provide a more robust method of estimating the causal effects of these REDD+ projects, further strengthening the confidence in the claims about overcrediting. For goal (2), the methodological demonstration of how to apply these sensitivity analyses in panel settings would help researchers who are seeking to integrate sensitivity analyses into modern quasi-experimental research designs. For example, a clear description of how the methods proposed by (Keele et al. 2019) seems like an obvious, and valuable addition if the authors really want to guide ecologists towards more widespread implementation of sensitivity analyses in cutting-edge, quasi-experimental analyses.

Thank you for your careful reading and insightful comments. We fully agree that a panel framework can complement our matched cross-sectional estimators in that it can adjust for any unobserved time-invariant confounders by exploiting within-plot temporal variation. As part of our response to this comment, we have implemented two fixed-effects panel estimators on the matched dataset (details in Supplementary Information, Section 5): [Model H] a standard two-way fixed-effects model with plot and time fixed effects, and [Model I] an augmented specification including interactions between time and second-level administrative units (the globally available ADM2 layer), which controls for any time-varying confounders at the level of these administrative unit (units such as counties, districts, municipalities, or regions). To identify the average treatment effect on the treated, these models assume that deforestation trends in the treated and control plots would, on average,

be the same in the absence of treatment (i.e., a parallel-trends assumption). Estimation uses the fixest package in R with finite-sample corrections and two-way clustering by plot and time.

We have added these results to the main results (Figure 4).

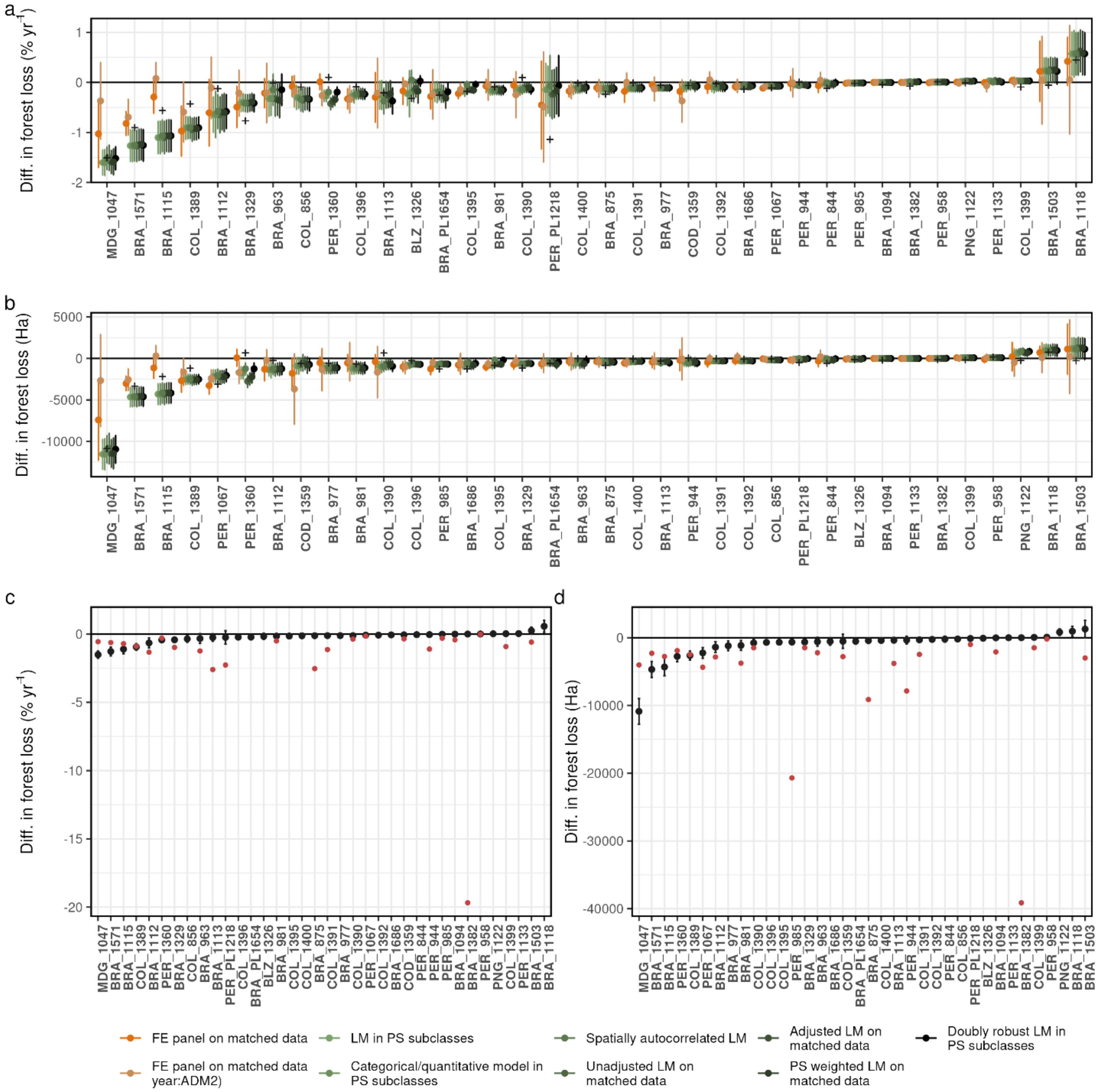


***Figure 4.*** *Average treatment effect on the treated (ATT) for the 36 REDD+ projects which were robustly matched, shown using all matching models as (a) differences in deforestation rates and (b) differences in the area of forest lost. Estimates below the zero line indicate avoided deforestation; error bars represent ±2 SEs. The ATT from Guizar-Coutiño et al. (2022) is marked with a '+'. Panels (c) and (d) show ATT estimates from the doubly robust linear model using propensity score subclassification, in terms of* ***(c)*** *deforestation rates, and* ***(d)*** *forest area lost. Claimed avoided deforestation by projects is shown in red and is available for 23 projects*

*included in the sensitivity analysis (Figure 5). See Figure S4 for results from all 44 projects, including those with <80% of plots matched.*

We have also added text to explicitly compare and contrast the results from the two panel estimators with the results from the estimators in our previous paper, as well as with the results from the seven matched cross-sectional estimators (excerpt below):

> *Thirty-six REDD+ projects were included in our main analysis (33 in the Americas, 2 in Africa and 1 in Asia-Pacific; Figure 3; Table S1). These projects varied in how much deforestation they avoided over their first five years of operation, both in terms of relative reductions in forest loss (Figure 4a) and absolute deforestation avoided (Figure 4b). Across our nine estimators, project-level our estimates of average treatment effect on the treated (ATT) are consistent in their qualitative pattern (Figure 4). While some projects have effect sizes that imply they delivered substantial avoided deforestation (those to the left-hand side of Figure 4), the majority have effect sizes close to zero. Although estimates varied somewhat across models, the overall ranking of projects was remarkably stable. This concordance indicates that our conclusions are not driven by a single modelling choice but are robust across regression adjustment, matching-only approaches, propensity score subclassification, weighting estimators, and fixed-effects panel models.*
>
> *The fixed-effects panel estimators produced ATT estimates that, with few exceptions, were similar to those from our matched cross-sectional models but with wider confidence intervals (Figure 4). In cases where the panel and cross-sectional estimates differed substantially, the panel estimate was closer to zero in the majority of cases, implying that additional control for time-invariant confounding does not reveal systematically larger project impacts.*
>
> *Our new panel estimates also help explain the relationship between this study and Guizar-Coutiño et al. (2022). The earlier analysis was not a conventional cross-sectional approach: by restricting the sample to plots with zero pre-treatment loss, ATTs were identifiable through within-plot temporal change. This structure is conceptually very close to a fixed-effects panel model. Accordingly, our new panel estimates align closely with the earlier paper, whereas our new cross-sectional estimators (which match on a broader set of time-invariant predictors rather than imposing a flat pre-treatment baseline) yield different effect sizes for some projects, such as PER_1360 (Figure 4).*

Our main purpose in this study is to highlight the importance of using sensitivity analyses in causal research, both to strengthen causal claims and to adjudicate disagreements about causal effects. Rather than apply the sensitivity analysis to one of the estimates from the panel data estimators, we argue in favour of applying it to the estimate from the matched and covariate-adjusted, cross-sectional estimator. This cross-sectional estimator yields larger (in absolute value) and more precise ATT estimates than the panel estimators (meaning smaller CIs than the later). It yields more precise estimates because panel data estimators not only eliminate variation in the treatment variable that arises from time-invariant confounders ("bad variation"), but they also eliminate time-invariant sources of variation that have no association with the outcome variable ("good variation"). Thus, using the cross-sectional estimates as the starting point for sensitivity analysis deliberately gives REDD+ projects the best opportunity for their reported avoided-deforestation estimates to align with our estimated effects in Figure 4. In contrast, with the imprecisely estimated effects that are often closer to zero from the panel data estimators, it would be easier for us to make the claim that hidden

biases are not the reason why the estimated effects are so much smaller than the claimed effects. In other words, the panel estimators are relatively disadvantageous towards project claims (when discrepancies from cross-sectional analyses exist), and thus we could be criticized as having deliberately chosen a model that disadvantages the projects in the sensitivity analysis (i.e., a model that would require much larger unobserved confounding to reconcile the empirical estimates with the project-reported avoided deforestation claims). We have added text in Methods to this effect:

> *Sensitivity analysis was implemented using the sensemakr package in R(Cinelli et al. 2020), applied to the matched and covariate-adjusted estimator (Model F, Supplementary Information: Estimating causal impact of projects on deforestation). We selected this estimator as the baseline for three reasons. First, it aligns directly with our core research design, which relies on cross-sectional comparison after matching, while adding robustness through covariate adjustment in the regression stage. Second, it integrates naturally with the Cinelli–Hazlett framework, which quantifies sensitivity in a regression framework in terms of observed covariate strength via partial $R^2$ metrics. Third, and most importantly, the cross-sectional estimator yields larger (in absolute value) and more precise ATT estimates than the panel estimators meaning that this choice deliberately gives REDD+ projects the best opportunity for certified estimates of avoided-deforestation estimates to align with our estimated effects. In contrast, with the imprecisely estimated effects that are often closer to zero from the panel data estimators, it would be easier for us to make the claim that unobserved confounders are not the reason why the estimated effects are so much smaller than the certified effects. In other words, the panel estimators are relatively disadvantageous towards project claims and thus if we were to use them we could be criticized as having deliberately chosen a model that disadvantages the projects in the sensitivity analysis. Full implementation details are provided in the Supplementary Information.*

In response to your comment about alternative sensitivity analysis tools, we have now added text in the Discussion explaining what other methods are available (including Keele et al.), and why we recommend sensemakr applied to matched cross-sectional estimators to ecologists in settings similar to ours:

> *There are a variety of approaches for exploring the sensitivity of estimates of treatment effects to unobserved confounders We considered alternative approaches that have been proposed for observational or panel settings, including methods for two-period panel estimators such as Keele et al(Keele et al. 2019). Oster-type bounds(Oster 2019), E-values* (Van Der Weele & Ding 2017) *and several classical frameworks* (Rosenbaum & Rubin 1983; Imbens 2003; Hosman et al. 2010). *These approaches are not well suited to our design or data structure, and they produce sensitivity measures that are less interpretable in ecological applications (see Supplementary Information for a full explanation). For these reasons, we adopt the Cinelli–Hazlett*(Cinelli & Hazlett 2020) *framework which avoids strong parametric assumptions, provides formally valid bounds grounded in omitted-variable bias theory, and, crucially, produces sensitivity measures directly interpretable relative to the strength of observed confounders.*

*We have also added more detail to the supplementary information:*

> *Panel-based sensitivity methods such as Keele et al. (2019) require explicit assumptions about how unobserved confounding evolves over time, and they quantify bias using a*

*sensitivity parameter Γ, which represents the maximum factor by which an unobserved confounder could differentially influence treatment assignment between periods. Although valuable for two-period or difference-in-differences designs, they cannot be readily interpreted in terms of ecological quantities like variance explained or landscape attributes. Applying the Keele et al. methods would also require restricting identification entirely to within-plot temporal variation, discarding all time-invariant information; in our data, this produces substantially noisier and often smaller estimates, providing an unstable basis for benchmarking unobserved confounding.*

*Oster-type bounds (Oster, 2019) require specifying Rmax (the explanatory power of a hypothetical full model with all confounders) and assuming "proportional selection," meaning that unobserved confounders affect treatment assignment in the same proportion as observed covariates. As noted by Cinelli & Hazlett (2019), this proportional-selection parameter is difficult to interpret and can behave counterintuitively. Given limited pre-treatment data and covariates that cannot plausibly capture most of the variation in deforestation pressure, these assumptions cannot be defended in our ecological setting.*

*The E-value approach (VanderWeele and Ding, 2017) is assumption-light and works with summary statistics, but it is fundamentally defined for risk ratios and most interpretable for binary outcomes. Extensions to continuous outcomes yield quantities without a natural ecological interpretation and cannot be benchmarked against observed covariates, in contrast to partial $R^2$–based sensitivity metrics.*

*For these reasons, we adopt the Cinelli–Hazlett framework. It avoids strong parametric assumptions, provides formally valid bounds grounded in omitted-variable bias theory, and, crucially, produces sensitivity measures directly interpretable relative to the strength of observed ecological covariates.*

One complication that I can imagine emerging if the authors did shift towards a panel research design is that many of the covariates the researchers use seem to be temporally invariant. However, it seems like there might be other several other variables that could serve as useful alternatives including: gridded population data, night lights data, interactions between accessibility or agricultural suitability and global agricultural prices.

Thanks. In the new panel data estimator (which we implemented in response to this reviewer's comments), we do now control for time-varying confounders at the level of these administrative units (such as counties, districts, municipalities, or regions, using the ADM2). Including these time-varying local dummy variables doesn't change the estimates and, as noted above, we don't take panel models forward because they are disadvantageous to the project claims (and therefore it would make it even more difficult for hidden confounders to explain the difference between certified estimates of deforestation and independent estimates). In the context of this paper and the point we are making, we are therefore making the conservative decision.

These results are now reflected in Fig. 4 of the main text and in Figure S4.

In our initial work on this comment we did implement distance to degradation as a time-varying covariate in our panel data analysis that was readily available to us and meaningful. However, upon further thought, we recognised that it could lie on the causal pathway between treatment and outcome, thereby acting as a collider rather than a confounder. REDD+ projects may reduce

degradation pressure in surrounding areas (for example, by deterring agricultural expansion or resource extraction), which in turn increases the distance to the nearest degraded area. Since distance to degradation also predicts future deforestation risk, conditioning on it would block part of the treatment effect and potentially introduce post-treatment bias. In other words, controlling for time-varying post-treatment distance to degradation risks adjusting away a legitimate causal pathway through which REDD+ projects affect outcomes. For this reason, we have not proceeded with reporting results of panel data analysis including this variable.

Of course, it is possible that a panel model with more spatially resolved potential time-varying confounders would yield a different result but unfortunately the suggestions made by the reviewer are not viable. Using either gridded population datasets or night lights directly as proxies for human pressures on forest, do not work well in remote forested areas. For example, if we consider Madagascar, a context we know well, neither dataset reflects human pressures nor are temporal trends necessarily meaningful. The underlying census data at the fokontany level (smallest administrative unit in Madagascar) which feeds into available gridded population datasets such as WorldPop is available for very large time steps with a census in 1993 and the next successful census in 2018. Data is interpolated using night light data and road data, and information on protected areas. However, 11% of the rural population of Madagascar have access to electricity and new night lights appearing, does not necessarily represent increased pressure on forest resources. In fact, it may represent the opposite as an area being electrified may reflect new economics activities beyond subsistence farming. For example, when an illegal gem mining rush occurred in a forested area in eastern Madagascar, night lights would have increased but deforestation actually decreased due to an increase in the opportunity cost of labour, and the fact the gem mining did not require deforestation as they were found in the river bottoms which had been long cleared of forest (Devenish et al. 2024)

There is no appropriate time-varying agricultural suitability layer available at the global scale. While we appreciate the suggestion to make agricultural suitability time varying by interacting with global agricultural prices, this would not be possible without heroic assumptions about the most important agricultural prices driving deforestation in the context of each of the REDD+ projects included in our study. We can illustrate this again with Madagascar. Our sample includes two projects: Coridor Ankeniheny Zahamena in the east and Makira in the north east. What agricultural commodity prices would you include in the model? The main agricultural product in much of the eastern rainforest is rice. However, the link between deforestation rates and national rice prices, much less global rice prices, is extremely weak. This is because very little rice grown in these forest frontier areas is making it into national markets, instead it is mostly for subsistence use or trade in very rural areas. The rate of deforestation in a given area will have much more to do with changes in enforcement of rules which prevent deforestation associated with, for example, the election of a new mayor. In parts of the north west (eastern side of Makira), the main agricultural product is vanilla. However, while the price of vanilla fluctuates dramatically year to year, the link to deforestation is very weak (due to speculation resulting in stock-piling, and the fact that the ‘best’ vanilla is often shade-grown using forest trees as supports for the vanilla vines).

As a related aside, I got the impression that some of the methodological decisions were made to maintain comparability to Guizar-Coutiño et al., 2019? For example, I think this applies both to the decision to stick with a matched, cross-sectional research design, as well as some of the sample selection? If this was a response to a comment to the original publication that strategy might make

sense. However, I believe the authors could strengthen this paper as a stand-alone contribution if they didn't feel the need to maintain consistency across these two studies.

This paper makes two distinct contributions: a) a methodological point that testing sensitivity to hidden confounders is really important when drawing causal inference from observational data, b) insights into the debate about over-crediting in REDD. Following helpful comments from all three reviewers, we have tightened the focus to make it clearer that the main contribution is the methodological point. While the paper will be of interest to anyone following the REDD+ over-crediting scandal (and makes a convincing rebuttal to a critique from industry-linked insiders that over-crediting was just an artifact of hidden confounders), our targeted readership is much broader than this. We believe that by focusing on the same sample of projects as one of the key papers underpinning the over-crediting scandal, the central methodological point that we draw out is clearer. However, because the literature has moved on, we apply very different methods: we sample the landscape using plots not pixels and run a variety of more robust models, including matching with propensity score subclassification, covariate-adjusted estimation, comparisons of post-implementation deforestation across alternative model specifications, and complementary fixed-effects panel analyses.

<u>**Methodological emphasis on matching techniques rather than sensitivity analysis**</u>

Main text of the paper really emphasizes that one contribution the authors hope to make is to demonstrate the use of sensitivity analysis in ecological studies. In contrast, the methods seemed to get bogged down in the details of other techniques, rather than emphasizing the details of the sensitivity analysis. For example, the methods section on "matching" is 1088 words, while the (final) section on the sensitivity analysis that represents the core results presented in the paper is only 171 words. This isn't inherently a bad thing, but I do worry it underscores a bit of a misalignment between the paper's stated goals, and its execution. I believe the paper could be more successful in advancing the use of sensitivity analyses if it (a) didn't make the readers work through somewhat unrelated, but technically complex methods on matching or a diversity of model structures; and (b) provided a more comprehensive description of the literature on, and the authors' implementation of the sensitivity analysis. For example, readers might benefit from a discussion of how sensitivity analyses might differ when attempting to measure model sensitivity when using just a simple OLS with covariate controls for confounders, versus a model that relies upon a combination of matching and covariates.

This is an excellent point. In the revision we have moved much of the detail on matching and modelling to Supplementary Information, and we have added more detail about the sensitivity analysis. Updated text on the sensitivity analysis appears in Results, Methods and Discussion.

Smaller editorial comments

Line 52: "unpicking" is an odd word here, maybe "unpacking"? Change made

Line 69: What do the authors mean by "independent studies"? Aren't some of these supported by REDD+ verification bodies? And don't some of the papers have common authors across studies? These studies are independent in that they are carried out by academics with no links to the

verification bodies or projects themselves. You are correct that some of the papers share co-authors. We have now made it clearer what we mean by "independent."

*These independent analyses are carried out by researchers without links to projects or verification bodies.*

Line 81: The authors are a bit vague in how they describe their research design as using "sophisticated statistical methods." By this point, I'd like to know the specific method they're using (e.g. matched, cross-sectional comparisons of post-implementation deforestation).

Thank you for this suggestion. We have improved the text to read:

*First, we quantify the extent of over-crediting by comparing the avoided deforestation claimed by the projects in certified reports with improved estimates of avoided deforestation from one of the independent studies(Guizar-Coutiño et al. 2022). Improvements in the prior estimates came from two methodological changes aimed at reducing potential sources of bias: sampling deforestation using plots rather than pixels*(Garcia & Heilmayr 2024) *and contrasting multiple estimates from causal inference estimators that more comprehensively control for sources of confounder bias (e.g., matching with propensity score subclassification, covariate-adjusted estimation, and fixed-effects panel analyses). Then we show how sensitivity analyses(Cinelli et al. 2020) can determine the extent to which unobserved confounders in our designs could explain the difference between our estimates and the certified estimates of avoided deforestation. We use this case study to illustrate the wider point about the importance of exploring the sensitivity of results in any causal analysis to the influence of potential unobserved confounders.*

Line 107: typo, remove 1km Edit made

Results: This section seems a bit superficial. Can you dig a bit deeper into your results? For example: (a) You ran lots of additional analyses on different model structures – is there anything useful to conclude based on that?

Thanks for this helpful suggestion. We have now added extra interpretation of concordance and disagreement across models in Results:

*Thirty-six REDD+ projects were included in our main analysis (33 in the Americas, 2 in Africa and 1 in Asia-Pacific; Figure 3; Table S1). These projects varied in how much deforestation they avoided over their first five years of operation, both in terms of relative reductions in forest loss (Figure 4a) and absolute deforestation avoided (Figure 4b). Across our nine estimators, project-level our estimates of average treatment effect on the treated (ATT) are consistent in their qualitative pattern (Figure 4). While some projects have effect sizes that imply they delivered substantial avoided deforestation (those to the left-hand side of Figure 4), the majority have effect sizes close to zero. Although estimates varied somewhat across models, the overall ranking of projects was remarkably stable. This concordance indicates that our conclusions are not driven by a single modelling choice but are robust across regression adjustment, matching-only approaches, propensity score subclassification, weighting estimators, and fixed-effects panel models.*

*The fixed-effects panel estimators produced ATT estimates that, with few exceptions, were similar to those from our matched cross-sectional models but with wider confidence intervals (Figure 4). In cases where the panel and cross-sectional estimates differed substantially, the panel estimate was closer to zero in the majority of cases, implying that additional control for time-invariant confounding does not reveal systematically larger project impacts.*

*Our new panel estimates also help explain the relationship between this study and Guizar-Coutiño et al. (2022). The earlier analysis was not a conventional cross-sectional approach: by restricting the sample to plots with zero pre-treatment loss, ATTs were identifiable through within-plot temporal change. This structure is conceptually very close to a fixed-effects panel model. Accordingly, our new panel estimates align closely with the earlier paper, whereas our new cross-sectional estimators (which match on a broader set of time-invariant predictors rather than imposing a flat pre-treatment baseline) yield different effect sizes for some projects, such as PER_1360 (Figure 4).*

(b) From a climate perspective, what we're really interested about when it comes to REDD+ is probably the overarching program performance vs the performance of each individual project (e.g. I might be willing to accept overcrediting in one project if another project is undercrediting). Can you reflect on your sensitivity analysis to make statements about the crediting performance of the overarching portfolio of projects as opposed to just the individual projects?

Of course, if over-crediting were fully balanced by under-crediting, then this would not affect the ultimate impact on the climate. However, looking across the portfolio, over-crediting was much more common than under-crediting and our analysis shows that hidden confounders cannot explain away this finding.

In addition, buyers buy credits from specific projects, and the claims are at the level of individual projects rather than across all projects trading on the voluntary carbon market. Therefore our analysis by project is appropriate.

Our adapted text is clearer about the existence of under crediting (but that over-crediting dominates).

*For the 23 out of 36 projects for which we have comparable data on certified avoided deforestation, our results suggest substantial over-crediting. (However, it is important to note that five projects (one in Madagascar, two in Brazil, one in Peru and one in Colombia) under-credited i.e. they claimed less deforestation than our results suggest they avoided (Figure 4c, d).) Overall, the projects for which this comparison is possible claimed 3.9 times more avoided deforestation in evaluation and verification reports compared with our estimates (~123,099 ha compared to ~31,822 ha), of which about 50% is explained by two projects alone: BRA_1382 and PER_985, with estimated claimed avoided deforestation areas of 39122.2 and 20684.2 ha, respectively.*

Line 111: If you're introducing this method to ecologists, I think it'd be useful to provide a bit more intuition about how this comparison provides information about potential confounding.

Many thanks for this suggestion. We have now included more text to better introduce the sensitivity analysis (Results, Methods and Discussion); here is the directly related introductory text in Results:

*To assess whether omitted variables could plausibly explain discrepancies between our estimated avoided deforestation and the amounts reported by projects, we quantified the potential strength of unobserved confounding using partial $R^2$. For each project, we computed the partial $R^2$ of each measured confounder (distance to degradation, mean slope, mean accessibility, and mean elevation) with treatment assignment and with deforestation outcomes, using unmatched data (see Supplementary Information Figure S2). These statistics represent the proportion of residual variation explained by the measured confounders, conditional on all other covariates, and were used as empirical benchmarks. We then parameterised hypothetical unobserved confounders with partial $R^2$ values equal to one, two, and three times those of each observed confounders, evaluating each confounder–project combination separately to maintain interpretability. Following Cinelli and Hazlett (2019), this analysis quantifies how strong an unobserved confounder would need to be to materially alter our ATT estimates.*

Line 146: Typo, subject verb agreement

Thank you, edit made.:

*New packages[29] simplify these tests, and we argue that sensitivity analyses should become standard practice whenever ecologists and conservation scientists seek to draw causal inferences from observational data.*

Line 169: This list of caveats is entirely focused on the selection bias in your sample of projects. Are there any other caveats you might want to raise about the broader analysis? For example, what caveats emerge from the use of cross-sectional analysis? And, most importantly, what caveats emerge about your use of sensitivity analysis? Are there other observable confounders that you'd ideally like to include?

Thank you. We have rewritten the section on caveats.

*There are of course important caveats to this work. First, our analysis covers only a subset of the REDD+ projects which were active at the time (Table S1, S5). We could only estimate avoided deforestation for the projects for which there were sufficient temporal records, sufficient coverage of undisturbed moist forest at baseline, and for which we could match at least 80% of treated plots with control plots (36 out of 71 projects). Further, we could only explore whether unobserved confounders could explain over-crediting in the subset of 23 of these 36 projects for which we could obtain data on how much avoided deforestation was claimed by the projects in their project monitoring and evaluation reports(Swinfield et al. 2025). If the included projects systematically differ from the excluded projects, this constraint could limit the generalizability of our conclusions. Secondly, confounders are not the only potential source of bias in the independent studies. Model misspecification biases* (Keele & Grieve 2025) *could also account for the discrepancy between the certified estimates of avoided deforestation and those from the independent studies. Thirdly, spillovers, also called interference among units, could also affect inferences in the independent study designs (Ferraro et al. 2019), but such spillovers would be more likely to inflate these estimates of avoided deforestation rather than deflate them because REDD+ projects are more likely to displace deforestation outside the project area than to reduce it. Finally, all*

*approaches to sensitivity analyses rely on assumptions that may be violated in empirical contexts. Most of the assumptions underlying the Cinelli and Hazlett approach that we use are plausible in our study context, but our conclusions about sensitivity to hidden confounders could change if the effect of the observable and unobservable confounders have nonlinear relationships with the treatment and outcome variables or if the confounders interact with each other.*

We don't go into the caveats that arise from cross-sectional analysis as the main caveat here (omitted variable basis) is what the whole paper is framed around.

Line 181: "avoided XX ha of deforestation"

Thank you, we corrected the sentence to:

> Across the 36 projects for which we have reliable data, the projects avoided 33,948 ha of deforestation

Line 200: May want to re-order figures
We have looked closely at this but not made a change.

Line 213: Typo, "percent"
Thank you, edits made.

Line 221: Given this limitation, why do you use the TMF dataset rather than GFC?
Both the Global Forest Change dataset (Hansen et al) and the TMF dataset are excellent global deforestation layers, but of course like all layers they have their pros and cons. One of the main motivations for using TMF instead of GFC is its ability to characterise per-pixel forest transitions, including repeated disturbance event, from long-standing undisturbed to degraded and deforested classes on an annual basis. This feature makes it ideal for REDD+ project evaluation, as it allows the identification of long-standing forested areas from the wider landscape that remain undisturbed by the time of project implementation, which is a condition that VCS project proponents must follow when defining a REDD+ project area. We've made this fact more explicit in the main text:

> *Guizar-Coutiño et al. determined to include sites that encompassed at least 80% of the undisturbed forest class as characterised by TMF at the project start date, which enabled the identification of standing forest areas from at least 1990 until project start date. The selection criterion was implemented to ensure alignment with VCS avoided deforestation protocols (Shoch et al 2011), which require that a project's spatial extent encompasses land that has maintained 100% forest cover for at least 10 years prior to the project starting date. The TMF database, whilst constrained to the moist tropics, allows for per-pixel forest trajectories separating long-standing forests from recently degraded or deforested areas, which enabled the identification of undisturbed forested areas that could be used as potential controls to assess REDD+ effectiveness.*

Line 222: Why did you need to drop projects starting before 2000? Given that you're using JRC data, you can go back to 1990.

We dropped projects starting before 2005 since this is the year when REDD+ framework became formally adopted as an international forest finance mechanism. We've edited the main text to read as:

> *Projects that had been operating for fewer than 5 years or began before 2000 were also excluded, in the later case because these started before REDD+ was established as an international framework.*

Line 233: How did you decide upon this cut-off?
The choice of threshold was based on previous studies. Local leakage assessments typically focus on areas adjacent to the intervention, normally using up to a 10km radius to examine these patterns (Fuller et al. 2019; Ford et al. 2020; Devenish et al. 2022). Leakage of course can still take place at much larger distances through market forces, displacing deforestation to distant parts within the same country or across borders, but is considerably harder to account for. We added a reference which supports our choice of buffer.

Line 235: Why did you do this sub-sampling before matching?

Sample selection from the full pool of untreated observations at the country level was conducted primarily to reduce spatial dependence in the potential control group. This approach also ensured that a comparable number of control observations were retained for the matching analyses across all examined projects, as differences in the extent of tropical moist forest across countries would inevitably lead to uneven numbers of potential control observations available for each project, depending on the country in which they are located.

Lines 250 and 264: You have sections on both "confounders" and "covariate set" but seem to use these terms somewhat interchangeably. Can these sections be better integrated? Maybe it'd be useful to differentiate between the covariates included in your matching, and the theoretical set of confounders that you'd be worried might bias your estimates? I think a more complete description of these confounders, both observed and unobserved, and how they've been discussed in the prior literature on the drivers of deforestation and causes of bias in REDD+ studies would be a useful addition, given the focus of the paper.

Thank you for highlighting this. We have been through carefully and revised the text to refer consistently to confounders when we are talking specifically about variables which we hypothesize have a causal relationship with selection to the treatment and the outcome of interest. We clarify what possible unobserved confounders could be in Figure 1 and in the text below.

> *While unobserved confounders such as tenure security, the presence of local conservation efforts, or agricultural pressures*(Busch & Ferretti-Gallon 2023) *may explain discrepancies in a few projects, they are unlikely to systematically account for the scale of over-crediting observed.*

We use the term covariate as a synonym for predictor when explaining modelling procedures.

Line 278: Here you motivate your propensity score subclass analysis by describing issues with difference in difference estimators, but my understanding is that you're not using DiD? This is a bit confusing.

Thank you for pointing this out. We have now moved mention of the DiD parallel trends assumption into the description of newly introduced panel models in Methods:

> *To complement these cross-sectional estimators, we additionally fit two fixed-effects panel models. The first uses plot and time fixed effects to allow for unobserved but time-invariant confounders, and identifies effects using only : a base model with fixed entity and time effects within-plot temporal variation. This specification relies on the standard parallel-trends assumption that, in the absence of treatment, each plot's deforestation trajectory can be represented as the sum of a plot-specific baseline level and a shared time trend.*

Table S2: Typo, misspelled "area" Thanks! Fixed.

Table S2: Additional variables that might be worth including to expand the sensitivity analysis: Night lights as a proxy for income; gridded population; FAO agricultural suitability or potential yields
In principle these could be included, however for the reasons given above, we do not believe these would be helpful to include as they represent something very different in the different areas included in this study. More importantly, the aim of this manuscript is not to identify all possible confounders. Our point is that unobserved confounders are inevitable in a study such as this. There are inevitable trade-offs between using repeatable, transparent methods at the global scale, and doing more bespoke, site-by-site analysis which can potentially include appropriate covariates for particular contexts. The global-scale studies which have revealed over-crediting were criticized by some in the industry because they may have omitted important confounders. The point of our paper is to highlight that sensitivity analysis (which we argue should be the norm in causal inference from observational data) can help reveal whether these omitted variables could change the result. This point would not be served by adding more covariates to the analysis. As an aside, the only confounders which would reduce the difference between the quasi-experimental estimates and the certified estimates are ones with a positive relationship to both deforestation and chance of a project being located there. The type of covariates suggested above (if they were appropriate to apply in a standard way across all sites) are likely to be positively associated with deforestation, and negatively associated with location of REDD+ projects and so would likely increase the observed difference between the quasi-experimental estimates and the certified estimates.

Reviewer 3 references


Ferraro, Paul J., and Juan José Miranda. 2014. "The Performance of Non-Experimental Designs in the Evaluation of Environmental Programs: A Design-Replication Study Using a Large-Scale Randomized Experiment as a Benchmark." Journal of Economic Behavior & Organization 107, Part A (November): 344–65. https://doi.org/10.1016/j.jebo.2014.03.008.

Keele, Luke J., Dylan S. Small, Jesse Y. Hsu, and Colin B. Fogarty. 2019. "Patterns of Effects and Sensitivity Analysis for Differences-in-Differences." arXiv:1901.01869. Preprint, arXiv, February 1. https://doi.org/10.48550/arXiv.1901.01869.


**Additional references to support this response.**

Busch J, Ferretti-Gallon K. 2023. What Drives and Stops Deforestation, Reforestation, and Forest Degradation? An Updated Meta-analysis. Review of Environmental Economics and Policy:0.

Devenish K, Desbureaux S, Willcock S, Jones JPG. 2022. On track to achieve no net loss of forest at Madagascar's biggest mine. Nature Sustainability 2022 5:6 5:498–508. Nature Publishing Group. Available from https://www.nature.com/articles/s41893-022-00850-7 (accessed November 16, 2022).

Devenish K, Willcock S, Goodenough KM, Heriniaina R, Rakotonarivo OS, Jones JPG. 2024. No evidence of increased forest loss from a mining rush in Madagascar's eastern rainforests. Communications Earth & Environment 2024 5:1 5:1–12. Nature Publishing Group. Available from https://www.nature.com/articles/s43247-024-01655-6 (accessed September 18, 2024).

Ford SA, Jepsen MR, Kingston N, Lewis E, Brooks TM, MacSharry B, Mertz O. 2020. Deforestation leakage undermines conservation value of tropical and subtropical forest protected areas. Global Ecology and Biogeography 29:2014–2024. John Wiley & Sons, Ltd. Available from https://onlinelibrary.wiley.com/doi/full/10.1111/geb.13172 (accessed December 19, 2025).

Fuller C, Ondei S, Brook BW, Buettel JC. 2019. First, do no harm: A systematic review of deforestation spillovers from protected areas. Global Ecology and Conservation 18:e00591. Elsevier.

Runfola D et al. 2020. geoBoundaries: A global database of political administrative boundaries. PLOS ONE 15:e0231866. Public Library of Science. Available from https://journals.plos.org/plosone/article?id=10.1371/journal.pone.0231866 (accessed December 19, 2025).

Swinfield T, Holland J, Williams AE, Dales M, Guizar Coutiño A, Hartup J, Lam M, Madhavapeddy A, West TAP. 2025. Data for "Understanding the reasons for over-crediting in REDD+." zenodoDOI: 10.5281/ZENODO.15745712. Available from https://zenodo.org/records/15745712 (accessed December 19, 2025).

Swinfield T, Shrikanth S, Bull JW, Madhavapeddy A, Zu Ermgassen SOSE, Affiliations. 2023. Nature-based credit markets at a crossroads. Nature SustainabilityDOI: 10.33774/COE-2023-PL9XV-V2. Available from https://www.cambridge.org/engage/coe/article-details/6576d7e3bec7913d2757aa05 (accessed May 16, 2024).

Verified Carbon Standard (VCS). 2019, December. VCS Project Database.

Reviewer #1 (Remarks to the Author):

This is a very thorough revision that has made the paper even more compelling.

Thank you!

My only remaining concern is around the influence of prior and nearby deforestation on new deforestation.

First, I think that there is a typo in the header for Table S3, which says that the table reports "Averages for accessibility, distance to recent deforestation, slope ..."

Thanks, have changed to "Mean values for distance to populated areas (accessibility) ..."

Second, while it is clear that prior deforestation > 20% of project area leads to the exclusion of a project, it is not clear what percent of prior deforestation would lead to the exclusion of a plot. I suggest that the authors specify what they mean by "some" in their explanation of how the plot sample was generated ("We used the extent of forest cover in 1990 as delineated by the TMF to position sampling plots, ensuring that all samples contained at least some portion of undisturbed moist forest cover when building the time-series of forest cover.")

Thanks. We agree this could be made clearer. We did not explicitly filter sampling plots within project boundaries by prior deforestation rate. Instead, we kept these plots in our observational set and matched them to untreated forest plots with similar prior deforestation rates, so that a) treated samples provide an accurate representation of initial forest condition at the start of the REDD+ projects, and b) matched controls constitute a representative sample of forest plots with similar degree of exposure to deforestation risks than those present in the REDD+ project area. Because our analyses is based on examining avoided deforestation, plots with a starting level of 0% forest cover (due to degradation or deforestation) were excluded. We've made this clear in the following text amendments:

*…We used the extent of forest cover in 1990 as delineated by the TMF to position sampling plots, ensuring that all treated samples contained at least some portion of undisturbed moist forest cover (excluding plots that have been entirely degraded or deforested) when building the time-series…*

Third, in their explanation of observed confounders used in matching, they should be clear that short-term and long-term forest disturbance trends are measured within plots (unlike

distance to recent forest degradation events). This is clear in Table S2 but could be clarified in the text with just minor editing.

Agree this is an excellent point. We've rewritten as:

*… These include elevation, slope, accessibility (measured as travel time to cities) distance to recent forest degradation events in the five years before project start. We also collected key characteristics at the plot-level, such as forest disturbance trends measured over both short-term (year −6 to year 0) and long-term (1990 to year 0) periods …*

Fourth, I find it surprising that they have not included nearby historical deforestation (e.g. in a buffer around plots) or distance to prior deforestation in their set of matching covariates - that is, they have not used the most obvious variable available to them to account for the contagious spread of deforestation. Perhaps this is because they believe it is strongly correlated with their accessibility measure and therefore unnecessary? I worry that this somewhat undermines their claims in the sensitivity analysis, because an unobserved confounder related to prior deforestation in the immediate region around a project could very well be just as important as accessibility.

Thank you. We thought about including this covariate, however it was highly correlated with 2 other variables included in the analyses, namely accessibility and distance to the nearest recently degraded pixel, both of which have been identified as strong predictors of future deforestation (Busch et al 2023) as you rightly point out and which we included.

Finally, I hope that the authors continue this line of work, including digging into the implications of unobservable confounders having nonlinear relationships with the treatment and outcome variables or interacting with each other. I don’t think that they need to say more in this manuscript, but I suspect that this will be an important future line of inquiry.

Thank you for the encouragement and the suggestion.

Reviewer #3 (Remarks to the Author):

I thank the authors for their detailed response and careful revisions in response to my prior review. The authors have done an excellent job responding to all of my prior concerns. In particular, I believe the new introduction does an excellent job communicating the paper’s two key contributions, and the additional panel models help readers better understand robustness of results across alternate research designs. The resulting paper represents an important contribution, and I think it’d be an excellent fit for Nature Ecology and Evolution.

Thank you!

At this point, I only have minor editorial comments that I hope will help the authors finalize the paper for publication.
Line 75: "delivered little" – Expand, little of what? No edit made. The following sentences expand on this point (the detail does not fit in that sentence).
Line 87: verb tense, should be "replaced" Done
Line 97: "specifically from" rather than "specially" Thanks! Good spot.
Line 105: "our project-level" rather than "project-level our" Again-great spot. Thanks for pointing this out.
Line 118: "relationship between this study" – both the panel results and the cross-sectional results are coming from "this study". I think it could be useful to specify here that "Our new panel estimates help explain differences between our cross-sectional results and the results from Guizar-Coutino et al." Agreed, done.
Line 133: The nested parenthetical plus and i.e. is a bit hard to follow. Good spot. Edit made.
Line 166: At some point, it might be informative to provide some description of the unobserved variables that have been proposed by REDD+ supporters. We believe this is covered in the introduction where we say: *"Many in the carbon credit industry strongly refute the findings*[24–26]*. One important critique of these studies is that they inevitably miss important factors that may influence deforestation at the local level, such as the land tenure or emerging local pressures. If these local drivers of deforestation are also associated with where REDD+ projects are established, omitting them from an analysis could indeed underestimate the impact of REDD+ projects."* This is therefore part of the set up and we don't feel it works for the narrative to revisit here.
Line 189: Missing "be" Thanks! Change made.
Line 202: Missing period. Thanks! Change made.
Line 417: I agree with your arguments for focusing your sensitivity analysis upon the cross-sectional estimator rather than the panel estimator. Your third point emphasizing your desire to choose an estimator that gives REDD+ projects the most generous interpretation is particularly compelling given your conclusions. However, I believe the text on lines 417-425 could be further refined to make this a bit more clear to readers. We have made a slight edit to make the point more explicit. Our text now reads:

"Sensitivity analysis was implemented using the sensemakr package in R[29], applied to the matched and covariate-adjusted estimator (Model F, Supplementary Information: Estimating causal impact of projects on deforestation). We selected this estimator as the baseline for three reasons. First, it aligns directly with our core research design, which relies on cross-sectional comparison after matching, while adding robustness through

covariate adjustment in the regression stage. Second, it integrates naturally with the Cinelli–Hazlett framework, which quantifies sensitivity in a regression framework in terms of observed covariate strength via partial $R^2$ metrics. Third, and most importantly, the cross-sectional estimator yields larger (in absolute value) and more precise ATT estimates than the panel estimators meaning that our choice of estimator deliberately gives REDD+ projects the best opportunity for certified estimates of avoided-deforestation estimates to align with our estimated effects. In contrast, with the imprecisely estimated effects that are often closer to zero from the panel data estimators, it would be easier for us to make the claim that unobserved confounders are not the reason why the estimated effects are so much smaller than the certified effects. In other words, the panel estimators are relatively disadvantageous towards project claims and thus if we were to use them we could be criticized as having deliberately chosen a model that disadvantages the projects in the sensitivity analysis. Full implementation details are provided in the Supplementary Information."

Figure 4: I think it's a bit confusing to have the legend for panels a-b appear under panels c-d, and then to have the legend for panels c-d described in the caption. Agreed, we have made a change.

Figure 5: If I understand correctly, it might be more clear to state "x times strength of strongest observed confounder" rather than "x times strength of observed covariate" in the figure labels and caption. This language is also more similar to what you use in the text (e.g. lines 155 and 194). Agreed, we have made the change suggested.
